# Construction of 3D-rendering imaging of an ischemic rat brain model using the planar FMMD technique

**DOI:** 10.1038/s41598-019-55585-x

**Published:** 2019-12-13

**Authors:** Chang-Beom Kim, Sang-Jin Park, Jae-Chan Jeong, Seung-Min Choi, Hans-Joachim Krause, Dae-Yong Song, Hyobong Hong

**Affiliations:** 1 SW Contents Research Lab., Electronics and Telecommunications Research Institute (ETRI), 218 Gajeong-Ro, Yuseong-Gu, Daejeon 34129 Republic of Korea; 2 Department of Anatomy and Neuroscience, School of Medicine, Eulji University, 77 Gyeryong-Ro, Jung-Gu, Daejeon 34824 Republic of Korea; 3 Institute of Complex Systems, Bioelectronics (ICS-8), Forschungszentrum Jülich, Jülich, 52425 Germany

**Keywords:** Imaging techniques, Biomedical engineering, Biomedical engineering, Imaging techniques, Biomedical engineering

## Abstract

Occlusion of the major cerebral artery usually results in brain hypoxic-ischemic injury, which evokes neuroinflammation and microglial activation. Activated microglia are considered a source of multiple neurotoxic factors, such as reactive oxygen species (ROS), in the central nervous system (CNS). We herein present a 3D-rendering brain imaging technique in an experimental rodent model of cerebral ischemia based on 2D magnetic images of superparamagnetic iron oxide nanoparticles (SPIONs) using the planar frequency mixing magnetic detection (p-FMMD) technique. A rat model of cerebral ischemia was established by unilateral middle cerebral artery occlusion with reperfusion (MCAO/R) injury. 2,3,5-Triphenyltetrazolium chloride (TTC) staining was performed to demonstrate the irreversibly damaged ischemic brain tissues, and double immunofluorescent labeling of OX6 (activated microglial marker) and ethidium (ROS marker) was conducted to confirm ROS generation in the activated microglia in the infarcted brain region. The ischemic brain sections treated with OX6-conjugated SPIONs were scanned using our p-FMMD system, yielding 2D images on the basis of the nonlinear magnetic characteristics inherent in SPIONs. The p-FMMD signal images representing microglia activation show an infarct ratio of 44.6 ± 7.1% compared to the contralateral counterpart, which is smaller than observed by TTC (60.9 ± 4.9%) or magnetic resonance imaging (MRI, 65.7 ± 2.7%). Furthermore, we developed a 3D-rendering brain imaging process based on the 2D p-FMMD signal images. The 3D reconstructed model showed a decreased ratio of coincidence of the ischemic regions compared with MRI models. In this study, we successfully conducted a feasibility test on whether our p-FMMD technology, a technique for signaling and imaging based on the nonlinearity of SPIONs, can be used to visualize the ischemic brain region in real time by detecting activated microglia in an MCAO/R animal model. Therefore, our method might allow for a different approach to analyze the pathophysiology of ischemic stroke through molecular imaging. Furthermore, we propose that this magnetic particle imaging (MPI) technique that detects the nonlinear magnetization properties of SPIONs could be applied not only to a stroke model but also to various types of pathophysiological studies as a new bioimaging tool.

## Introduction

Imaging techniques for analyzing and visualizing tissue biospecimens are important in the field of biological research and clinical diagnosis. There have been many efforts and studies in these fields to develop and improve medical imaging techniques and systems. To date, many biological imaging systems and the image analysis process are mainly based on immunolabeling. Immunolabeling is a biochemical method that enables the detection and localization of a particular antigen within a tissue or organ by tagging it with a specific antibody conjugated to marker-like fluorescent molecules or chromogens^1^. To accurately analyze the target biomaterials during experiments, one of the most important factors to be considered is the photostability of the fluorescent dyes or chromogens^2^. However, photobleaching has become a critical factor to overcome^3^. To solve the above problem, quantum dots (Qdots) have recently been employed to complement conventional fluorescent dyes^4^ due to their superior photostability compared to conventional fluorescence dyes. However, Qdots also have intrinsic problems, such as photoblinking that induces fluorescence intensity fluctuations^5^, cytotoxic damage to cell functionalities^6^, and a blueshift after continuous excitation leading to a photobleached state^7^. Additionally, in the case of fluorescent markers, light needs to be able to pass through the tissue. Thus, alternative methods have been explored that are less susceptible to photobleaching to allow longer duration excitation of fluorescent markers.

The magnetic particle imaging (MPI) technique, which uses magnetic nanoparticles (MNPs) as imaging tracers, has been developed as one of several promising medical imaging techniques. Among various types of MNPs, superparamagnetic iron oxide nanoparticles (SPIONs) have been used in a wide variety of applications due to their high coercivity, high magnetic saturation, and high susceptibility, which are physicochemically steady with prolonged occurrences of redox reactions^8^. In particular, the applications of SPIONs have been expanding to the detection of specific biological substances, magnetic bioseparation, therapy, and even drug delivery. Currently, SPIONs are now being used in medical imaging technology fields such as MRI and MPI^9–12^. MPI technology is a novel medical imaging technique based on the nonlinear magnetization properties of SPIONs when placed under external AC magnetic fields. To date, many studies have shown that MPI is an emerging technology that exhibits feasibility for highly sensitive and highly spatially and temporally resolved medical imaging^13–15^.

Our previous research showed the feasibility of 2D MNP imaging of microchannel platforms with our planar frequency mixing magnetic detection (p-FMMD) technique^16^. The p-FMMD technique can be classified as a nonlinear AC susceptometry employing two distinct frequencies. In the p-FMMD measurement, the sample is exposed to a time-varying magnetic field consisting of two spectral components, a high and low frequency. The nonlinearity in the magnetization curve of a sample substance leads to the generation of harmonics and the mixing components of these frequencies. When the MNPs are exposed to the excitation fields with frequencies *f*_1_ and *f*_2_, harmonics with frequencies of *m*·*f*_1_ ± *n*·*f*_2_ (where *m* and *n* denote integers) will be generated^17^. A previous study showed that the measurement of the mixing term *f*_1_ + 2·*f*_2_ is very useful to quantify the total amount of MNPs in a sample^18^.

In this study, we demonstrate the applicability of our p-FMMD device as an *in situ* imaging tool for the detection of microglial activation in the ischemic region in an experimental animal model. The middle cerebral artery occlusion with reperfusion (MCAO/R) injury rat model was employed, and the region damaged by cerebral ischemia was visualized using our p-FMMD system. The efficacy of the p-FMMD system was verified by comparing the images generated from p-FMMD with the resultant images of 2,3,5-triphenyltetrazolium chloride (TTC) staining and OX6 immunofluorescence staining, which are commonly used to identify cerebral ischemic damage. Furthermore, we built a 3D-rendering brain image based on 2D SPION images obtained from our p-FMMD system and compared it with an image from 3D magnetic resonance imaging (MRI). These results demonstrate that our p-FMMD system can successfully visualize the SPIONs embedded in the tissue sections, showing the feasibility of this method as a novel *in situ* imaging tool for various biological specimens.

## Methods

### MCAO/R injury rat model preparation

All experimental procedures were performed according to the National Institutes of Health Guide for the Care and Use of Laboratory Animals (NIH Publication No. 80-23, revised 1996) under the approval of the Eulji University Institutional Animal Care and Use Committee. Adult male Sprague-Dawley rats (body weight 250–300 g, Charles River Lab., DE, USA) were housed with freely available food and water at a constant room temperature (20–22 °C) on a 12:12 hour light-dark cycle.

Following the surgical procedure^19^ (see Supplement S1), a cerebral ischemic rat model was established by deliberately inducing unilateral middle cerebral artery occlusion with reperfusion (MCAO/R) injury. Briefly, experimental animals were anesthetized with ketamine (70 mg/kg body weight) and xylazine (8 mg/kg body weight) intraperitoneally. The external carotid artery (ECA) was isolated and coagulated by identification of the branching superior thyroid artery from the left common carotid artery (CCA). The internal carotid artery (ICA) was also isolated, and an MCAO suture (403965PK10, Doccol Corporation, Sharon, MA, USA) was introduced into the ICA lumen at approximately 25 mm until resistance was felt and a slight curving of the suture was observed. After 1 hour of MCAO, the MCAO suture was withdrawn to allow reperfusion. After postsurgical treatment, the rats were allowed to survive for 1 day and then sacrificed the next day. Transcardial perfusion of fixative, 4% paraformaldehyde for immunofluorescence or phosphate-buffered saline (PBS) for TTC staining, was performed prior to the removal of the brains from the experimental animals.

### TTC staining and measurement of the cortical infarct volume

To examine tissue viability and evaluate the infarct size, TTC staining was conducted. One day after MCAO/R injury, the animals were deeply anesthetized and transcardially perfused with 100 ml of ice-cold saline with 0.5% sodium nitrite and 10 U/ml heparin. The rat brains were rapidly extracted and sliced into 2 mm thick coronal sections using a Rat Brain Blocker (David Kopf Instruments, CA, USA). The coronal sections at the level of caudoputamen where the anterior commissure was observed (approximately 3 mm caudal from the bregma) were incubated in a 2% solution of 2,3,5-triphenyltetrazolium chloride (TTC; Sigma-Aldrich Inc., MO, USA) for 10 min at 37 °C and fixed with 10% formalin solution after staining. After taking a photograph with a digital camera (NX200, Samsung, Seoul, Korea), ImageJ (Ver 1.45) software (http://rsb.info.nih.gov/nih-image) was used for infarct volume analysis, as described previously^19^. The size of the infarct volume (n = 5) was measured as follows,

1 $${V}_{infarct}( \% )=\frac{{A}_{contra}-({A}_{ipsi}-{A}_{infarct})}{{A}_{contra}}\times 100$$

where *V*_*infarct*_ is the infarct volume of the brain, *A*_*contra*_ is the area of the contralateral brain, *A*_*ipsi*_ is the area of the ipsilateral brain, and *A*_*infarct*_ is the area of the infarct brain. We also measured the infarct volumes with p-FMMD (n = 5) and T2 MRI (n = 5). Statistical significance was assessed by independent Student’s t-test and one-way ANOVA followed by Dunnett’s test. Data are represented as the mean ± standard error of the mean (SEM). Values of *p* < 0.05 were considered significant.

### HEt histochemistry and immunofluorescence labeling with SPIONs

The *in situ* visualization of the reactive oxygen species (ROS, O^−^_2_ and O^−^_2_-derived oxidant) generated in the ischemic brain tissues was assessed by hydroethidine (HEt) histochemistry as described previously^20^. One day after MCAO/R injury, HEt (2 mg/kg in saline containing 1% dimethyl sulfoxide; Molecular Probes, Eugene, USA) was intraperitoneally administered. After 30 min, the whole rat brain was removed, and serial coronal sections of 40 μm thickness were obtained using a cryostat microtome (Leica Microsystems Inc., Wetzlar, Germany) (see Supplement S2). The sections mounted on gelatin-coated slides were examined to investigate the accumulation of ethidium (ET), the oxidized product of HEt, using a wide-field fluorescence microscope (400×; excitation 510 nm, emission 580 nm; Olympus, Tokyo, Japan).

Superparamagnetic iron oxide nanoparticles (SPIONs; size 100 nm, number of particles 1.8 × 10^15^/g), the magnetic tracer for our p-FMMD system, were covalently conjugated to OX6 antibodies (mouse monoclonal anti-rat MHC class II, 1:50, Secrotec, Oxford, UK) that reacted with the activated microglia using the carbodiimide method (see Supplement S3). Following incubation in PBS containing 10% normal goat serum for 30 min, the tissue specimens were incubated with anti-OX6 antibody-conjugated SPIONs (1 mg/ml final concentration) for 16 hours at 4 °C using a vertical rotator (20 rpm). After rinsing with PBS, an FITC-conjugated donkey anti-mouse secondary antibody (1:200, Jackson ImmunoResearch Lab., West Grove, USA) was applied for 2 hours at room temperature. All procedures were conducted in the dark.

### The planar frequency mixing magnetic detection (p-FMMD) system

**Figure 1:**
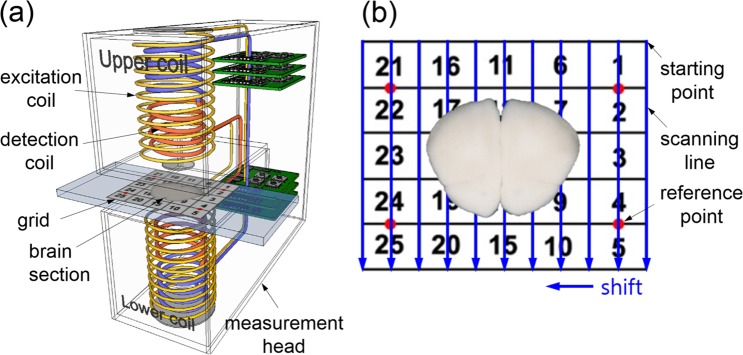
(**a**) Schematic diagram of the p-FMMD system consisting of a magnetic measurement head with an intermediate scanning space between the upper and lower coils. Each part contains a set of excitation coils to generate high and low frequency magnetic excitation and detection coils to detect the nonlinear response signals from the magnetized OX6-conjugated SPIONs. (**b**) A 40 μm thick brain section specimen on a grid platform. The p-FMMD system performs the scanning of the brain section treated with OX6-conjugated SPIONs (invisible) in the infarcted region. Arrows indicate the scanning direction and shifting during the translational motion of the p-FMMD system (the x/y-direction motorized translation stages are not shown).

The measurement head of the p-FMMD system (x/y-direction motorized translation stages not shown) is schematically sketched in Fig. 1(a). Details on this setup can be found in our previous publications^12,21^. For p-FMMD image acquisition, the 40 μm thick coronal brain sections mounted on gelatin-coated slides were covered with coverslips using vector-shield medium (Vector Labs., CA, USA) and scanned within the 2 mm high scanning space between the upper and lower measurement coils.

As shown in Fig. 1(b), a 5 × 5 rectangular grid measuring 3 cm by 2.4 cm is marked on the slide. Each individual grid measures 0.6 cm by 0.48 cm. The rectangles are numbered from 1 to 25. Four reference points are marked in red on the midpoints between two grids (1 and 2, 4 and 5, 21 and 22, and 24 and 25). On these points, SPION droplets were placed, serving as adjustment points for merging the optical images of the brain sections and the p-FMMD scanned measurement data. The brain sections incubated with OX6-conjugated SPIONs were loaded onto the 3 × 3 grid in the center, as schematically shown in Fig. 1(b). The brain section surface was scanned by the p-FMMD system as the measurement head translated along each scanning line (along the blue arrows, 10 shifts, 0.3 cm step width, from right to left). Every 15th brain section was scanned using the p-FMMD system, and p-FMMD images were obtained. The scanned brain sections were also photographed using a camera (Canon 50D, Conon, Tokyo, Japan) for further image processing. The specificity and reliability of our p-FMMD system for biospecimens was verified, as shown in Supplement S4.

### Image processing

The brain section pictures and the p-FMMD scanned images have different coordinate systems. To merge them into the same coordinate system, the four reference points scanned by the p-FMMD system were adjusted to the optically photographed points. By matching the reference points between the optical image and the scanned image, we can unify the coordinate system and correct distortions of the two images. Once the two images were merged into one coordinate system, the magnetized signal region represented the ischemic infarction lesion (the region where the activated microglia are present) of the MCAO/R brain injury model. To remove unnecessary background from the merged image, the outer border of the brain section was precisely defined, and the histologic image was isolated. Then, the magnetic signal image was extracted from the histologic image, separating it into brain tissue and a magnetic signal image in the same coordinate system. All 2D image processing steps were performed using Adobe Photoshop (version 7.0; Adobe systems Inc., CA, USA).

The segmented images were used to reconstruct a 3D rat brain model by stacking and rendering the above 2D images. MATLAB (MathWorks, Natick, MA) was used as the 3D image processing tool with built-in functions such as *isosurface*, *isocaps*, and *isonormals* (see Supplement S5). The function *isosurface* computes the displayable data of the outer surface by connecting points of the same value on the contour of the rat brain in 629 volume data sets. The function *isocaps* calculates the equivalent curved cross-section geometry from the volume data, allowing display of the inside shape of the volume data. The function *isonormals* is used to create smoother shaped equivalent surface. The patch is composed of polygons by using vertices and faces to be connected to each other by using the data calculated by *isosurface* and *isocaps* and outputs the data to the screen in three dimensions. A p-FMMD image was acquired from every 15^th^ slice, from slices 135 to 345. The p-FMMD images of all 22 slices and the corresponding camera images were matched.

### Magnetic resonance imaging (MRI)

To compare the p-FMMD 3D reconstructed model with an MRI-based 3D model, a rat head-and-neck MRI was acquired using a 4.7 T animal MRI system (BioSpec 47/40; Bruker, Germany) at Korea Basic Science Institute in Ochang, with 72 mm volume and rat brain quadrature surface coils for radiofrequency transmission and reception, respectively. All axial T2-weighted RARE images were acquired with the following parameters: TR = 6400 ms, effective TE = 36 ms, slice thickness = 0.5 mm, FOV = 3.5 × 3.5 cm^2^, matrix size = 256 × 256, number of average = 8, and acquisition time = 20 m 29 s. Among the four MRI methods such as T1-4 repeat, T1-8 repeat, T2-4 repeat, and T2-8 repeat, we decided that the T2-8 repeat method was the most suitable for this study (see Supplement S6). Brain images were collected from 5 experimental animals.

## Results and Discussion

In this study, we demonstrated that our p-FMMD system could detect microglial activation that represents the infarction area in brain tissue specimens of a cerebral ischemia animal model in real time. We interpreted the region where the signals for the SPIONs conjugated to the activated microglia marker OX6 were detected as the infarction area. Although the activated microglia distribution in ischemic brain tissue is what we truly visualized, microglial activation generally occurs in brain infarction regions, thus we considered this area as an infarction area despite the imperfect spatial coincidence between the “infarct” detected by p-FMMD, TTC and MRI. We also developed a 3D-rendering brain imaging process to transform 2D p-FMMD magnetized signals into a 3D model. For the experimental sample preparation, the animal stroke model was induced by temporal MCAO/R (1 hour) using the intraluminal thread method, and the infarcted area was confirmed by TTC staining. The infarcted area was also visualized by immunofluorescence labeling using FITC-conjugated OX6 antibodies, which bind to the major histocompatibility complex (MHC) molecules expressed from the activated microglia. Additionally, we applied HEt histochemistry to ensure ROS generation in the infarcted brain sections.

**Figure 2:**
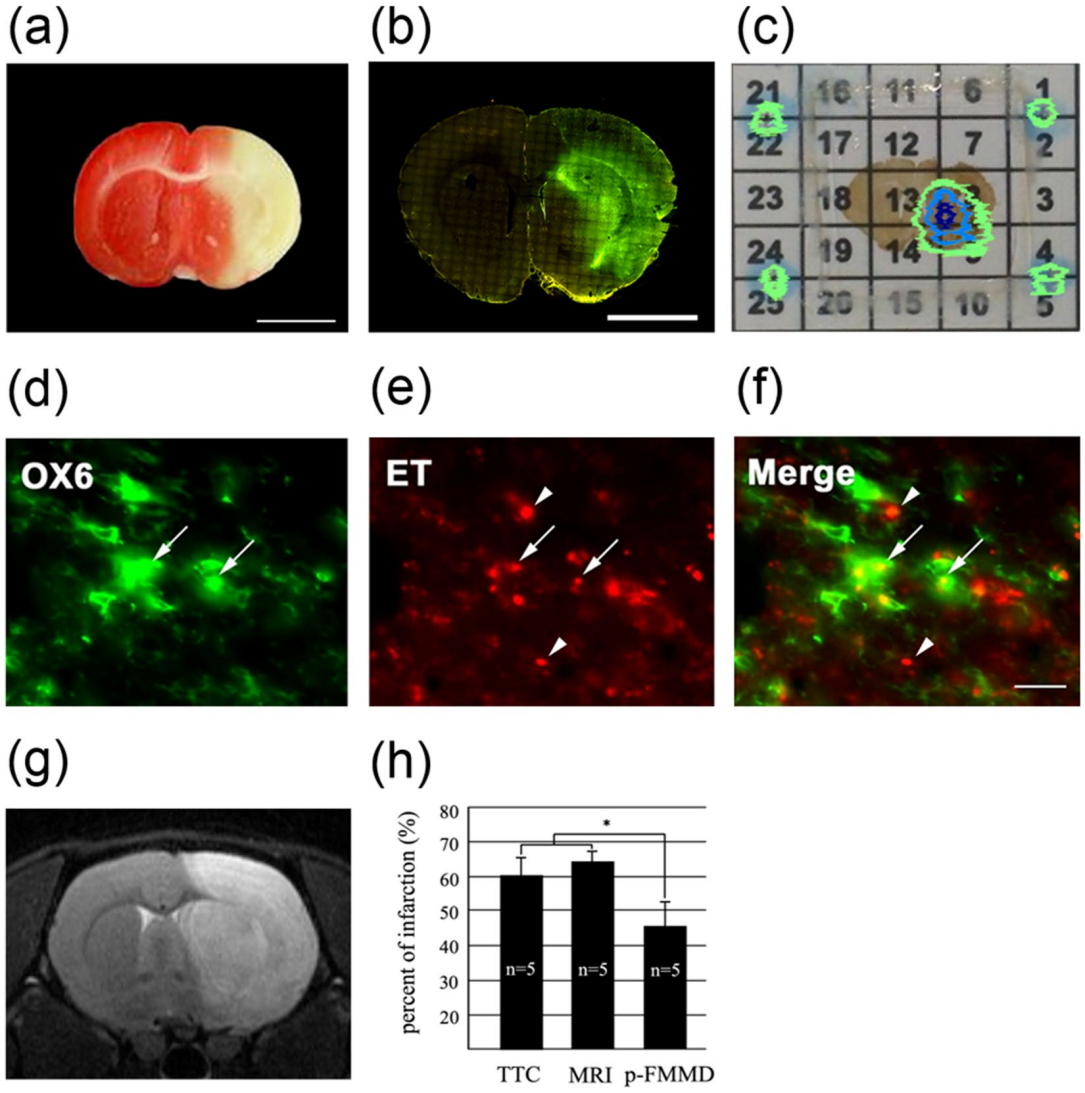
(**a**) Middle cerebral artery occlusion/reperfusion (MCAO/R) model in the adult Sprague-Dawley rat. TTC staining was applied to brain slices 1 day after 1 hour of transient MCAO/R injury. The infarcted area (white) can easily be distinguished from the other intact brain tissue (red). The scale bar represents 5 mm. (**b**) Immunofluorescence labeling of the activated microglia in the infarcted area using antibody OX6 (conjugated with FITC and SPIONs) after MCAO/R injury. The scale bar represents 5 mm. The microglial activation region closely coincides with the TTC stained area on the ipsilateral (right) side of the brain section. (**c**) The p-FMMD signal image of the magnetized OX6-conjugated SPIONs reacting to the activated microglia in the infarcted region. The signal image is merged with a brain section optical image on a grid platform. (**d**) Enlarged immunofluorescent image of FITC-conjugated OX6, a monoclonal antibody reacting to the activated microglia in the ipsilateral region. (**e**) Enlarged immunofluorescent image of ethidium (ET), the oxidized product of hydroethidine (ROS), accumulation in the same brain slice as (**d**). (**f**) The merged immunofluorescent images of OX6 (**d**) and ET (**e**) indicates the spatial coincidence between the activated microglia and ROS generation (arrows). The ET-positive spots (arrowheads) not coinciding with OX6-positive microglia may be caused by dying neurons in the ipsilateral brain parenchyma. The scale bar represents 50 μm. (**g**) T2 MRI at the level of the anterior commissure of a rat brain 1 day after MCAO/R injury. (h) The percent infarct area at the anterior commissure level from TTC staining, MRI, and p-FMMD 1 day after MCAO/R injury. A significantly decreased infarct ratio of p-FMMD compared with that of MRI and TTC was observed. Values are expressed as the mean ± S.E.M. (n = 5). *p < 0.05 represents the statistical significance of the ischemic lesion volume in p-FMMD compared with that in TTC/MRI.

TTC staining was performed to differentiate between the metabolically active and inactive brain regions in our animal MCAO/R model. TTC is a redox indicator that is reduced to a red formazan by mitochondrial enzymes in metabolically active cells. TTC staining has been widely used as a reliable histochemical indicator to experimentally detect ischemic brain damage^22,23^. As shown in Fig. 2(a), the white area treated with TTC was identified as infarcted tissue around most of the ipsilateral parts of the caudoputamen, neocortex, and thalamus, where blood is mainly supplied through the middle cerebral artery (MCA). This result means that our animal MCAO/R model was adequately prepared.

Next, we carried out immunofluorescence labeling with OX6 and HEt histochemistry to biochemically determine ROS generation in the activated microglia in the ischemic rat brain. OX6 is a monoclonal antibody that reacts to the MHC class II antigen expressed in activated microglia in the central nervous system (CNS)^24^. Microglia are resident macrophages in the brain and act as the first line of defense in the CNS. When the immune system is challenged and microglia are activated, the activated microglia have the potential to act in both a neurodegenerative and a neuroprotective fashion^10,25–27^. In a previous report, we demonstrated that activated microglia can actively produce and secrete ROS, and the well-controlled blockade of microglial activation can increase collateral neuronal survival^20^. In this study, we confirmed the existence of activated microglia within the infarcted regions by the immunofluorescence of OX6, as shown in Fig. 2(b). The microglial activation region closely coincides with the TTC stained area on the ipsilateral (right) side of the brain section. HEt is the most popular fluorogenic probe used for detecting ROS due to its easy acceptance by living cells. When it is oxidized in the presence of ROS, such as the intracellular superoxide radical anion (${\rm{O}}{\cdot }_{2}^{ \mbox{-} }$), it is converted into red fluorescent ethidium (ET), which in turn is trapped in cells through intercalation into DNA^28,29^. Figure 2(d,e) represent the FITC-conjugated OX6 and ET, respectively, in the same region. Figure 2(f) shows the merged image of Fig. 2(d,e), indicating the spatial coincidence between the activated microglia and ROS generation.

However, it should be noted that ET is not formed from the direct oxidation of HEt by ${\rm{O}}{\cdot }_{2}^{ \mbox{-} }$^30^. Instead, another product, 2-hydroxyethidium (2-OH-ET), is formed from the HEt/${\rm{O}}{\cdot }_{2}^{ \mbox{-} }$ reaction^31^, and it is known that 2-OH-ET is not formed during the reaction between HEt and other oxidants, such as ONOO^−^, ·OH, and H_2_O_2_. Therefore, HEt histochemistry may not be representative of the total ROS production in the brain parenchyma induced by ischemic damage. It should also be noted that ischemia causes the overproduction of ROS in the tissues and intracellular organelles, from which oxidative stress in normal cells (neurons) and tissues may occur. Therefore, ET-positive spots not coinciding with OX6-positive microglia in Fig. 2(f) (arrowheads) may be caused by other types of neuroglia and/or dying neurons.

Other than microglial cells, astrocytes are known to produce ROS in various pathological conditions in the CNS. Therefore, activated astrocytes could be labeled with ethidium histochemistry. However, and more importantly, the two types of neuronal cell death, necrosis and apoptosis, occur very extensively in the ischemic brain region (both in the ischemic core region and peri-infarct region). That is, ischemia causes the overproduction of ROS in intracellular organelles, and the consumption of endogenous antioxidants by these radicals may lead to a dramatic rise in intracellular ROS. Numerous studies have demonstrated that ROS can directly damage macromolecules (including lipids, proteins, and nucleic acids), leading to neuronal cell death. Therefore, a large amount of ROS can be produced and accumulated in dying neurons.

Following the biochemical confirmation of ROS generation, we obtained p-FMMD signal images of the magnetized OX6-conjugated SPIONs bound to the infarcted brain specimens. As shown in Fig. 2(c), the merged image of the p-FMMD signal with a brain section image indicates the apparent four reference points and the positive signal image. The p-FMMD signal image was imported into Adobe Photoshop, and only the positive signal was extracted by removing the background. Then, the image was adjusted and merged with the brain section image in accordance with the four reference points. As a result, the p-FMMD system could successfully identify the infarcted region in the brain slice by detecting the distribution of activated microglia. This result also indicates the spatial coincidence between the p-FMMD detection region and the biochemically determined region.

The degree of infarct size as determined by TTC staining, MRI, and p-FMMD was also investigated. Figure 2(a) shows the TTC staining image, Fig. 2(c) depicts the p-FMMD image, and Fig. 2(g) shows the T2 MR image at the same level of the rat brain after stroke, which had similar regions of infarction. The percent of infarct area compared to the contralateral counterpart was 60.9 ± 4.9% (mean ± SD) from TTC staining, 65.7 ± 2.7% rom MRI, and 44.6 ± 7.1% from p-FMMD. Quantitatively, the infarct ratio from T2 MRI is strongly correlated with the corresponding parameter from TTC staining, but the infarct ratio obtained from p-FMMD is significantly smaller than those obtained from MRI and TTC staining (Fig. 2(h)). The lower size of the ischemic volume analyzed by p-FMMD compared to that of TTC or MRI can be explained as follows. The p-FMMD signal representing microglia activation may only represent a portion of the infarct as measured by TTC and T2 MRI due to microglial activation not covering the full extent of the infarct area. Additionally, p-FMMD is a device that detects superparamagnetic substances. Therefore, if the SPIONs are inhomogeneously distributed in a specific region, relatively weak signals could be masked. This may explain the lower detection ratio from p-FMMD depicted in Fig. 2(h).

**Figure 3:**
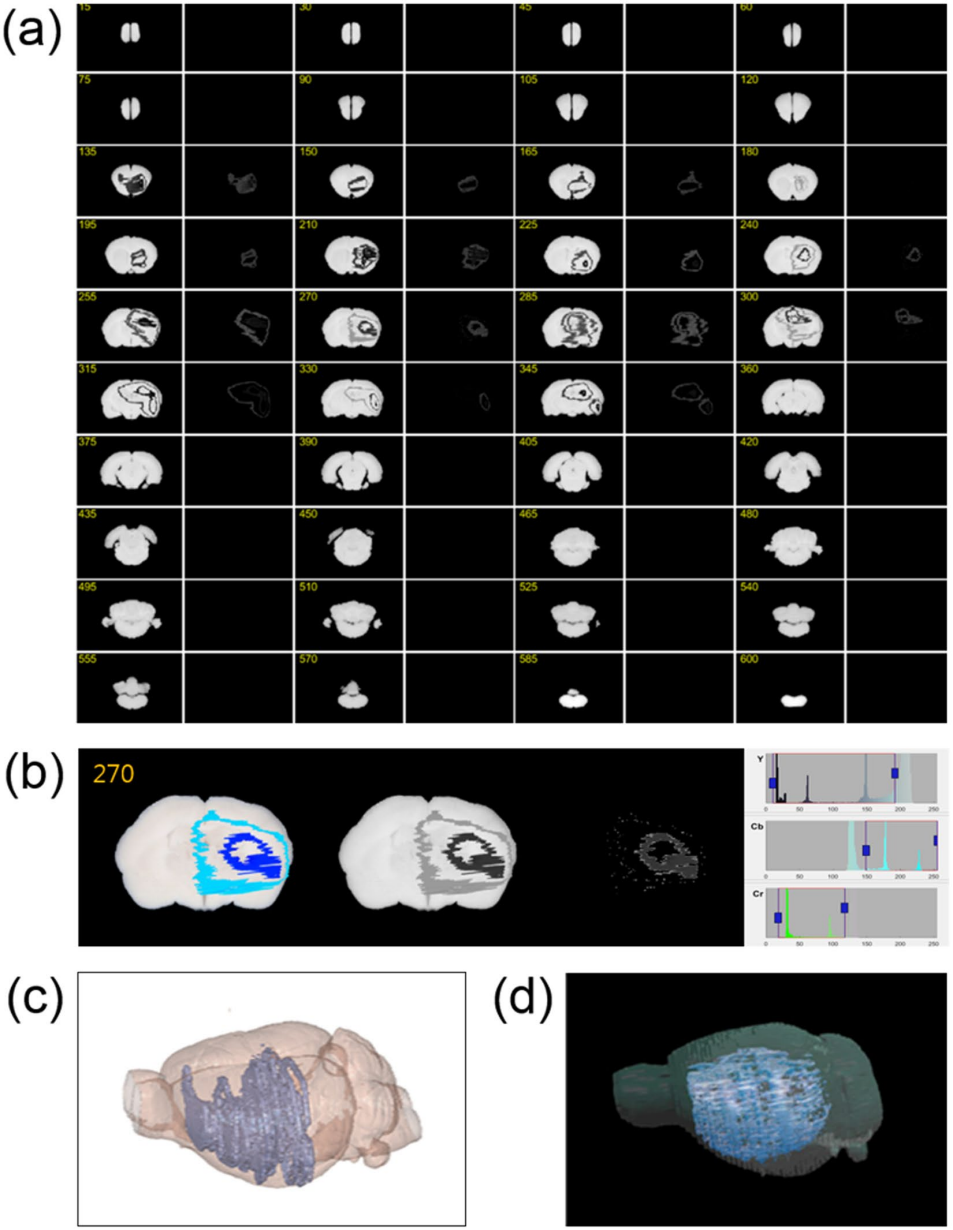
(**a**) 2D p-FMMD signal images obtained for every 15^th^ slice between slices 135 and 345 out of a total of 629 slices of the brain. (**b**) Segmentation results of the 270^th^ image setting YCbCr color spaces to adjust the threshold for brightness and color information of the p-FMMD magnetized signal image. (**c**) 3D rendered image by reconstructing the 2D stacked merged images of the brain sections and p-FMMD magnetized signal. (**d**) The infarction area was determined using MRI and visualized by 3D rendering. Threshold-based volume reconstruction of T2 hyperintensities are rendered as pulp in 3D objects. A decreased ratio of 3D spatial coincidence between the p-FMMD 3D reconstructed model (microglial activation in infarct region) and the 3D MRI model (infarct region) was found.

For the same reason, the signal images can be expanded to the periphery. As a result, the p-FMMD signals generated in the ischemic area (ipsilateral brain area) can spread and invade into the surrounding areas (contralateral brain region), as shown in Fig. 3(a). This explains why our p-FMMD system is not superior to conventional techniques (i.e., immunohistochemistry, immunofluorescence, TTC staining, MRI) for detecting the precise distribution of specific molecules in tissue specimens. However, we can demonstrate the applicability of our p-FMMD system as an *in situ* imaging tool for detecting superparamagnetic materials in biospecimens. As we noted in prior reports^12,16,21^, frequency mixing is known to be a useful method to isolate small quantities of weak signals of nonlinear materials from the linear bulk. Through this work, we successfully showed that our hypothesis is correct.

Microglia are highly plastic cells, and their activation is a complicated process that can be influenced by many substances and the surrounding microenvironments^26^. Resident microglia constantly patrol the CNS parenchyma and maintain homeostasis. However, once disturbed, they become activated and recruit neighboring microglia and/or peripheral macrophages to injury sites. Microglia dynamically interact with and are influenced by other cell types, such as neurons, astrocytes, oligodendrocytes, and endothelial cells, during different pathological stages^27^. Therefore, the microglial response is variable and changes over time poststroke. For example, Zhang *et al*. demonstrated time-dependent changes in the number of activated (OX6-positive) microglial cells in the infarct region after transient (2 hour) MCAO/R injury^32^. A significant increase in OX6-positive cells in the ischemic region was detected 10 hours after MCAO/R injury. The number of OX6-positive cells reached a maximum level at approximately 46 hours and persisted for up to 166 hours. Therefore, our p-FMMD infarct assessment using OX6-conjugated SPIONs can be changed according to the amount of time that has passed after infarction.

Next, we modeled the appearance of the whole brain by reconstructing the 2D stacked optical images of the 629 brain sections. We also performed segmentation of the model in accordance with the pixel coordinate system to reconstruct the volumetric shape of the magnetic signals of the OX6-conjugated SPIONs. Figure 3(a) represents the p-FMMD signal images of every 15th slice between slices 135 and 345 out of a total of 629 slices. Each brain section image was achromatically colored, and a thresholding process was performed based on the color information by adequately setting the YCbCr color space by adjusting the brightness and color information, as shown in Fig. 3(b). After the adjustment process, each segmentation image was resized and converted to a grayscale map for 3D reconstruction. Figure 3(c) shows the 3D-rendered model obtained by stacking every sliced brain optical image and segmented p-FMMD signal image. The transparency was adjusted to internally visualize the infarcted region within the whole brain. To compare the p-FMMD 3D reconstructed model with the MRI-based 3D model, a total of 53 images were taken 5 mm in thickness apart (see Supplement S7). Following MCAO/R, as expected, hyperintensity was typically observed in the ipsilateral cortex, caudoputamen, and hippocampal formations where blood is mainly supplied through the MCA. After converting every retained image to JPEG format, a 3D rendered image was generated using 3D-DOCTOR, as shown in Fig. 3(d), indicating a decreased ratio of coincidence of the infarcted regions compared with those of MRI models.

It is well known that various biological molecules, including antibodies, proteins, targeting ligands, etc., can be bound to the polymer surfaces of SPIONs without difficulty by chemical coupling via amide or ester bonds^33^. Currently, SPIONs offer high potential for many biomedical applications, including drug delivery, hyperthermia, magnetofection, nanotherapy, and so on^34^. In this study, we visualized the infarction region in real time by detecting activated microglia in an MCAO/R animal model using the p-FMMD technique. From an ischemic stroke biology point of view, our method might allow a different approach to analyze the pathophysiology of ischemic stroke through molecular imaging. Furthermore, we propose that this MPI technique detecting the nonlinear magnetization properties of SPIONs could be applied not only to stroke biology but also to various types of pathophysiological studies as a new molecular imaging tool. To consider that the p-FMMD system could have potential as a medical imaging system, we need to examine the current situation and the next requirements for practical use. Historically, studies of free radicals and ROS have been strictly confined to the fields of physics and chemistry. However, there is growing evidence that these fleeting molecules play critical roles in both normal physiology and pathophysiology. This recognition has led to the consensus that the development of noninvasive *in vivo* tools for detecting free radicals and/or ROS in living organisms, including the human body, could be a significant advancement in biomedical science. In this study, we demonstrated the feasibility of using our p-FMMD system as an *in situ* imaging tool for detecting superparamagnetic materials in biospecimens.

## Conclusion

In this study, we demonstrated the imaging of focal ischemia using a p-FMMD imaging system based on the detection of SPIONs in a biospecimen. The infarcted areas were confirmed using immunofluorescence labeling and MRI. The results of this study confirm that our p-FMMD method based on the detection of the nonlinear magnetic response of SPIONs constitutes a new technique for imaging biospecimens. This technique could potentially be applied to replace X-ray-based CT and radiation-based PET in the future.

## Supplementary information

Supplementary material

Dataset 1
