# Supplementary material for: Construction of 3D-rendering imaging of an ischemic rat brain model using the planar FMMD technique
Source: Sci Rep. 2019 Dec 13;9:19050. doi: 10.1038/s41598-019-55585-x (PMC6910971; doi:10.1038/s41598-019-55585-x)

Supplementary material

Construction of 3D-rendering imaging of an ischemic rat brain model using the planar FMMD technique

Chang-Beom Kim^a,†^, Sang-Jin Park^b,†^, Jae-Chan Jeong^a^, Seung-Min Choi^a^, Hans-Joachim Krause^c^,

Dae-Yong Song^b,^**, and Hyobong Hong^a,^*

^a^ SW Contents Research Lab., Electronics and Telecommunications Research Institute (ETRI), 218 Gajeong-Ro, Yuseong-Gu, Daejeon 34129, Republic of Korea

^b^ Department of Anatomy and Neuroscience, School of Medicine, Eulji University, 77 Gyeryong-Ro, Jung-Gu, Daejeon 34824, Republic of Korea

^c^ Institute of Complex Systems, Bioelectronics (ICS-8), Forschungszentrum Jülich, Jülich 52425, Germany

^†^ These authors equally contributed to this work.

* Corresponding Author 1

*Mailing address*: SW Contents Research Lab., Electronics and Telecommunications Research Institute (ETRI), 218 Gajeong-Ro, Yuseong-Gu, Daejeon 34129, Republic of Korea

*Phone No*.: +82-42-860-6663

*E. mail*: hb8868@etri.re.kr

** Corresponding Author 2

*Mailing address*: Department of Anatomy and Neuroscience, Eulji University School of Medicine, 77 Gyeryong-Ro, Jung-Gu, Daejeon 34824, Republic of Korea

*Phone No*.: +82-42-259-1622

*E. mail*: dysong@eulji.ac.kr

**S1. Surgical procedure**

The surgical procedure was carried out as previously described^17^. Rats were anesthetized with ketamine (70 mg/kg body weight) and xylazine (8 mg/kg body weight) intraperitoneally. After midline incision of the neck, the left common carotid artery (CCA) was exposed and carefully dissected free from the surrounding nerves and fascia. The external carotid artery (ECA) was isolated by identification of the branching superior thyroid artery and then coagulated. The internal carotid artery (ICA) was also isolated and carefully separated from the adjacent vagus nerve. A punctate incision was made in the ventral wall of the CCA with a 25G needle, and a MCAO suture (403965PK10, Doccol Corporation, Sharon, MA, USA) was introduced into the ICA lumen and then advanced approximately 25 mm when resistance was felt and a slight curving of the suture was observed. The incised skin was clipped, leaving 1 cm of the MCAO suture protruding so it could be withdrawn to allow reperfusion. Sixty minutes after MCAO, the MCAO suture was removed, and the incised skin was completely sutured. During the operation, the experimental animals were maintained at 37 °C with a heat lamp and a heating pad. The rats were allowed to survive for 1 day and then sacrificed.

**S2. Brain sectioning**

Rats were anesthetized and transcardially perfused with physiological saline, followed by 400 mL of 4 % paraformaldehyde in phosphate-buffered saline (PBS). The brains were removed immediately, postfixed in the same fixative for 2 hours, and infiltrated with 30 % sucrose solution for 24 hours at 4 °C until they sank. The whole brains were rapidly frozen in 2-methylbutane chilled on dry ice and mounted in Tissue-Tek OCT compound (Sakura Finetechnical Co., Tokyo, Japan). Serial coronal sections 40 μm thick were obtained with a cryostat microtome (Leica Microsystems Inc., Wetzlar, Germany).

**S3. OX6 conjugation with SPIONs (Carbodiimide method)**

Superparamagnetic iron oxide nanoparticles (SPIONs) were conjugated with OX6 antibody using the manufacturer’s instructions (Chemicell GmbH, Berlin, Germany). Briefly, 10 mg of fluid MAG-amine SPIONs (size 100 nm, number of particles 1.8 × 10^15^/g) were washed with 1 ml of MES buffer (0.1 M 2-(N-morpholino)ethanesulfonic acid, pH 5.0) twice using a magnetic separator. The SPIONs were suspended in 0.25 ml of MES buffer containing 10 mg of EDC (1-ethyl-3-[3-dimethylaminopropyl]carbodiimide hydrochloride, Pierce, Rockford) and mixed on a shaker for 10 min at room temperature. After two washes, 50 mg/ml OX6 antibody was added, and the mixture was incubated at room temperature on a shaker for 2 hours. The OX6-conjugated SPIONs (1 mg/ml final concentration) were harvested using a magnetic separator and resuspended in 50 μl of PBS containing 0.05 % sodium azide for preservation. The OX6-conjugated SPIONs were washed twice with PBS buffer prior to use.

**S4. Validation of the specificity and reliability of the p-FMMD system on the biospecimens**

The experimental animal was anesthetized and secured in a stereotaxic apparatus (Stoelting Co., Wood Dale, IL, USA). The skull was exposed by a midline incision, and a small hole was made with a dental drill on the right side of the skull 2.0 mm posterior to the bregma and 3.0 mm lateral (right) to the midline. A 26 gauge steel needle attached to a 10 µl Hamilton syringe (Hamilton Co., Reno, NV, USA) was lowered through the hole to a depth of 5.0 mm from the skull. The 10 µl of paramagnetic beads used in this study as SPIONs were administered using a microinfusion pump (KD Scientific Inc., Holliston, MA, USA) at a rate of 2 µl/min. The needle was left in place for an additional 5 min before slowly retracting it from the brain. On the left side of the brain (coordinates: 0.8 mm anterior to the bregma, 3.0 mm lateral (left) to the midline, and 6.0 mm depth), 10 µl of hematoxylin was administered as a control (A). After one hour of survival, the animal was sacrificed. Following cardiac perfusion, the brain was extracted, and 400 µm of coronal serial sectioning was performed using a vibratome. The p-FMMD scan results showed signal only in the brain area injected with paramagnetic particles (black arrows in B) and not in the brain region injected with hematoxylin used as a negative control (white arrowheads in B). The 3D reconstruction is represented in (C).


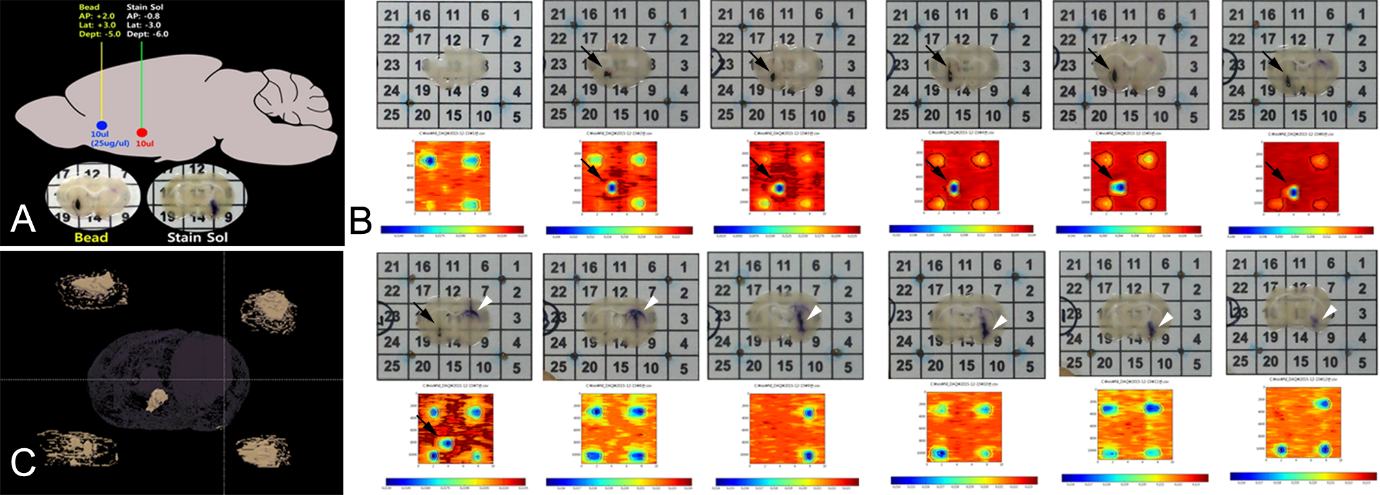


**S5. MATLAB source code for 3D rendering**


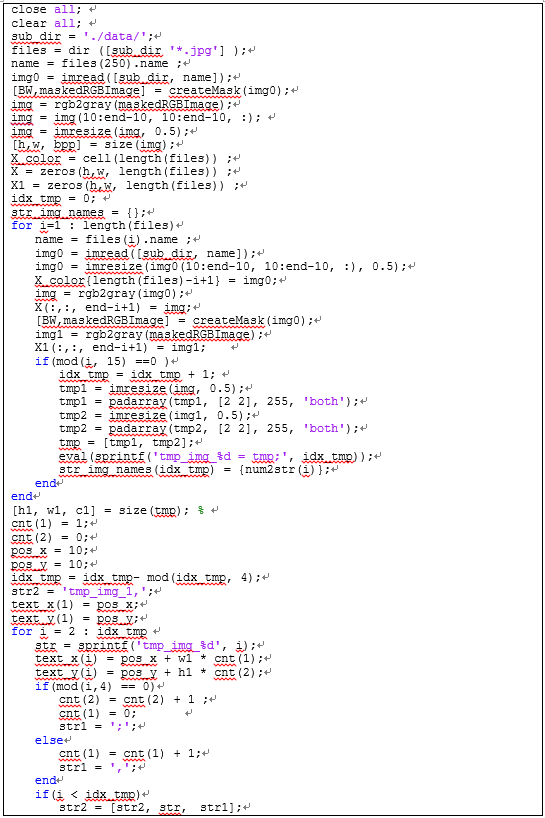


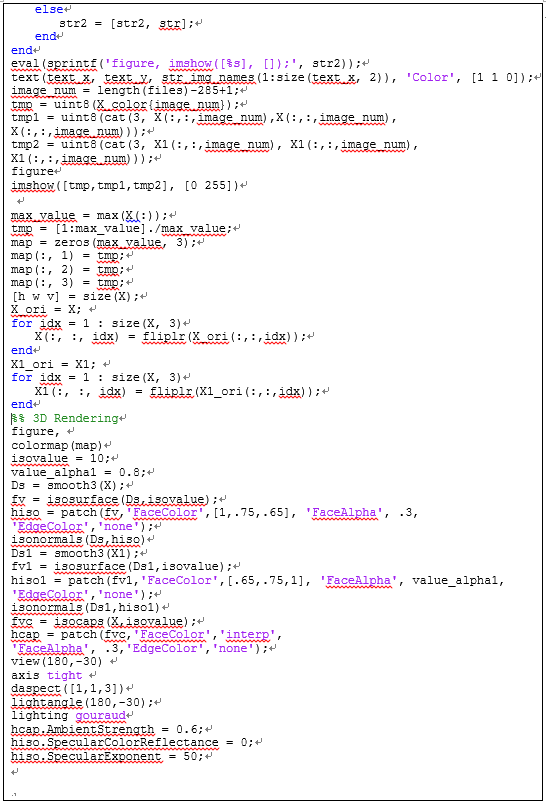


**S6.** Representative T1-4 repeat, T1-8 repeat, T2-4 repeat, and T2-8 repeat MR images of the rat brain. The T1- and T2-weighted images can be easily differentiated by examining the cerebrospinal fluid (CSF). The CSF is dark on the T1-weighted imaging and bright on the T2-weighted imaging. Ischemic damage usually breaks down the blood-brain barrier or the blood-CSF barrier, allowing fluid to accumulate in the brain parenchyma. When comparing the four images, the T2-8 repeat MR image is the clearest and shows the distribution of brain fluid accurately.

**
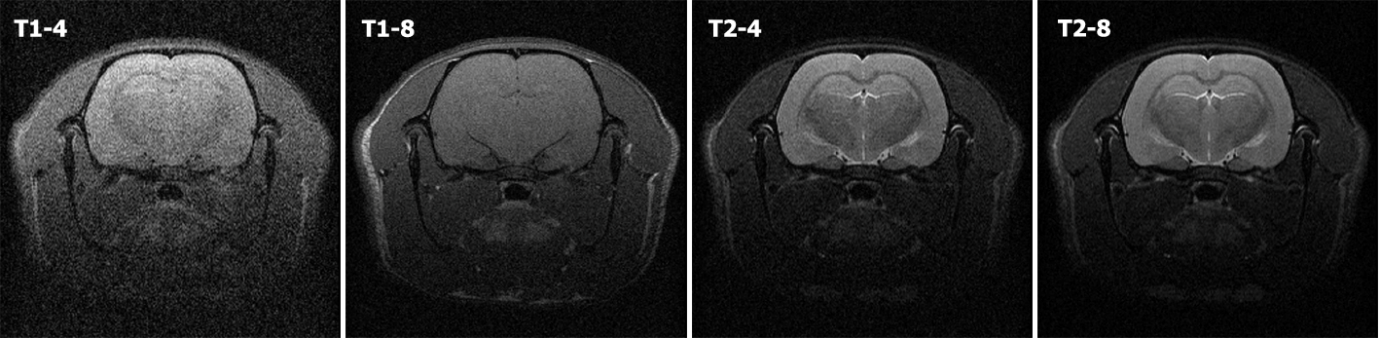
**

**S7.** Representative T2 MRI images after MCAO and reperfusion injury to a rat. T2 hyperintensities appeared in the ischemic brain regions in the serial coronal MRI images.


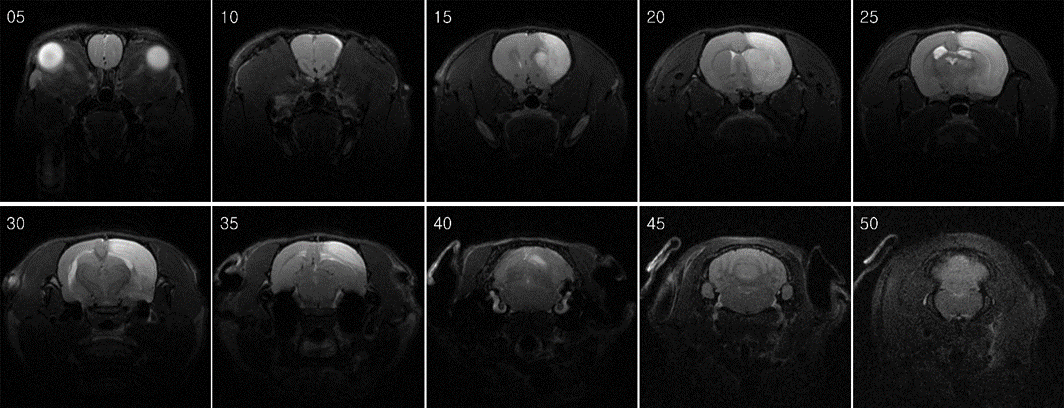

Supplement: Supplementary file 1 — Supplementary material [file 41598_2019_55585_MOESM1_ESM.docx]
