# Supplementary figures and images for: Construction of 3D-rendering imaging of an ischemic rat brain model using the planar FMMD technique (part 2 of 2)
Source: Sci Rep. 2019 Dec 13;9:19050. doi: 10.1038/s41598-019-55585-x (PMC6910971; doi:10.1038/s41598-019-55585-x)

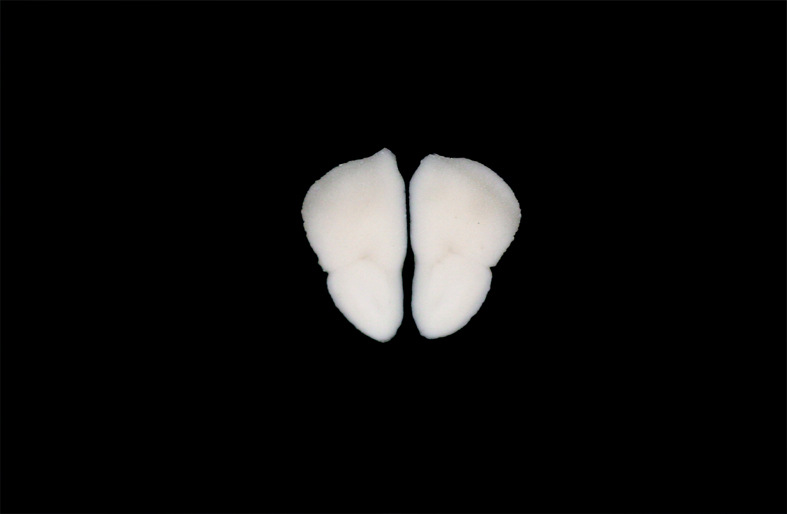

Supplement: Supplementary file 2 — Dataset 1 [file 41598_2019_55585_MOESM2_ESM.zip › 101.jpg]

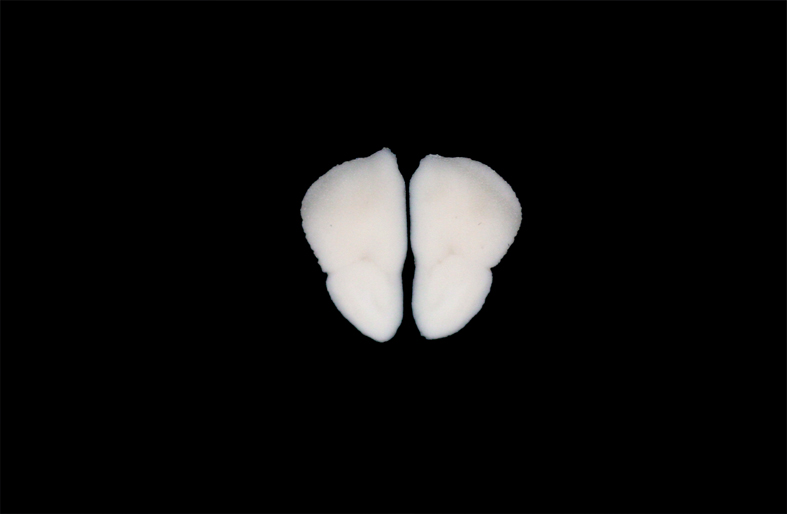

Supplement: Supplementary file 2 — Dataset 1 [file 41598_2019_55585_MOESM2_ESM.zip › 102.jpg]

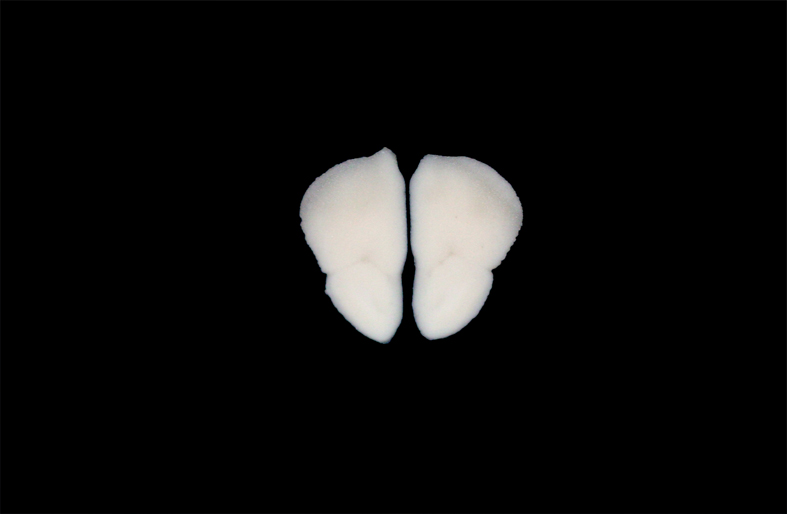

Supplement: Supplementary file 2 — Dataset 1 [file 41598_2019_55585_MOESM2_ESM.zip › 103.jpg]

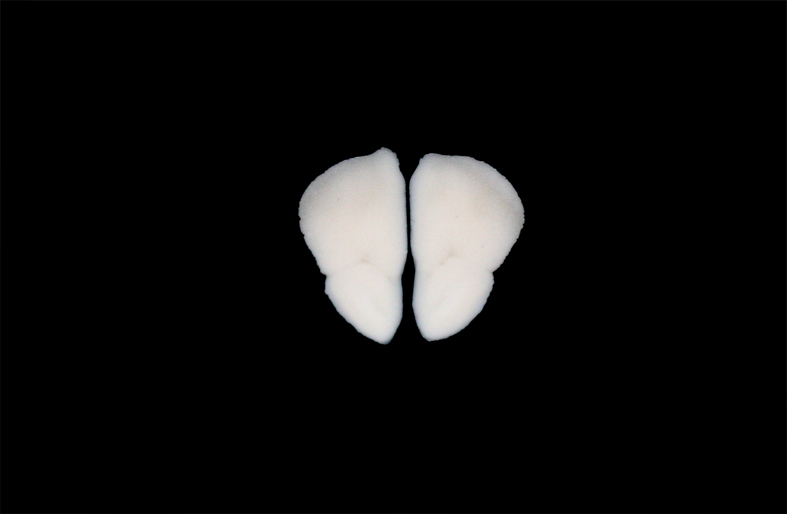

Supplement: Supplementary file 2 — Dataset 1 [file 41598_2019_55585_MOESM2_ESM.zip › 104.jpg]

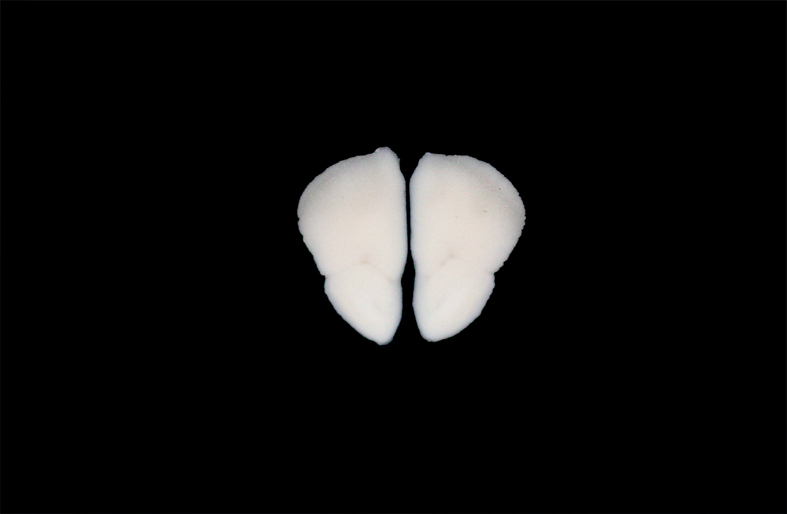

Supplement: Supplementary file 2 — Dataset 1 [file 41598_2019_55585_MOESM2_ESM.zip › 105.jpg]

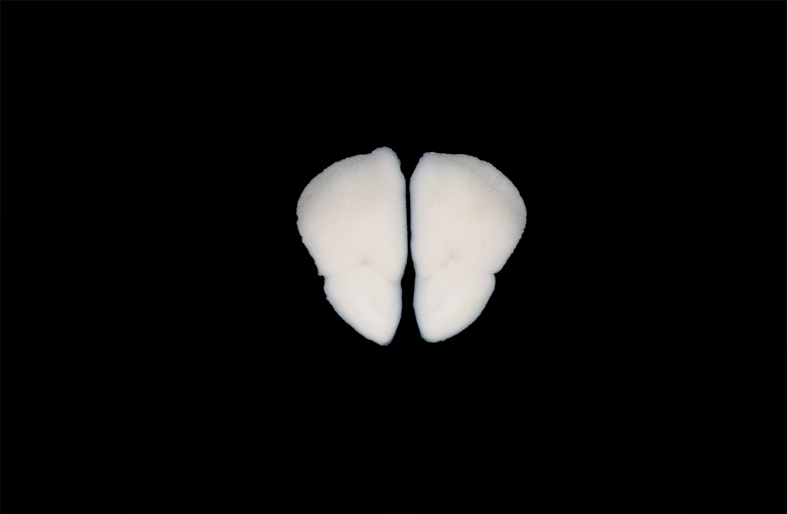

Supplement: Supplementary file 2 — Dataset 1 [file 41598_2019_55585_MOESM2_ESM.zip › 106.jpg]

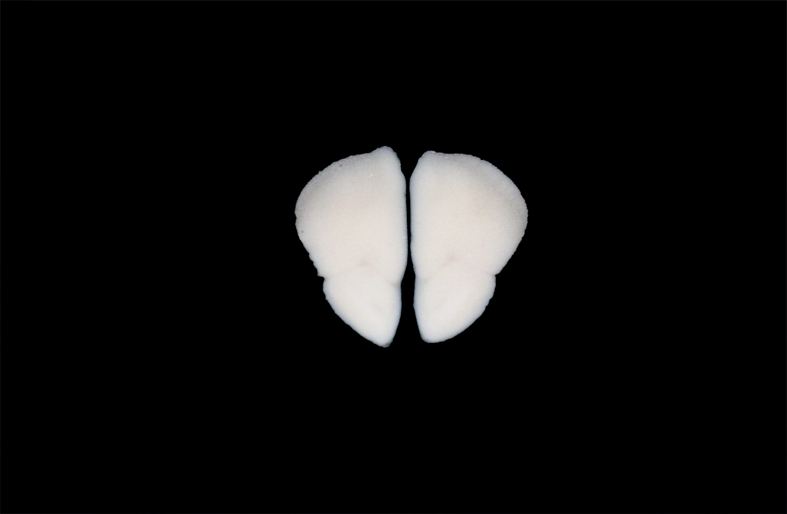

Supplement: Supplementary file 2 — Dataset 1 [file 41598_2019_55585_MOESM2_ESM.zip › 107.jpg]

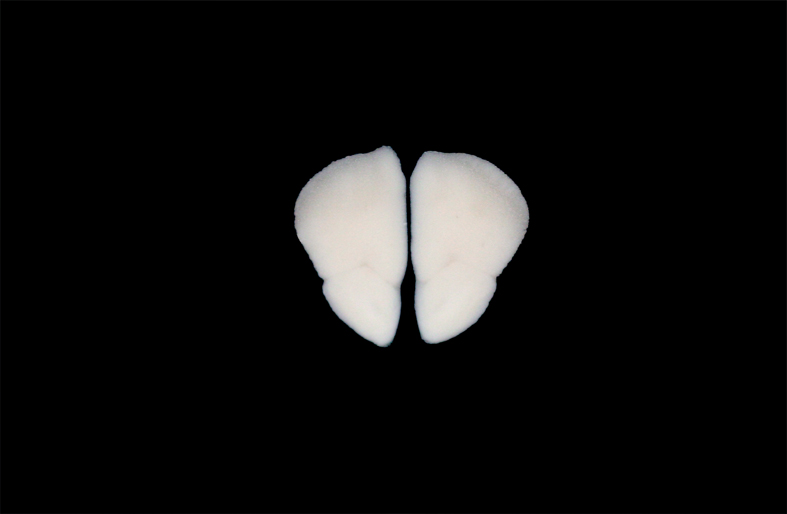

Supplement: Supplementary file 2 — Dataset 1 [file 41598_2019_55585_MOESM2_ESM.zip › 108.jpg]

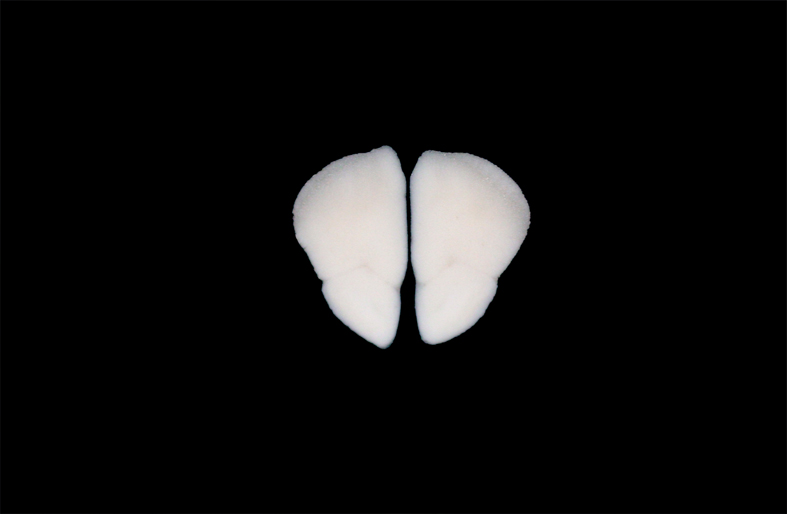

Supplement: Supplementary file 2 — Dataset 1 [file 41598_2019_55585_MOESM2_ESM.zip › 109.jpg]

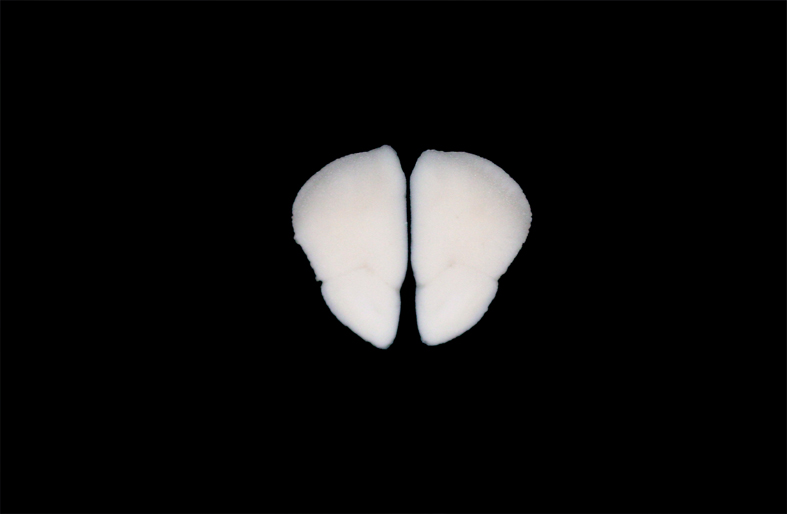

Supplement: Supplementary file 2 — Dataset 1 [file 41598_2019_55585_MOESM2_ESM.zip › 110.jpg]

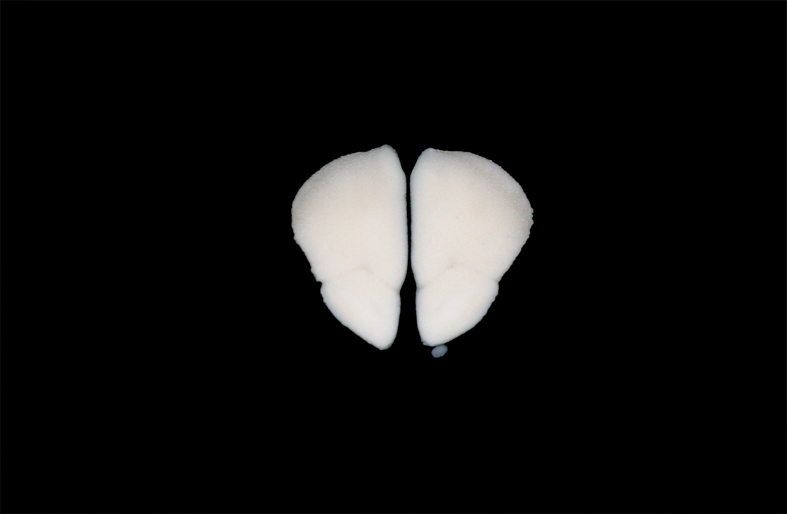

Supplement: Supplementary file 2 — Dataset 1 [file 41598_2019_55585_MOESM2_ESM.zip › 111.jpg]

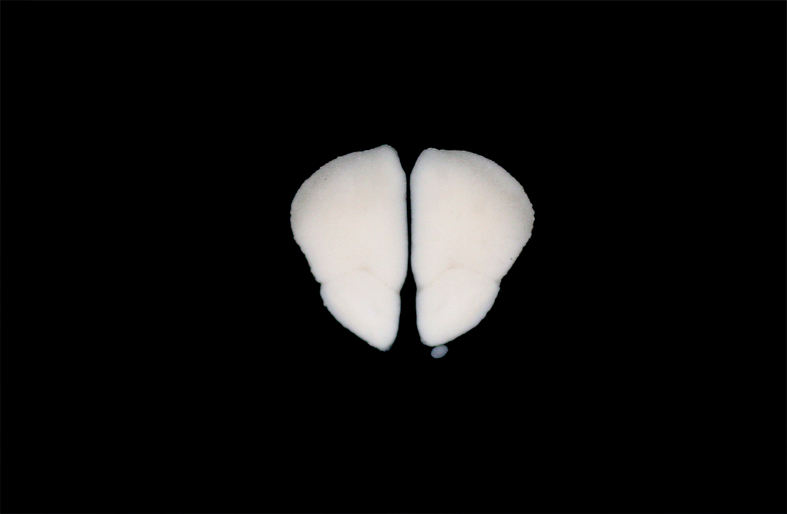

Supplement: Supplementary file 2 — Dataset 1 [file 41598_2019_55585_MOESM2_ESM.zip › 112.jpg]

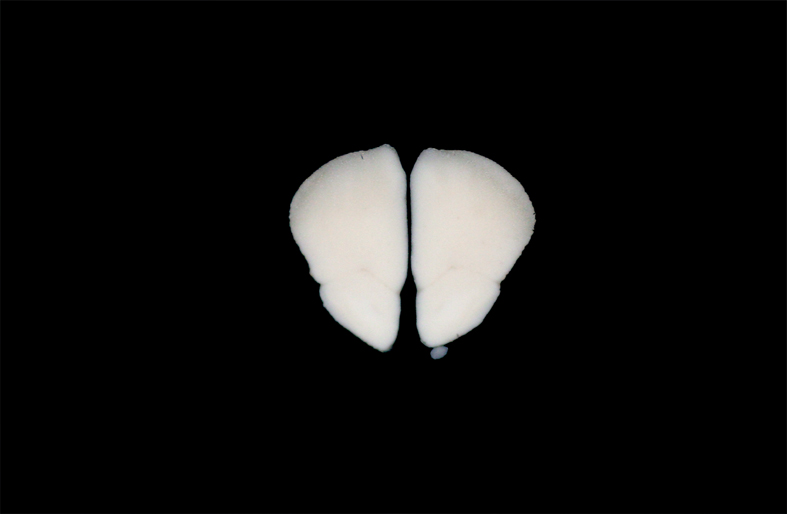

Supplement: Supplementary file 2 — Dataset 1 [file 41598_2019_55585_MOESM2_ESM.zip › 113.jpg]

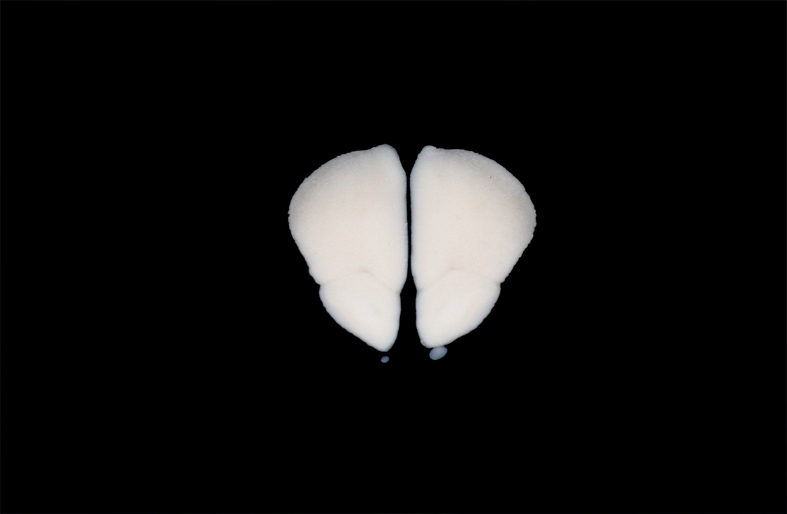

Supplement: Supplementary file 2 — Dataset 1 [file 41598_2019_55585_MOESM2_ESM.zip › 114.jpg]

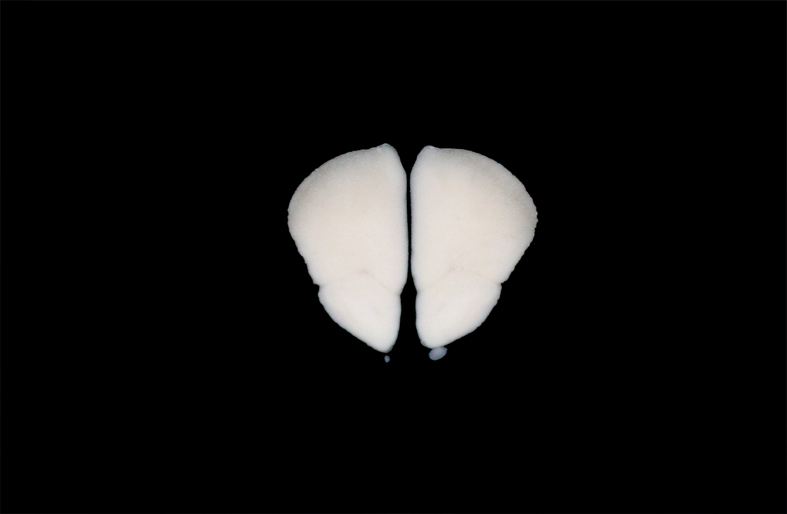

Supplement: Supplementary file 2 — Dataset 1 [file 41598_2019_55585_MOESM2_ESM.zip › 115.jpg]

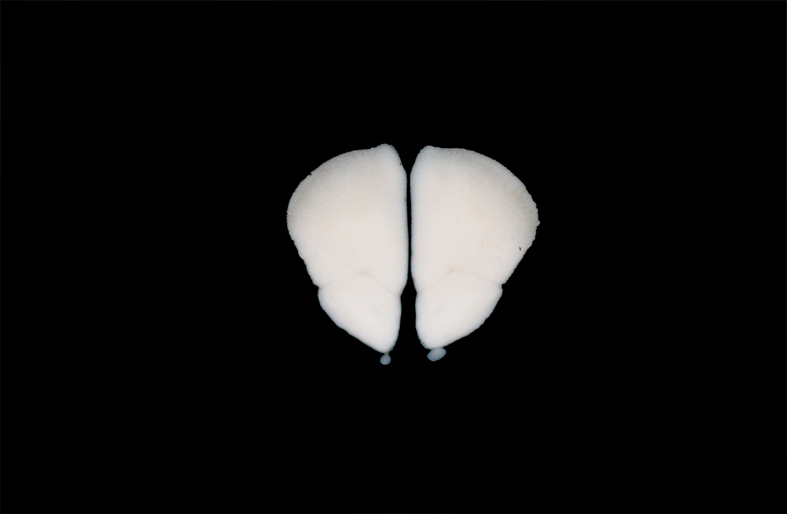

Supplement: Supplementary file 2 — Dataset 1 [file 41598_2019_55585_MOESM2_ESM.zip › 116.jpg]

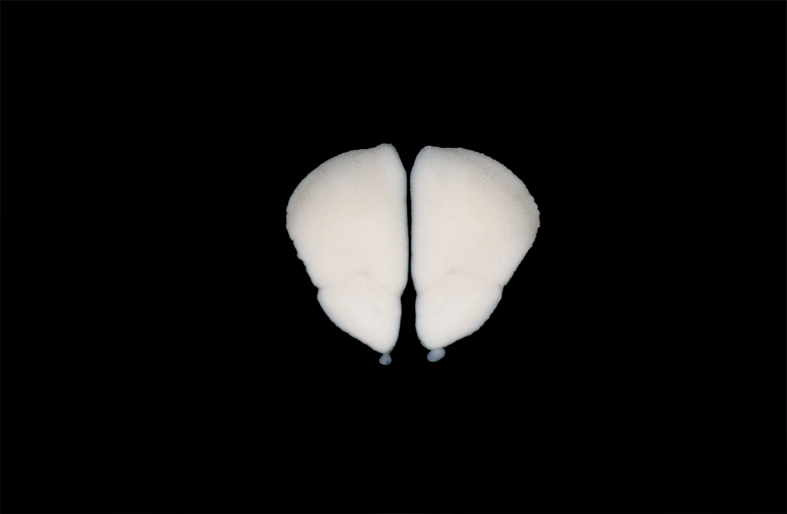

Supplement: Supplementary file 2 — Dataset 1 [file 41598_2019_55585_MOESM2_ESM.zip › 117.jpg]

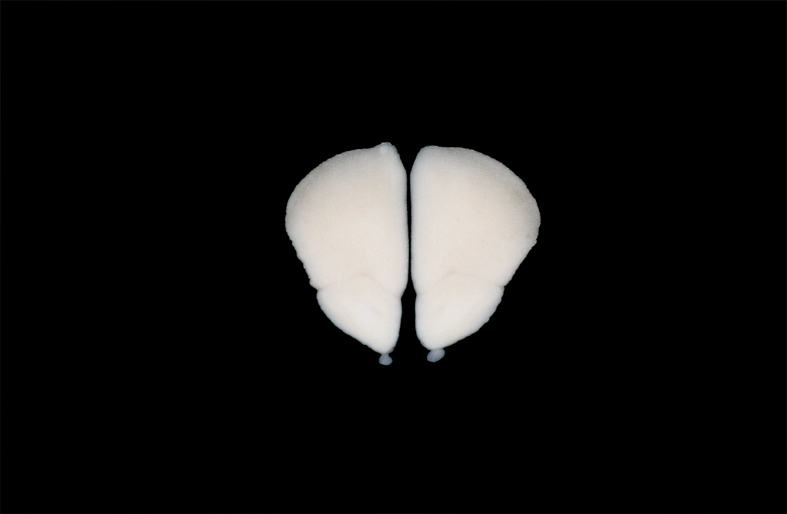

Supplement: Supplementary file 2 — Dataset 1 [file 41598_2019_55585_MOESM2_ESM.zip › 118.jpg]

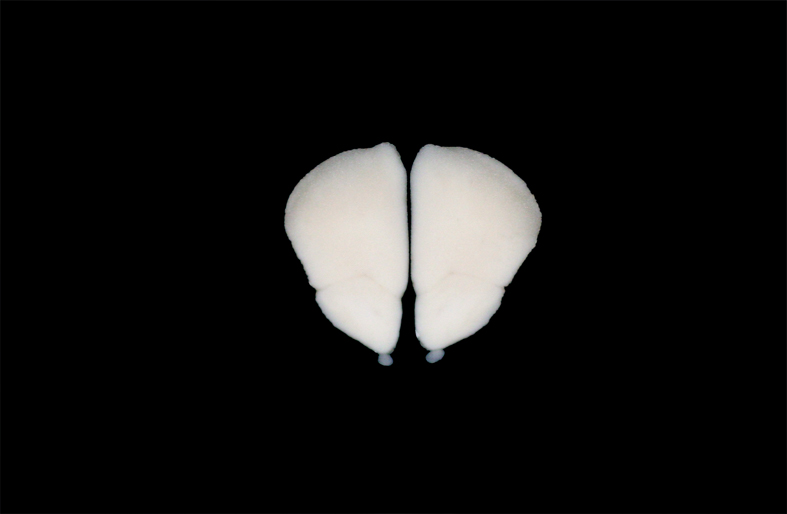

Supplement: Supplementary file 2 — Dataset 1 [file 41598_2019_55585_MOESM2_ESM.zip › 119.jpg]

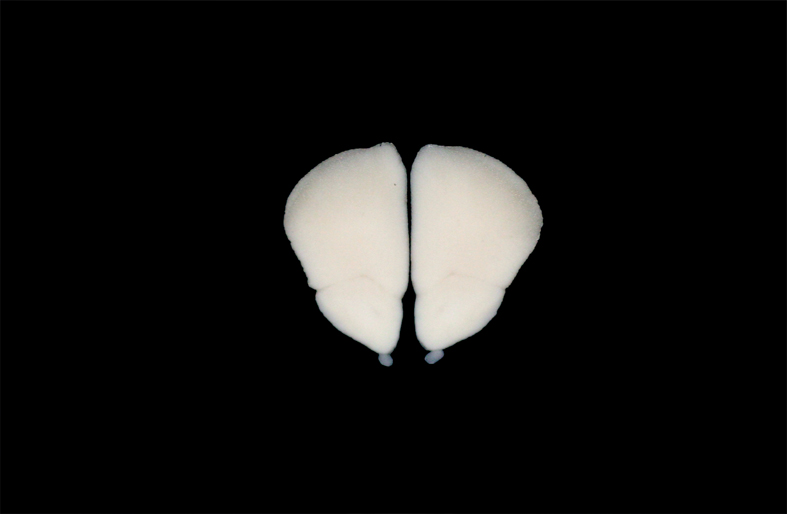

Supplement: Supplementary file 2 — Dataset 1 [file 41598_2019_55585_MOESM2_ESM.zip › 120.jpg]

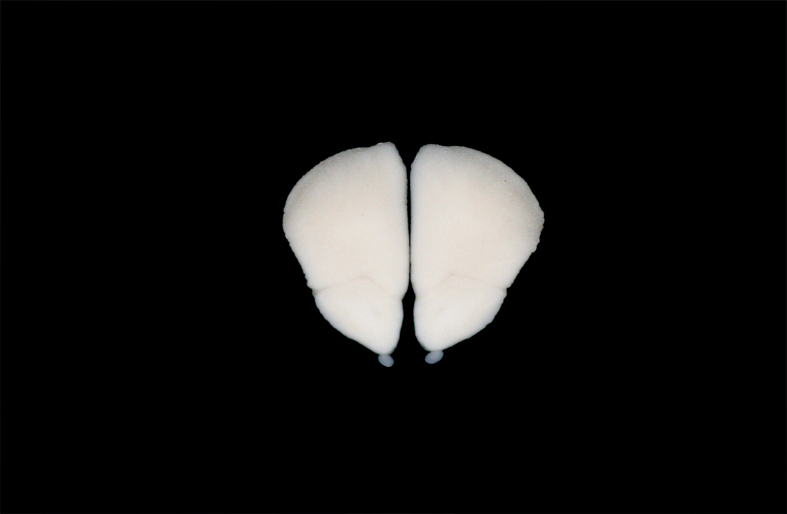

Supplement: Supplementary file 2 — Dataset 1 [file 41598_2019_55585_MOESM2_ESM.zip › 121.jpg]

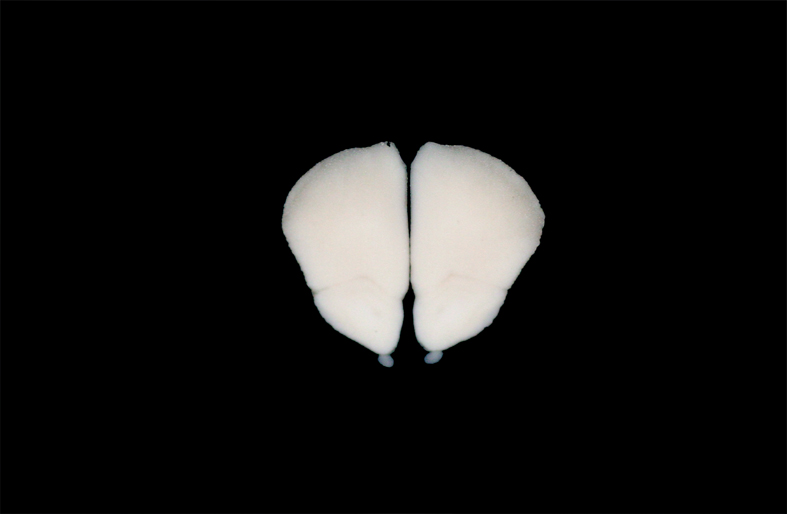

Supplement: Supplementary file 2 — Dataset 1 [file 41598_2019_55585_MOESM2_ESM.zip › 122.jpg]

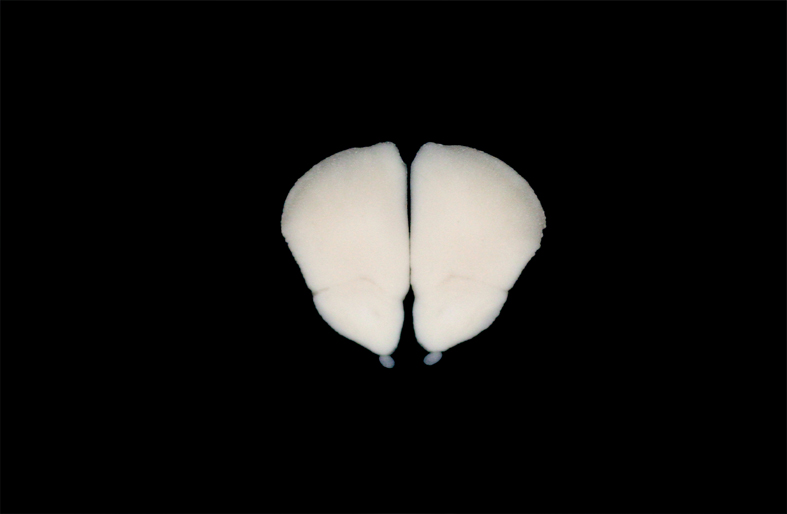

Supplement: Supplementary file 2 — Dataset 1 [file 41598_2019_55585_MOESM2_ESM.zip › 123.jpg]

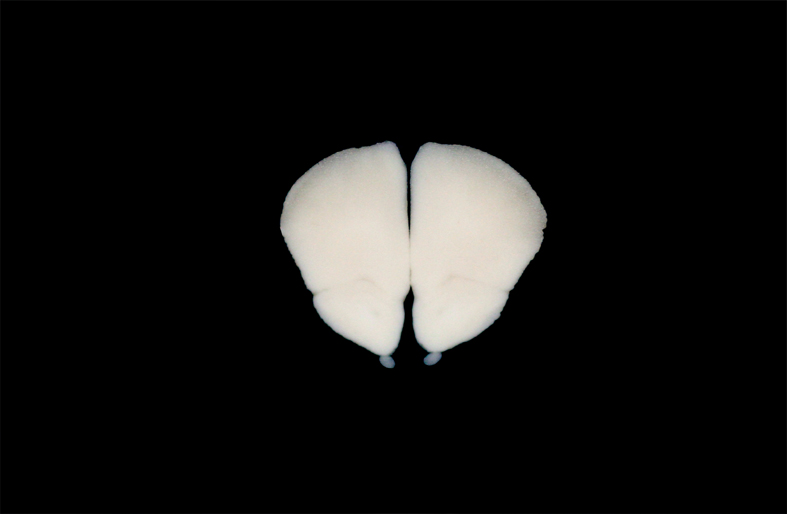

Supplement: Supplementary file 2 — Dataset 1 [file 41598_2019_55585_MOESM2_ESM.zip › 124.jpg]

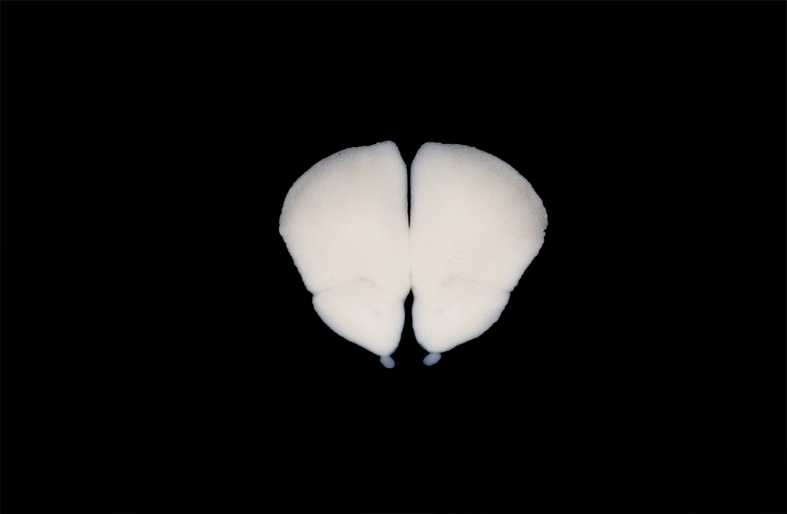

Supplement: Supplementary file 2 — Dataset 1 [file 41598_2019_55585_MOESM2_ESM.zip › 125.jpg]

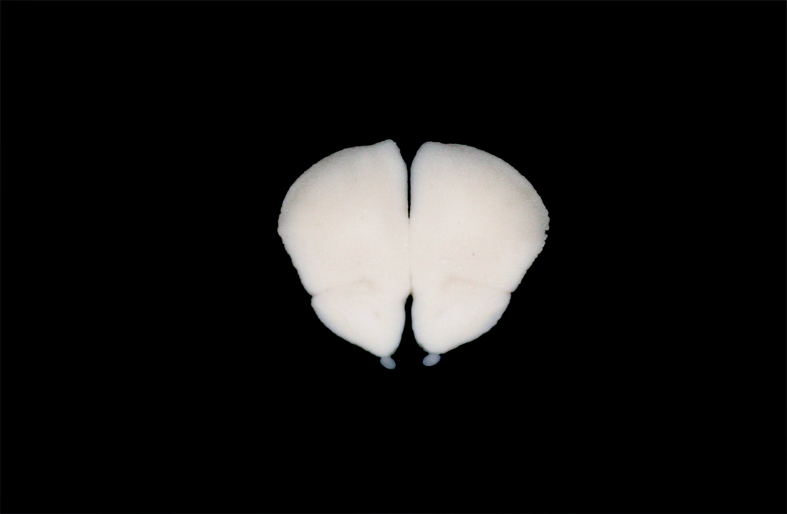

Supplement: Supplementary file 2 — Dataset 1 [file 41598_2019_55585_MOESM2_ESM.zip › 126.jpg]

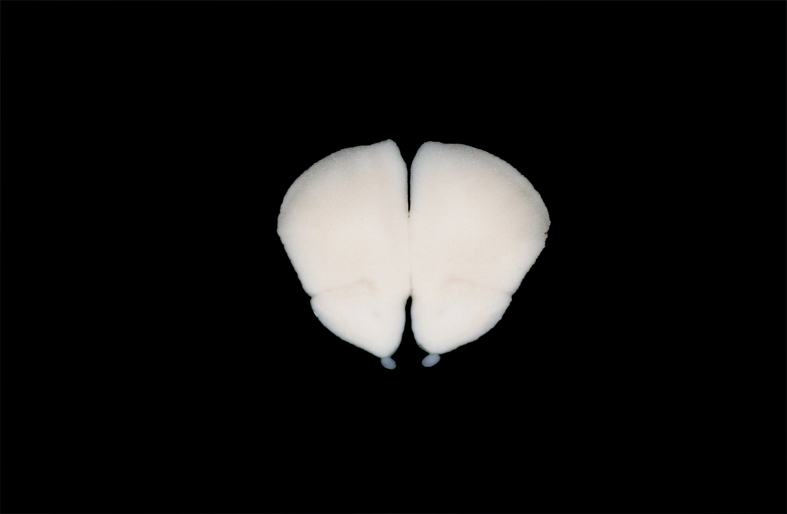

Supplement: Supplementary file 2 — Dataset 1 [file 41598_2019_55585_MOESM2_ESM.zip › 127.jpg]

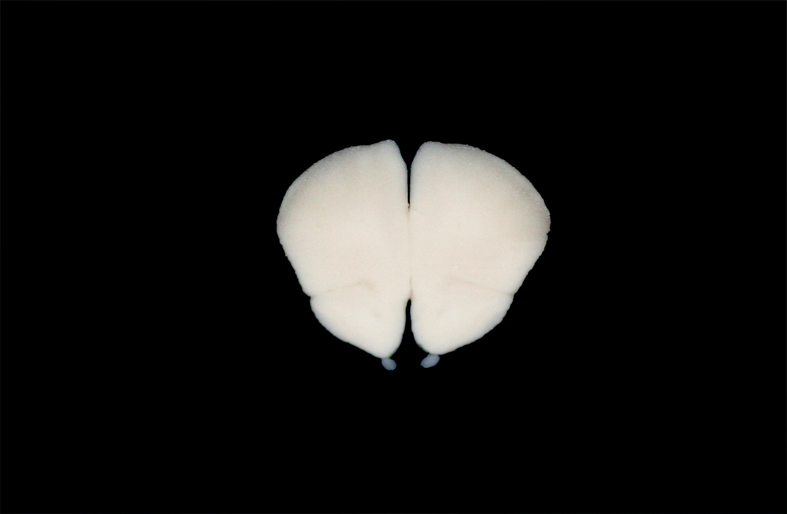

Supplement: Supplementary file 2 — Dataset 1 [file 41598_2019_55585_MOESM2_ESM.zip › 128.jpg]

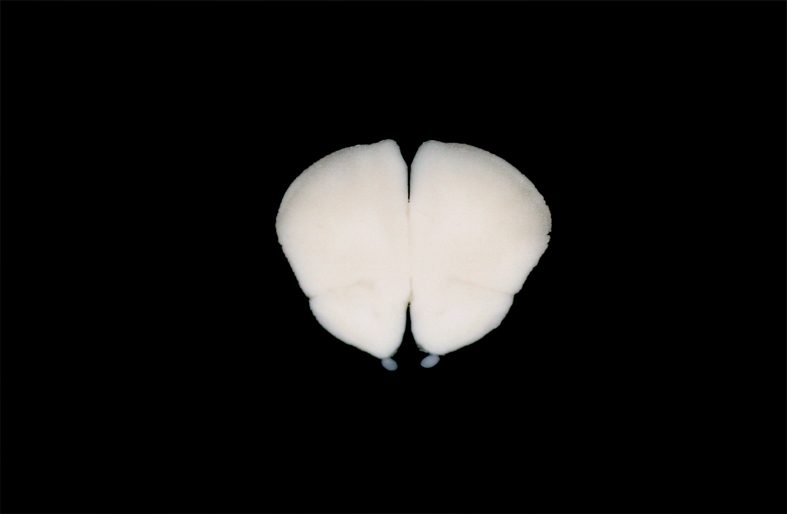

Supplement: Supplementary file 2 — Dataset 1 [file 41598_2019_55585_MOESM2_ESM.zip › 129.jpg]

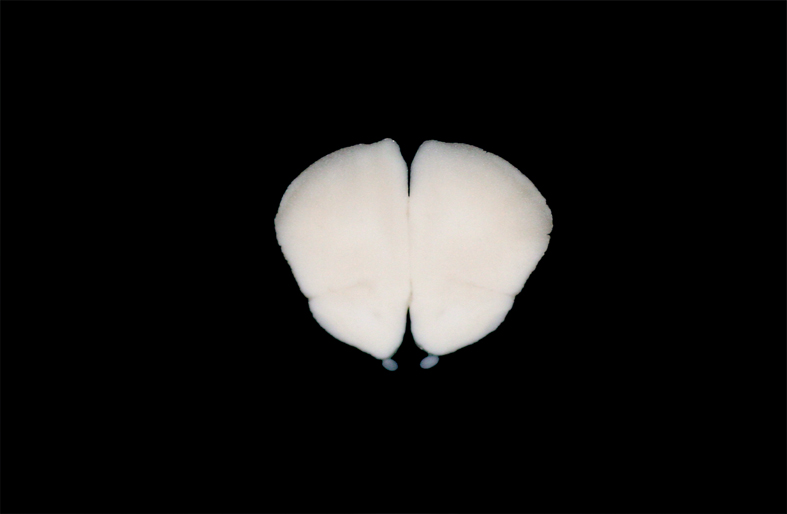

Supplement: Supplementary file 2 — Dataset 1 [file 41598_2019_55585_MOESM2_ESM.zip › 130.jpg]

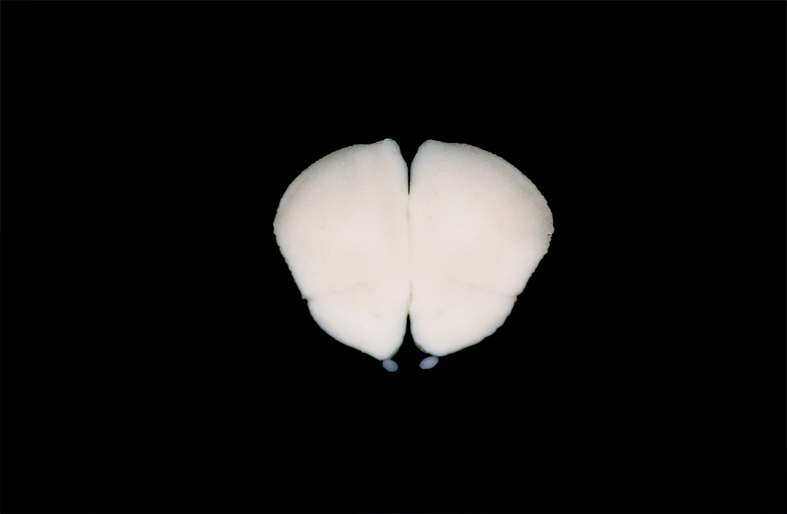

Supplement: Supplementary file 2 — Dataset 1 [file 41598_2019_55585_MOESM2_ESM.zip › 131.jpg]

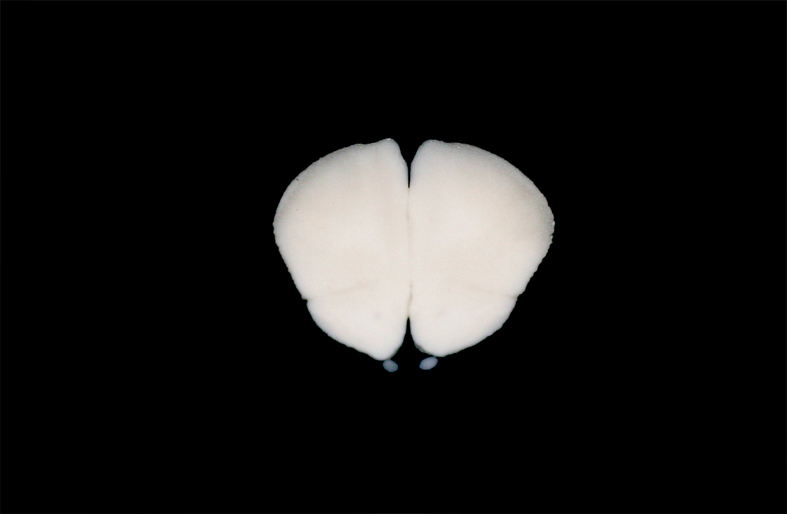

Supplement: Supplementary file 2 — Dataset 1 [file 41598_2019_55585_MOESM2_ESM.zip › 132.jpg]

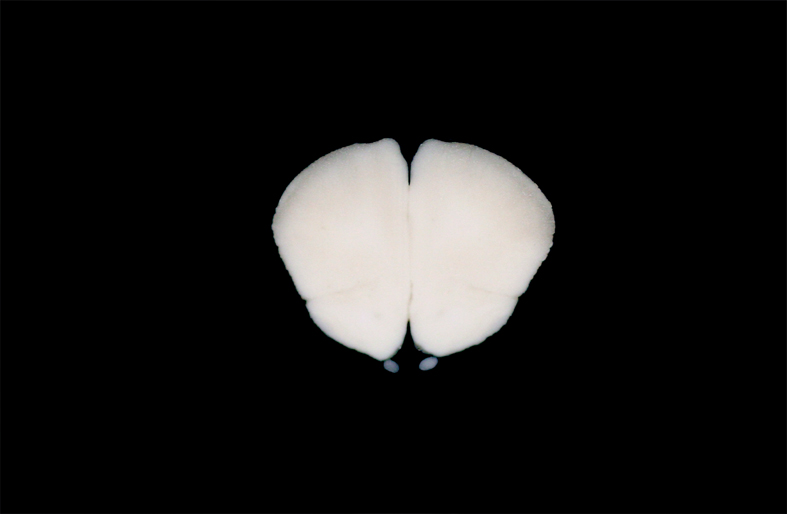

Supplement: Supplementary file 2 — Dataset 1 [file 41598_2019_55585_MOESM2_ESM.zip › 133.jpg]

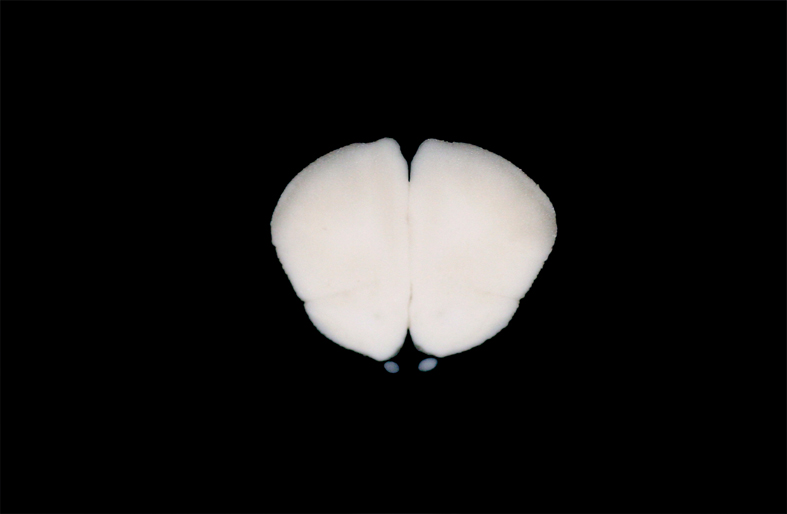

Supplement: Supplementary file 2 — Dataset 1 [file 41598_2019_55585_MOESM2_ESM.zip › 134.jpg]

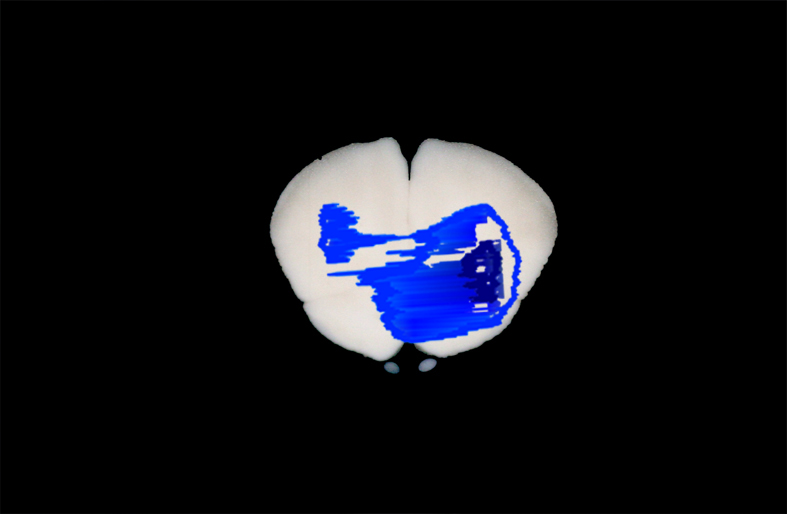

Supplement: Supplementary file 2 — Dataset 1 [file 41598_2019_55585_MOESM2_ESM.zip › 135.jpg]

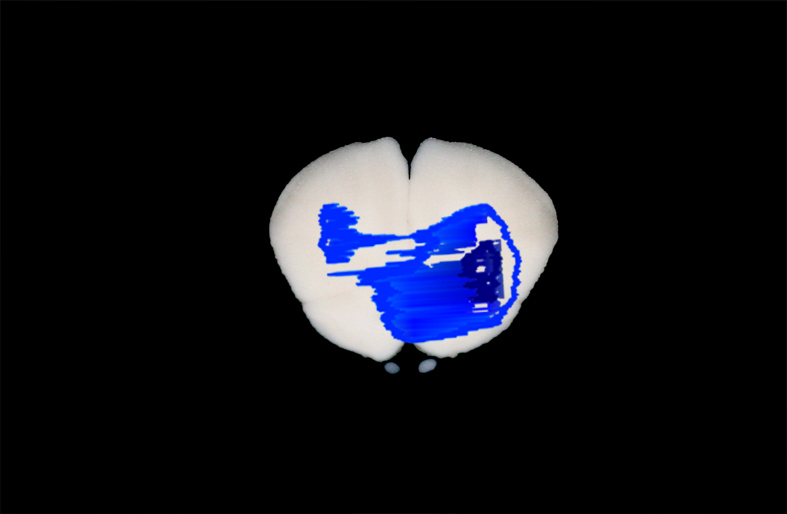

Supplement: Supplementary file 2 — Dataset 1 [file 41598_2019_55585_MOESM2_ESM.zip › 136.jpg]

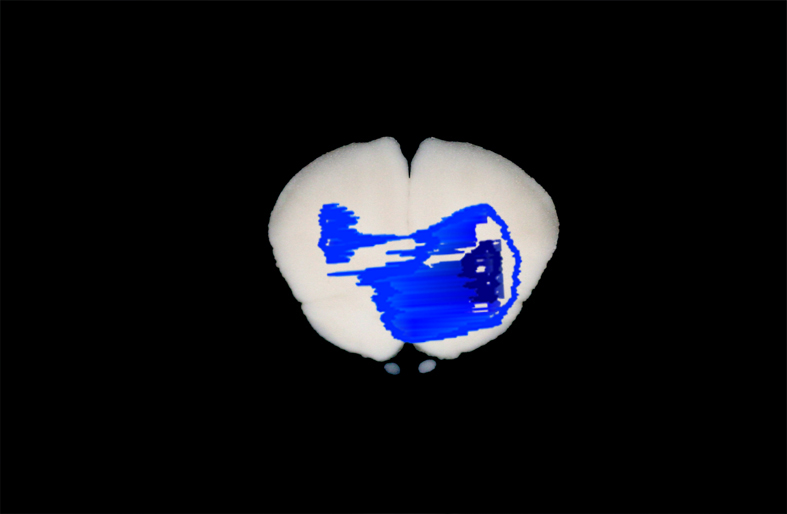

Supplement: Supplementary file 2 — Dataset 1 [file 41598_2019_55585_MOESM2_ESM.zip › 137.jpg]

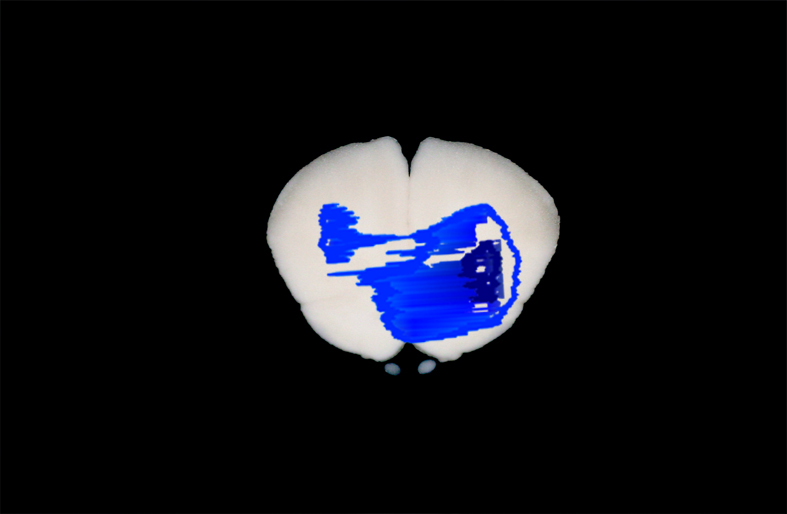

Supplement: Supplementary file 2 — Dataset 1 [file 41598_2019_55585_MOESM2_ESM.zip › 138.jpg]

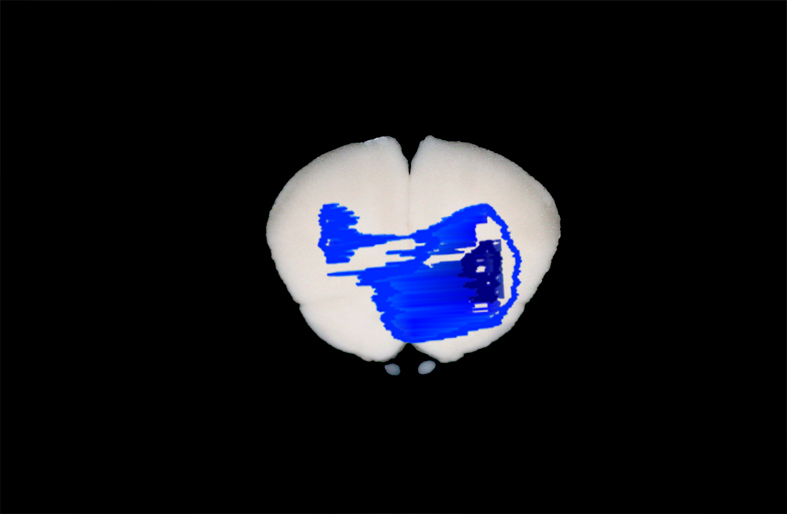

Supplement: Supplementary file 2 — Dataset 1 [file 41598_2019_55585_MOESM2_ESM.zip › 139.jpg]

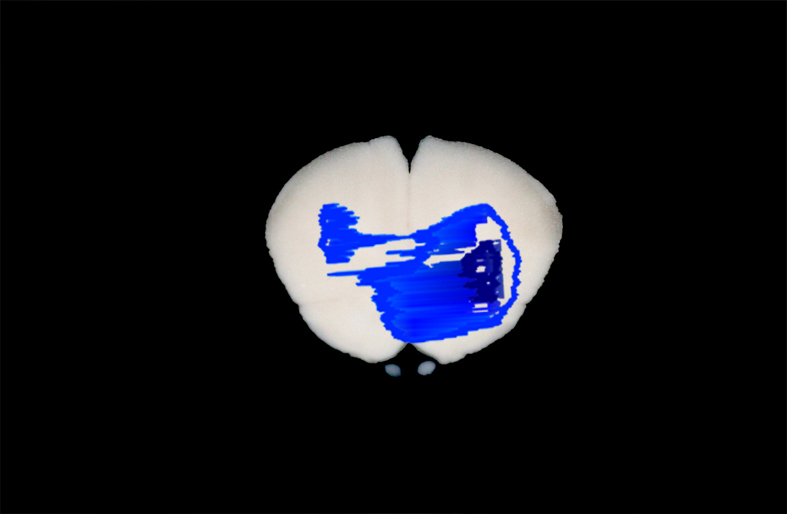

Supplement: Supplementary file 2 — Dataset 1 [file 41598_2019_55585_MOESM2_ESM.zip › 140.jpg]

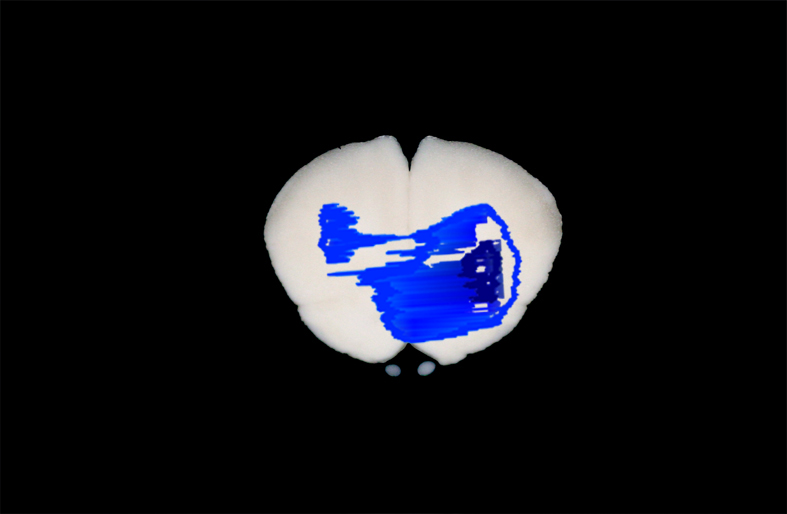

Supplement: Supplementary file 2 — Dataset 1 [file 41598_2019_55585_MOESM2_ESM.zip › 141.jpg]

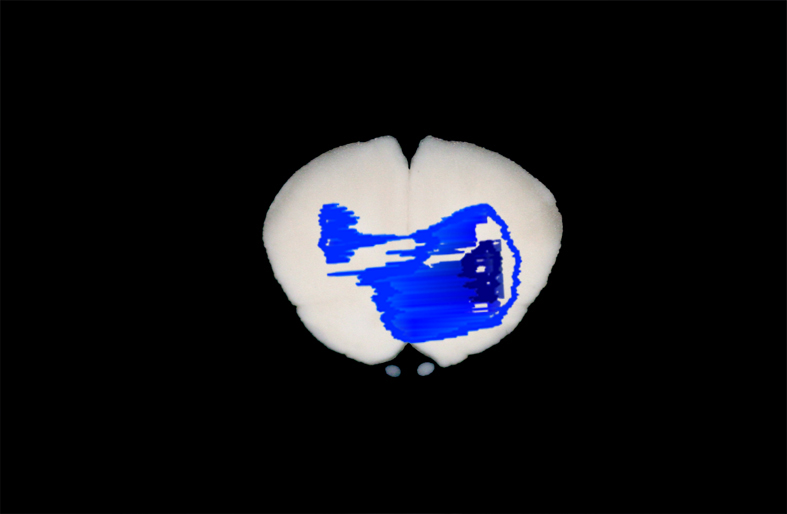

Supplement: Supplementary file 2 — Dataset 1 [file 41598_2019_55585_MOESM2_ESM.zip › 142.jpg]

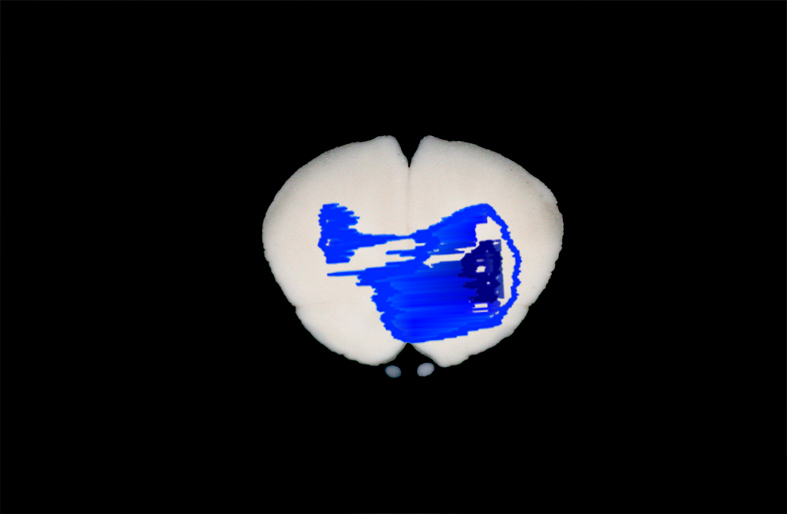

Supplement: Supplementary file 2 — Dataset 1 [file 41598_2019_55585_MOESM2_ESM.zip › 143.jpg]

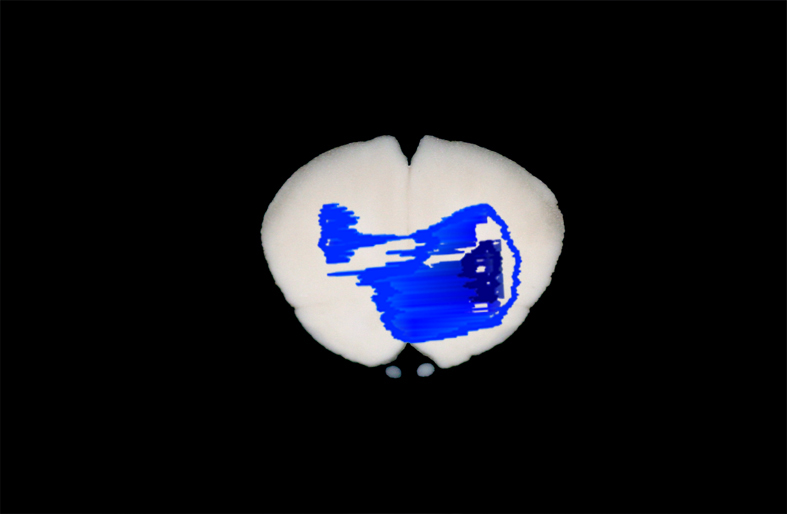

Supplement: Supplementary file 2 — Dataset 1 [file 41598_2019_55585_MOESM2_ESM.zip › 144.jpg]

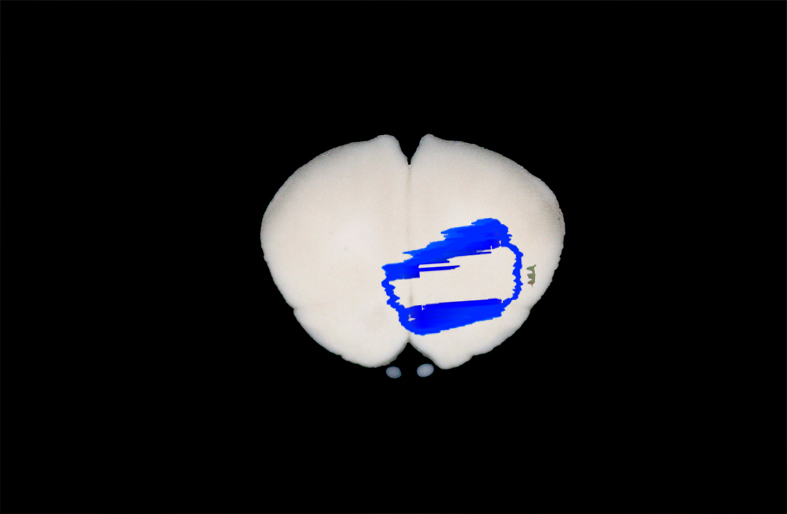

Supplement: Supplementary file 2 — Dataset 1 [file 41598_2019_55585_MOESM2_ESM.zip › 145.jpg]

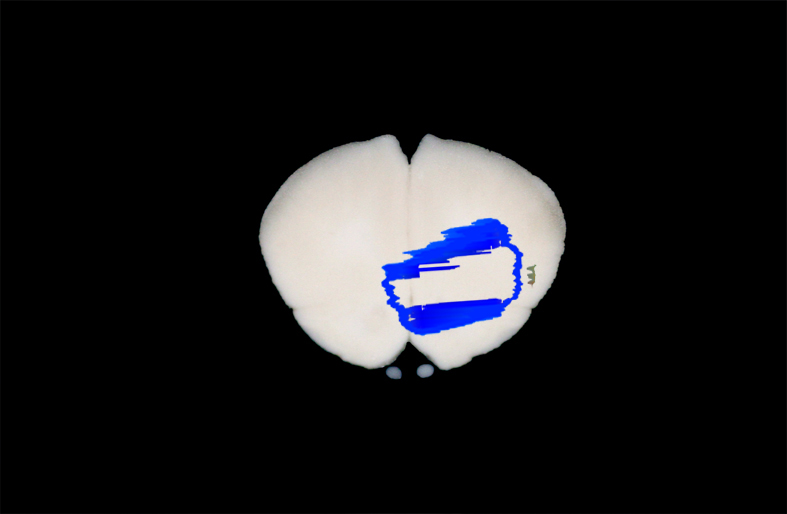

Supplement: Supplementary file 2 — Dataset 1 [file 41598_2019_55585_MOESM2_ESM.zip › 146.jpg]

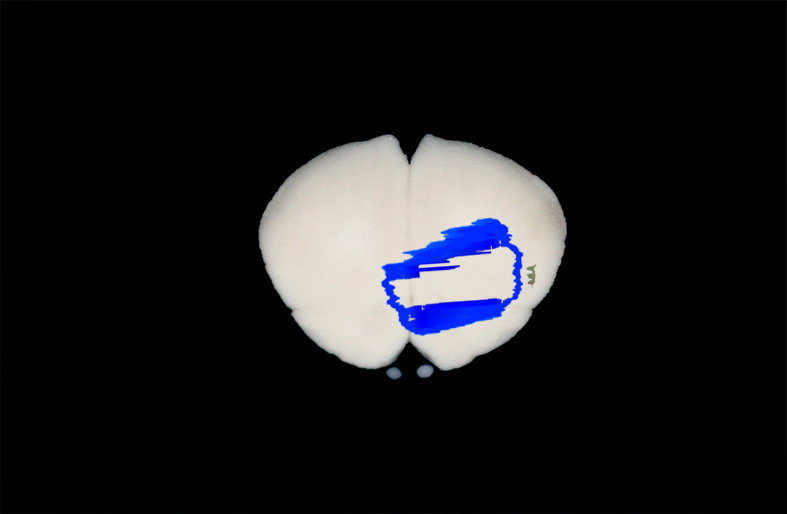

Supplement: Supplementary file 2 — Dataset 1 [file 41598_2019_55585_MOESM2_ESM.zip › 147.jpg]

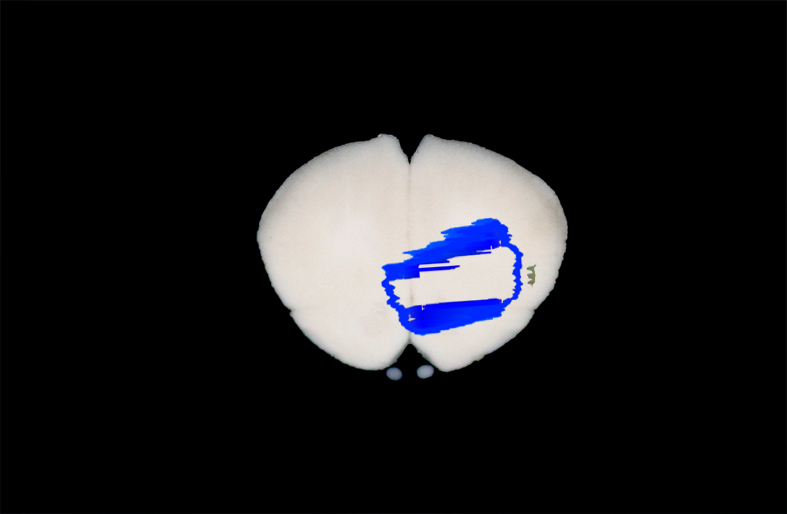

Supplement: Supplementary file 2 — Dataset 1 [file 41598_2019_55585_MOESM2_ESM.zip › 148.jpg]

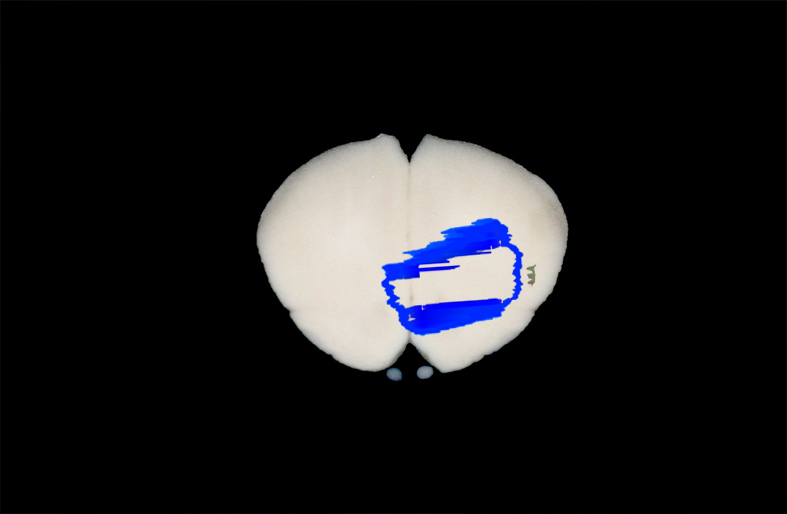

Supplement: Supplementary file 2 — Dataset 1 [file 41598_2019_55585_MOESM2_ESM.zip › 149.jpg]

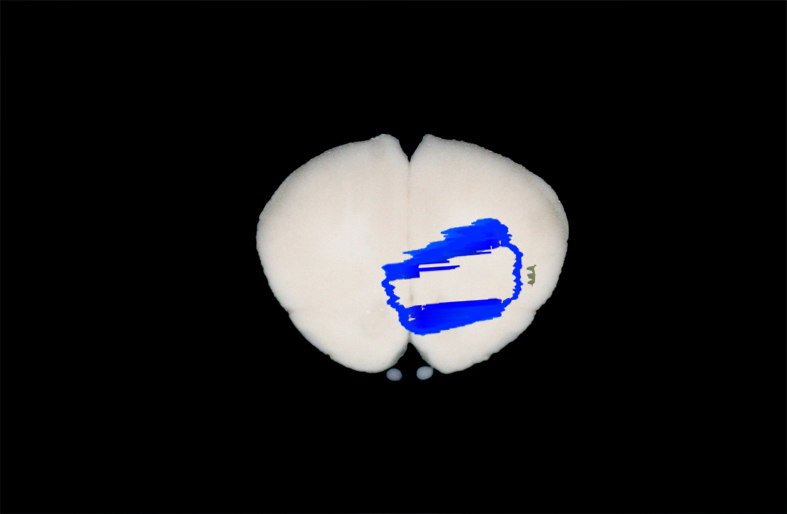

Supplement: Supplementary file 2 — Dataset 1 [file 41598_2019_55585_MOESM2_ESM.zip › 150.jpg]

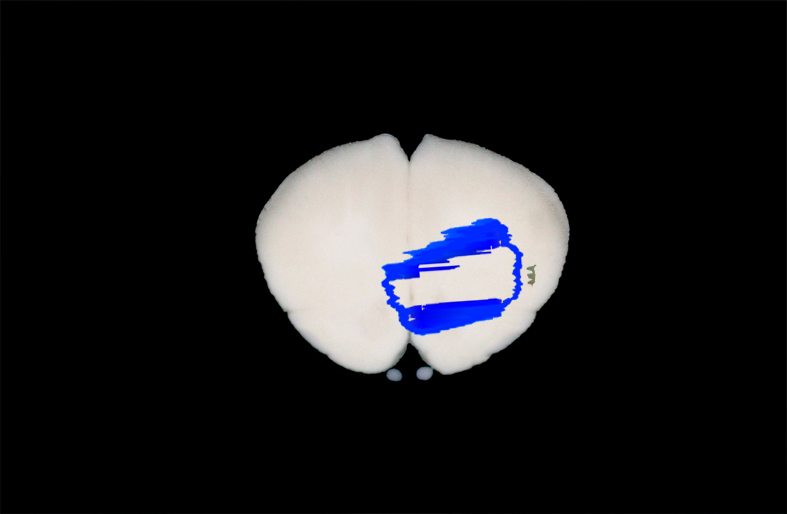

Supplement: Supplementary file 2 — Dataset 1 [file 41598_2019_55585_MOESM2_ESM.zip › 151.jpg]

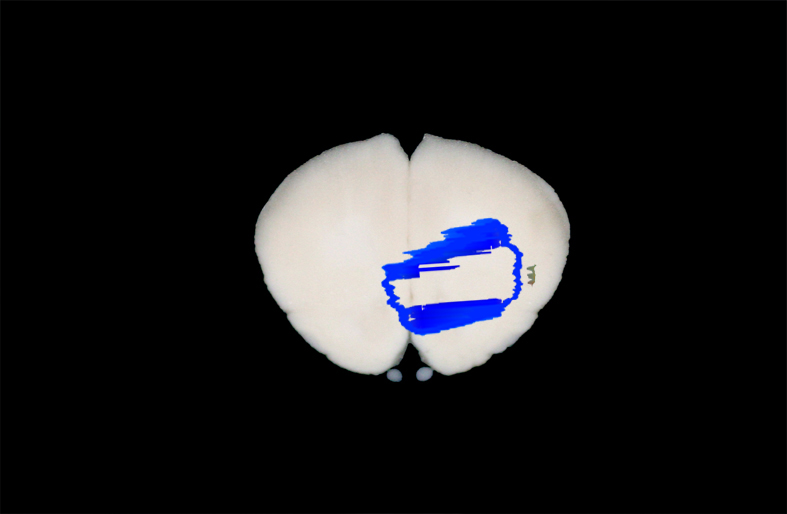

Supplement: Supplementary file 2 — Dataset 1 [file 41598_2019_55585_MOESM2_ESM.zip › 152.jpg]

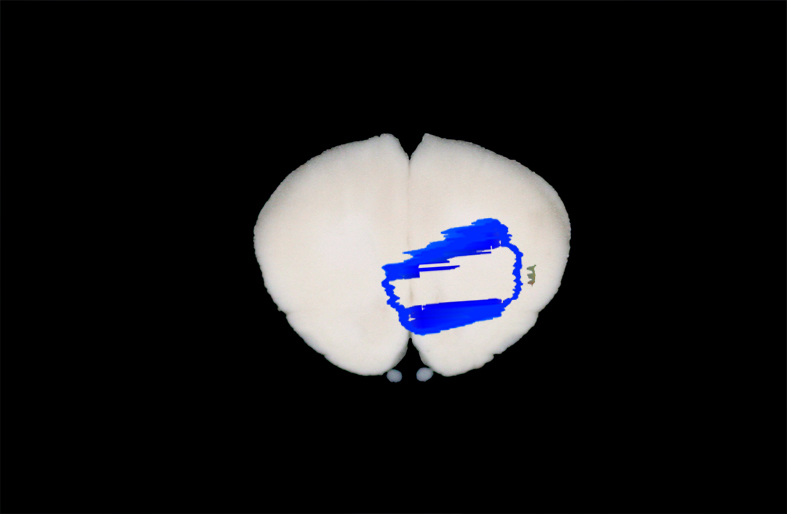

Supplement: Supplementary file 2 — Dataset 1 [file 41598_2019_55585_MOESM2_ESM.zip › 153.jpg]

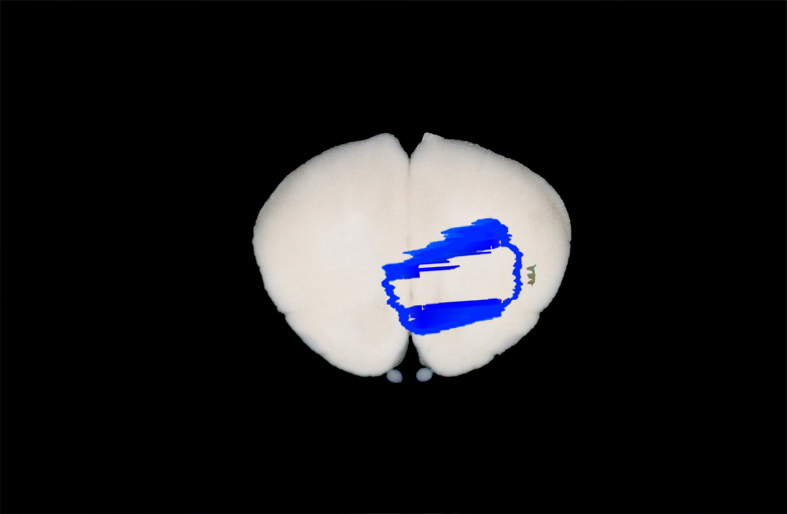

Supplement: Supplementary file 2 — Dataset 1 [file 41598_2019_55585_MOESM2_ESM.zip › 154.jpg]

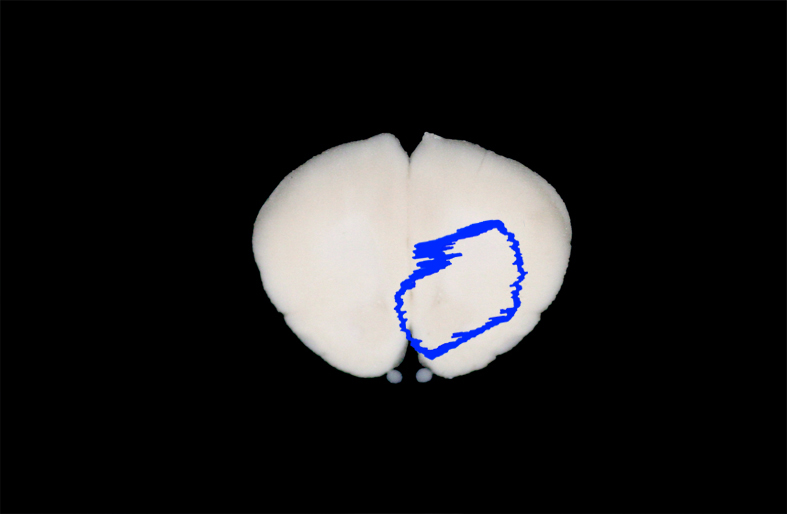

Supplement: Supplementary file 2 — Dataset 1 [file 41598_2019_55585_MOESM2_ESM.zip › 155.jpg]

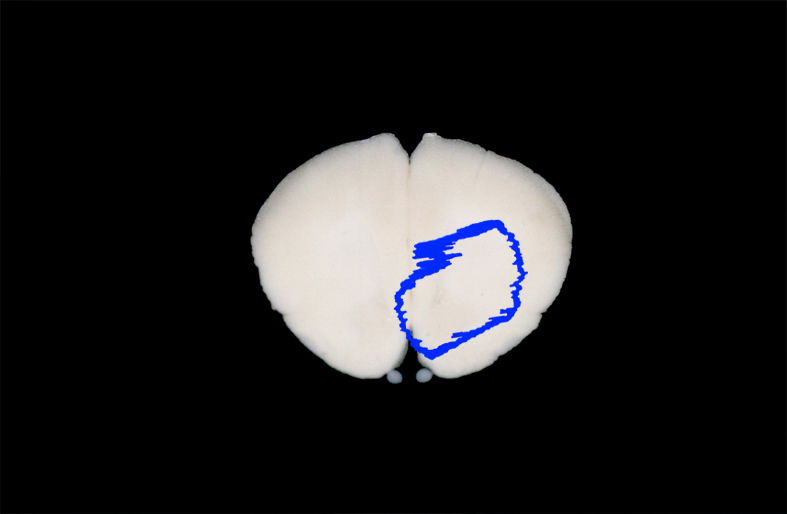

Supplement: Supplementary file 2 — Dataset 1 [file 41598_2019_55585_MOESM2_ESM.zip › 156.jpg]

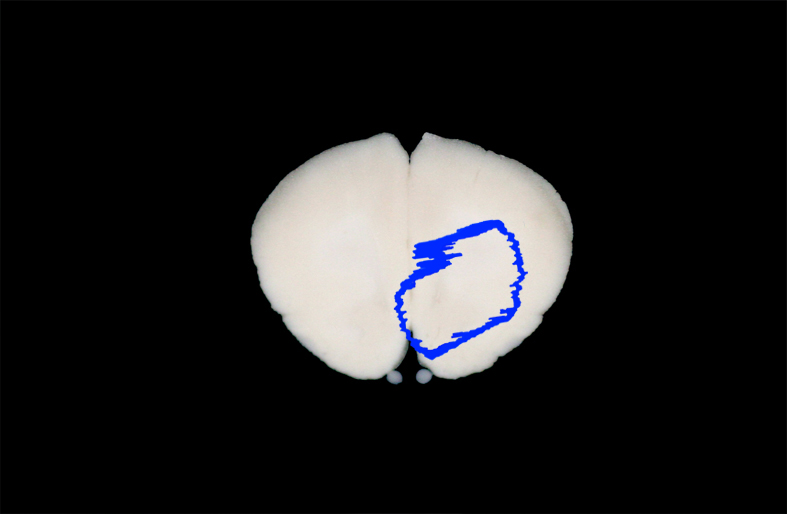

Supplement: Supplementary file 2 — Dataset 1 [file 41598_2019_55585_MOESM2_ESM.zip › 157.jpg]

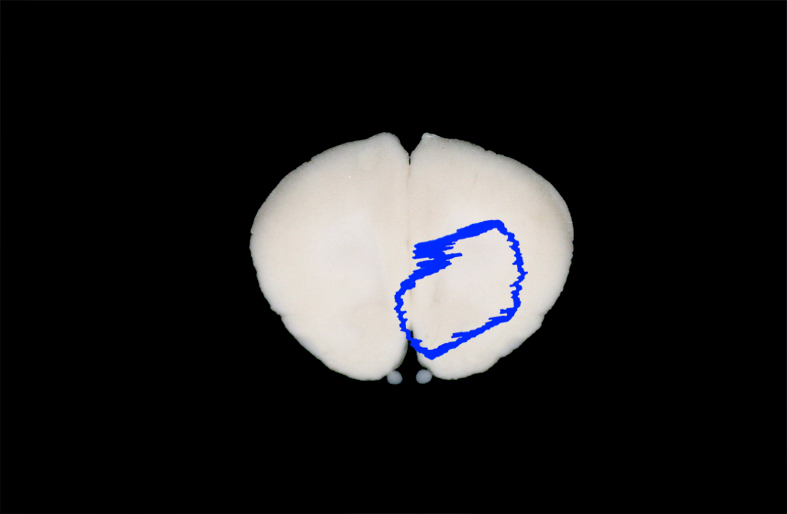

Supplement: Supplementary file 2 — Dataset 1 [file 41598_2019_55585_MOESM2_ESM.zip › 158.jpg]

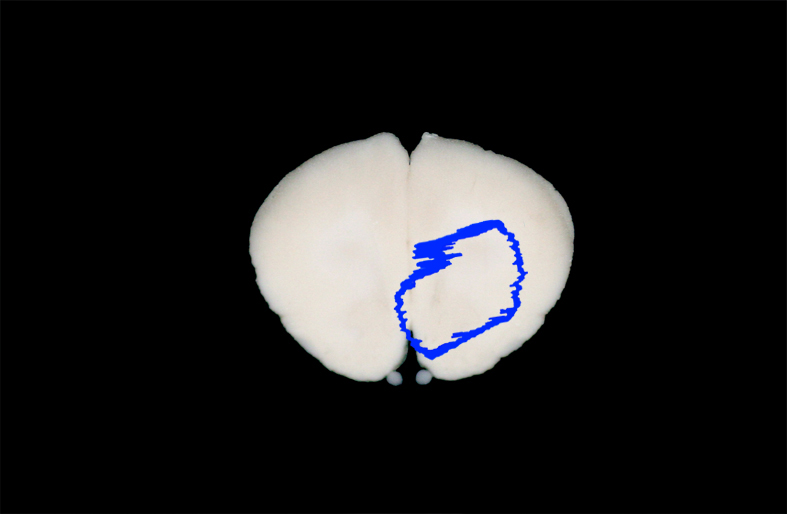

Supplement: Supplementary file 2 — Dataset 1 [file 41598_2019_55585_MOESM2_ESM.zip › 159.jpg]

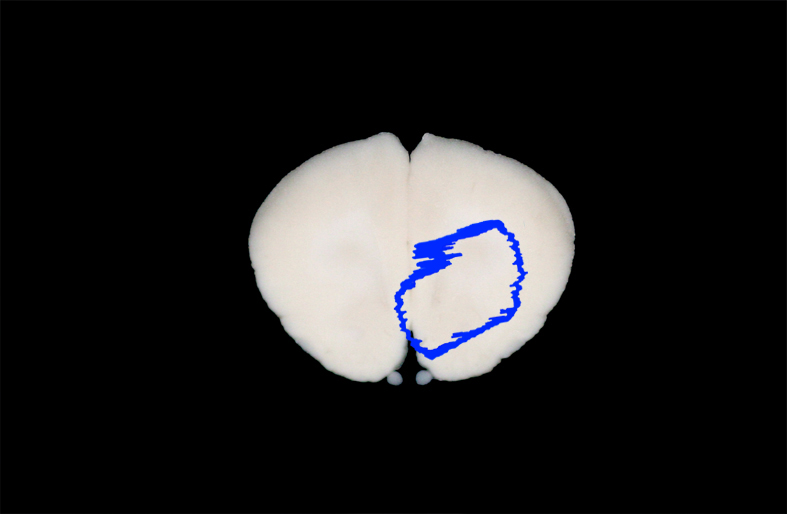

Supplement: Supplementary file 2 — Dataset 1 [file 41598_2019_55585_MOESM2_ESM.zip › 160.jpg]

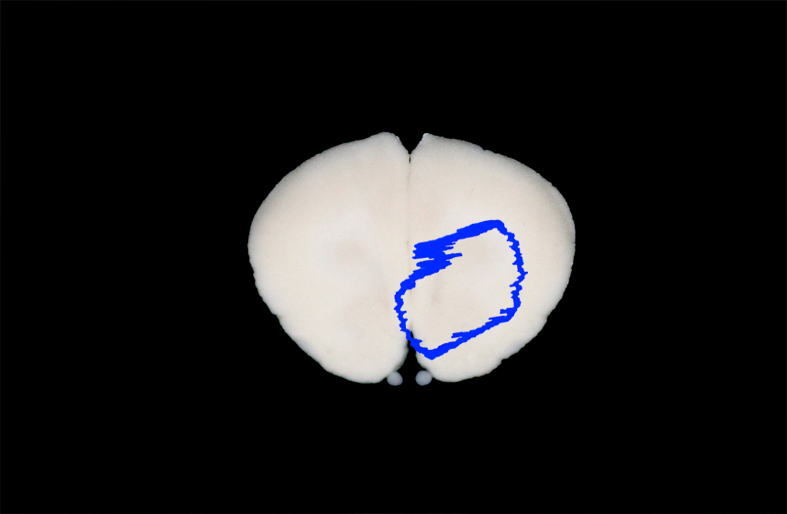

Supplement: Supplementary file 2 — Dataset 1 [file 41598_2019_55585_MOESM2_ESM.zip › 161.jpg]

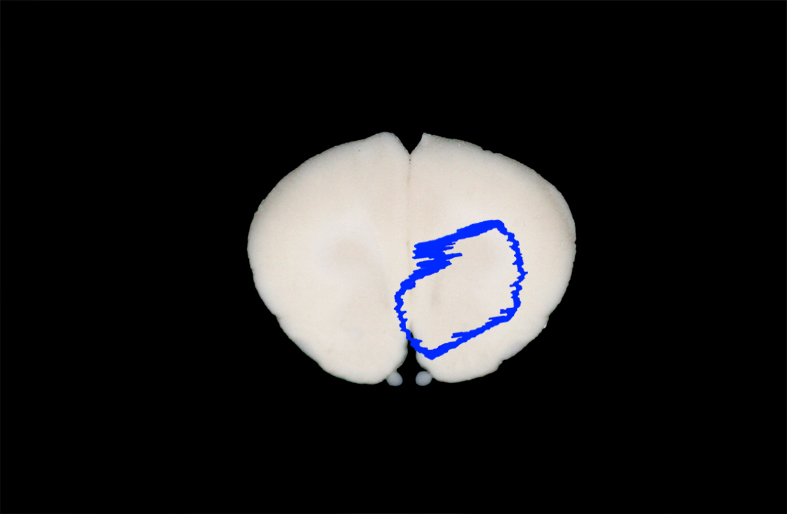

Supplement: Supplementary file 2 — Dataset 1 [file 41598_2019_55585_MOESM2_ESM.zip › 162.jpg]

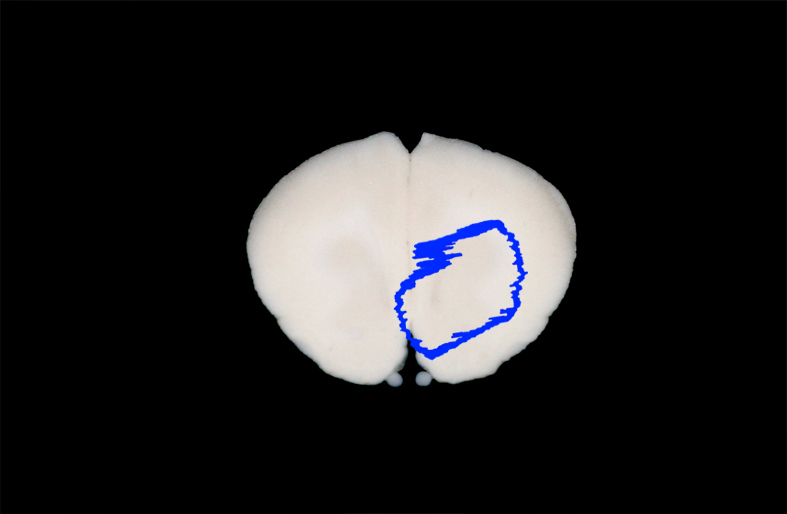

Supplement: Supplementary file 2 — Dataset 1 [file 41598_2019_55585_MOESM2_ESM.zip › 163.jpg]

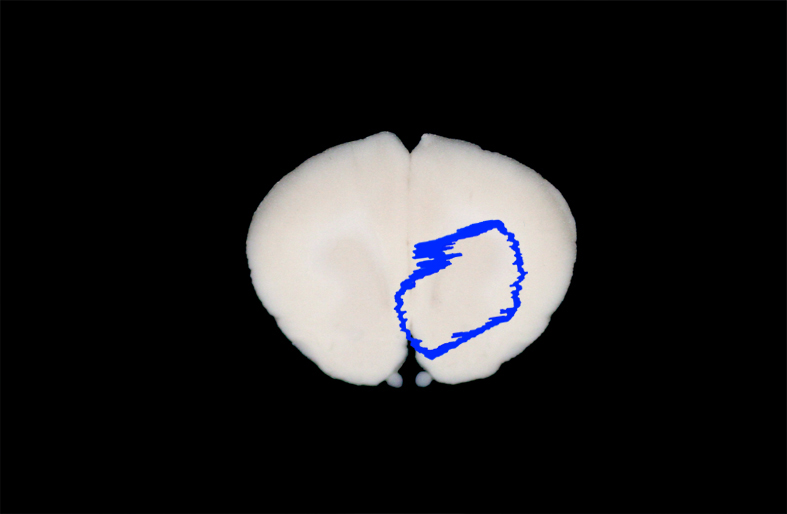

Supplement: Supplementary file 2 — Dataset 1 [file 41598_2019_55585_MOESM2_ESM.zip › 164.jpg]

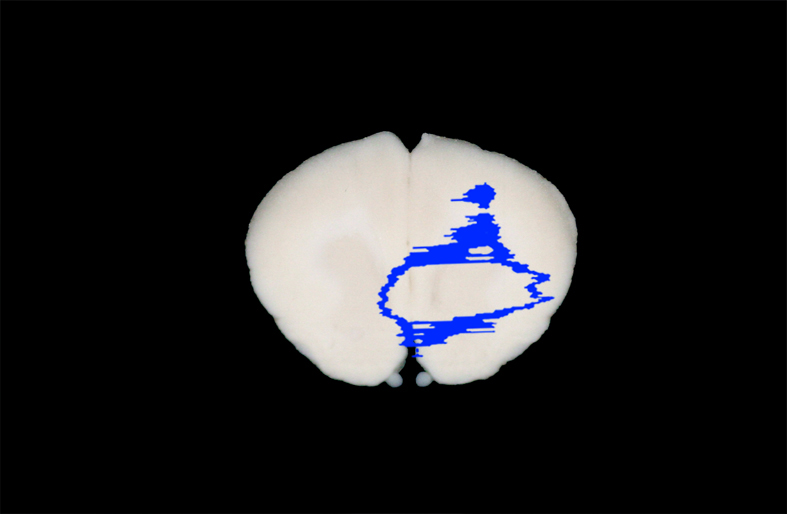

Supplement: Supplementary file 2 — Dataset 1 [file 41598_2019_55585_MOESM2_ESM.zip › 165.jpg]

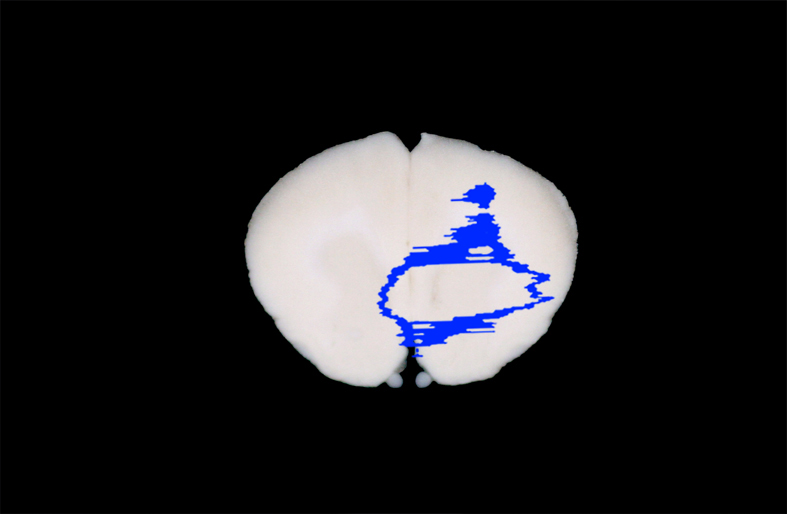

Supplement: Supplementary file 2 — Dataset 1 [file 41598_2019_55585_MOESM2_ESM.zip › 166.jpg]

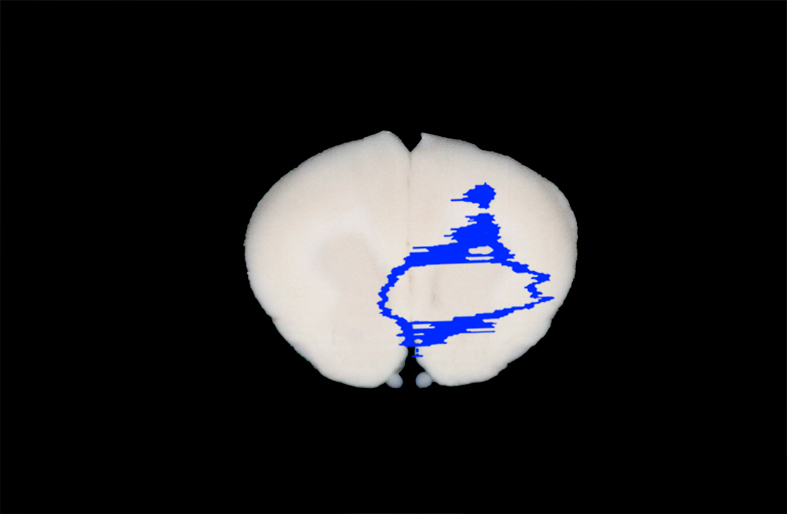

Supplement: Supplementary file 2 — Dataset 1 [file 41598_2019_55585_MOESM2_ESM.zip › 167.jpg]

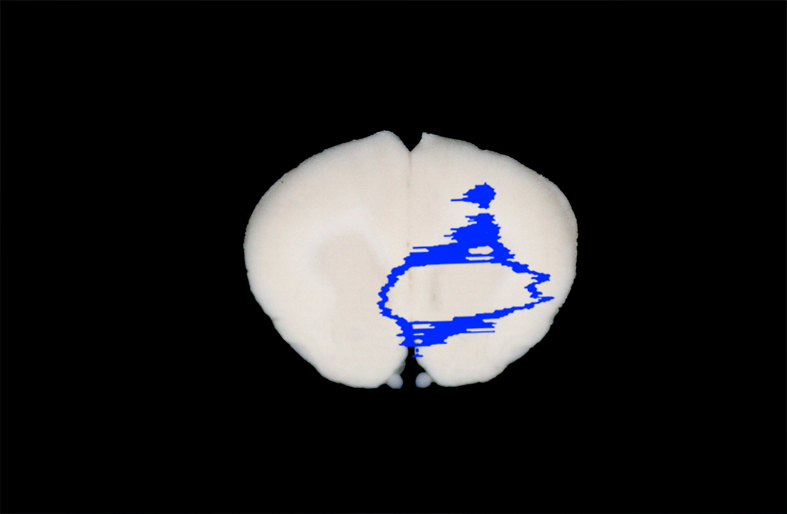

Supplement: Supplementary file 2 — Dataset 1 [file 41598_2019_55585_MOESM2_ESM.zip › 168.jpg]

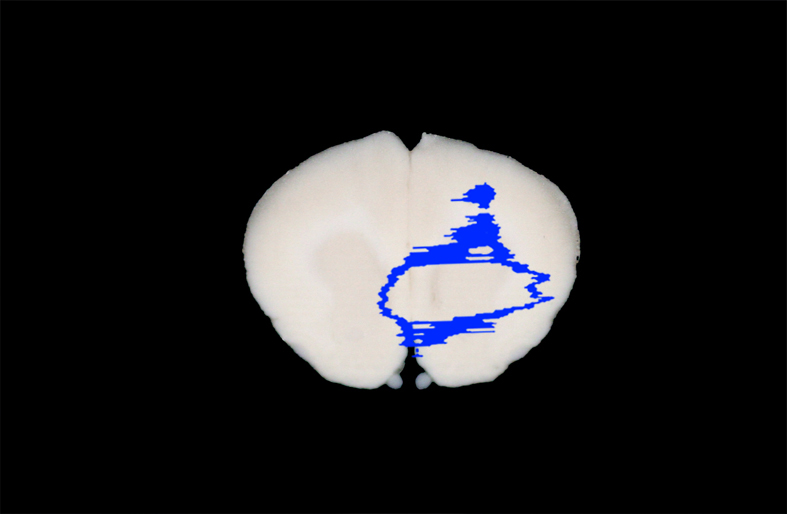

Supplement: Supplementary file 2 — Dataset 1 [file 41598_2019_55585_MOESM2_ESM.zip › 169.jpg]

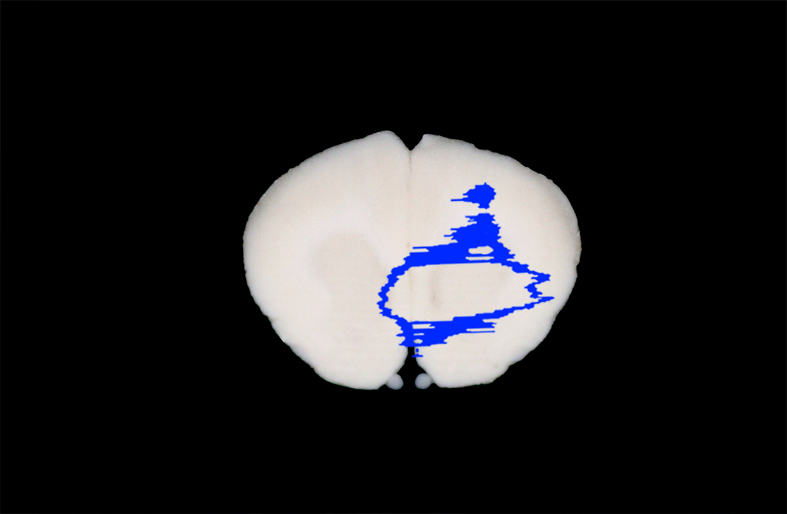

Supplement: Supplementary file 2 — Dataset 1 [file 41598_2019_55585_MOESM2_ESM.zip › 170.jpg]

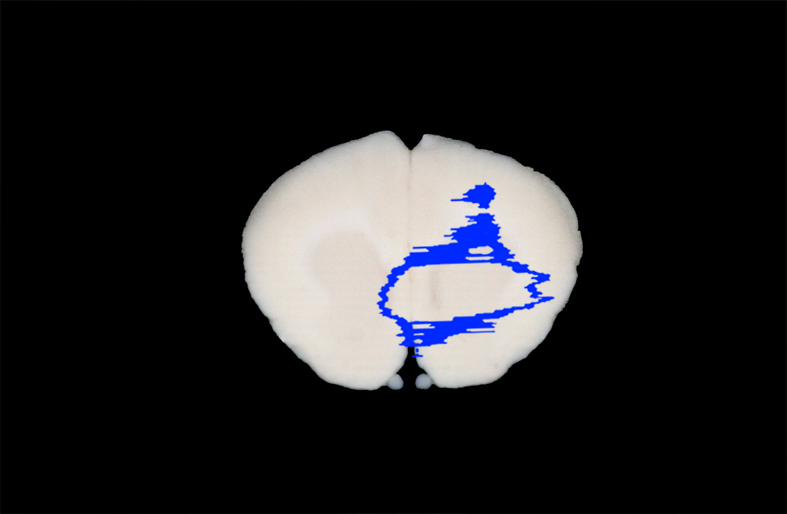

Supplement: Supplementary file 2 — Dataset 1 [file 41598_2019_55585_MOESM2_ESM.zip › 171.jpg]

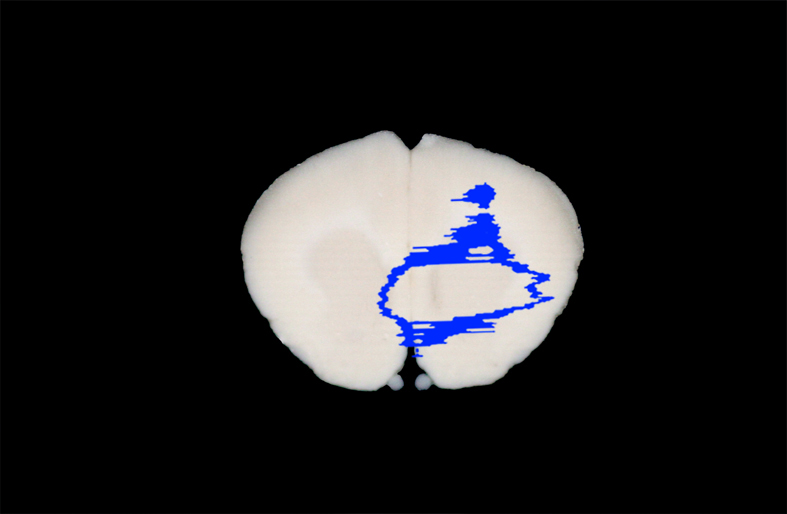

Supplement: Supplementary file 2 — Dataset 1 [file 41598_2019_55585_MOESM2_ESM.zip › 172.jpg]

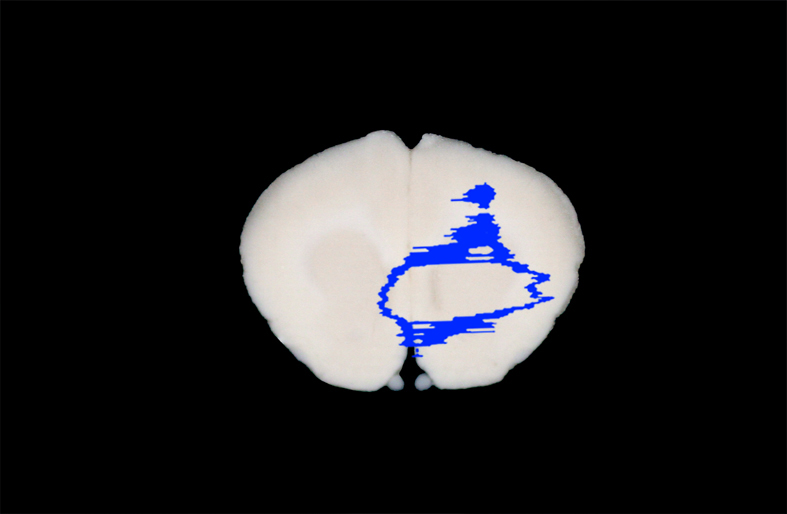

Supplement: Supplementary file 2 — Dataset 1 [file 41598_2019_55585_MOESM2_ESM.zip › 173.jpg]

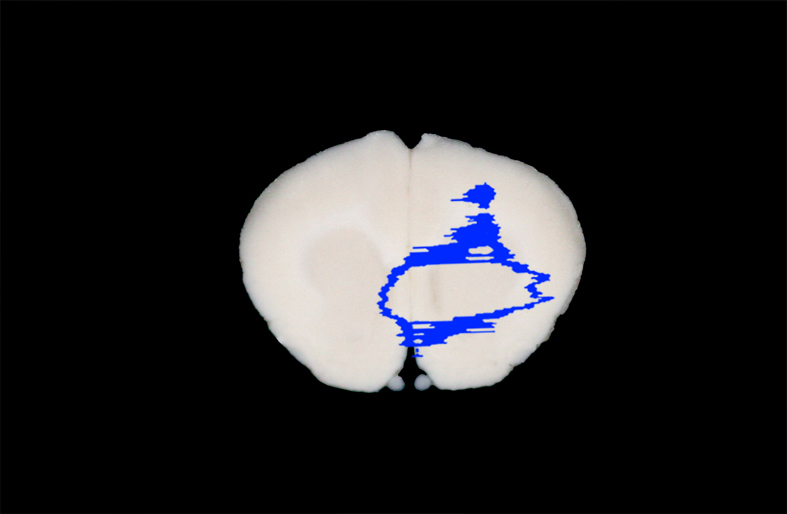

Supplement: Supplementary file 2 — Dataset 1 [file 41598_2019_55585_MOESM2_ESM.zip › 174.jpg]

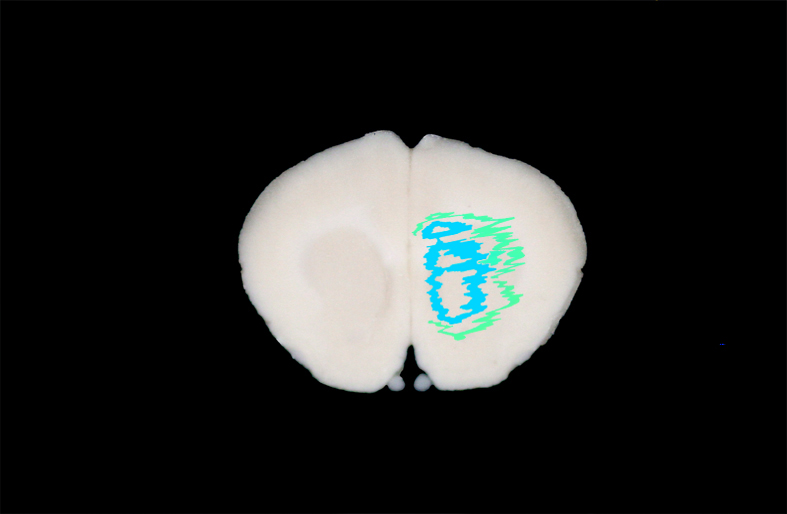

Supplement: Supplementary file 2 — Dataset 1 [file 41598_2019_55585_MOESM2_ESM.zip › 175.jpg]

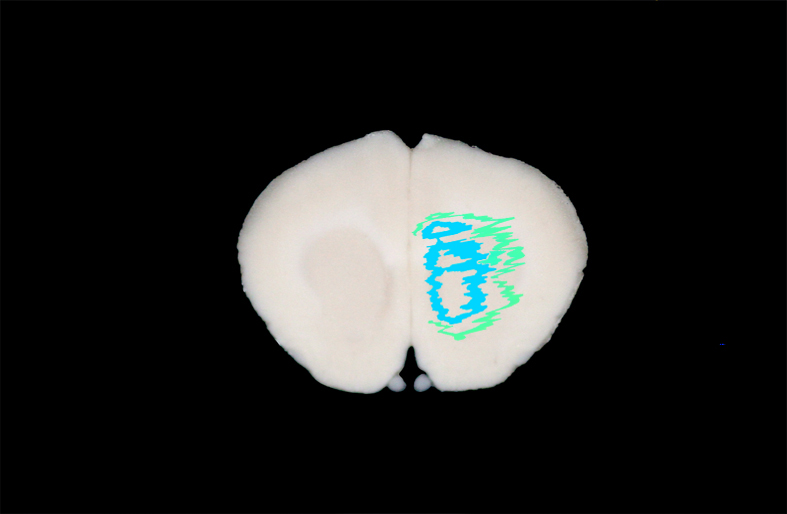

Supplement: Supplementary file 2 — Dataset 1 [file 41598_2019_55585_MOESM2_ESM.zip › 176.jpg]

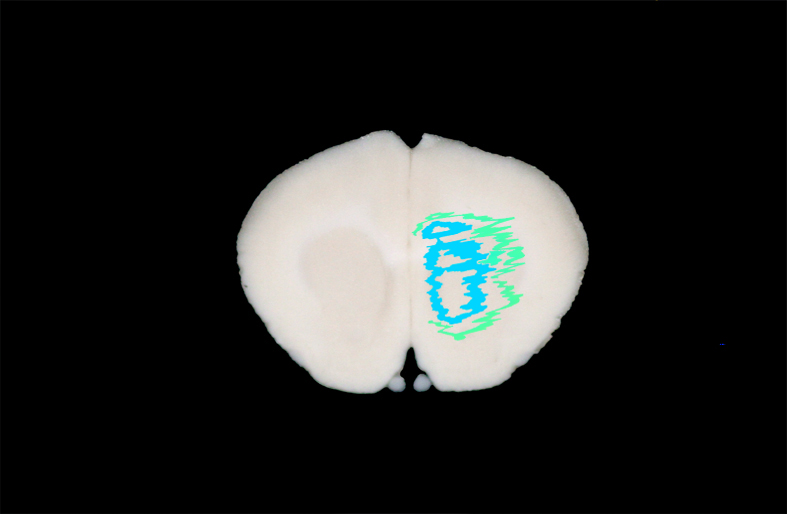

Supplement: Supplementary file 2 — Dataset 1 [file 41598_2019_55585_MOESM2_ESM.zip › 177.jpg]

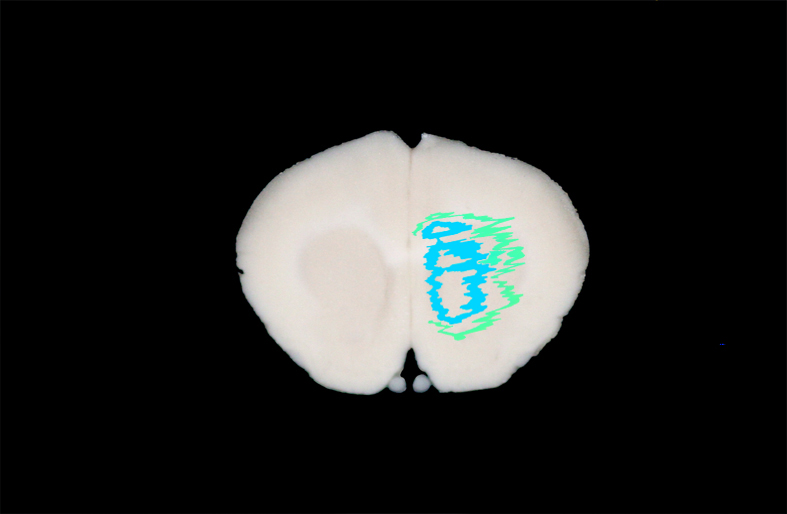

Supplement: Supplementary file 2 — Dataset 1 [file 41598_2019_55585_MOESM2_ESM.zip › 178.jpg]

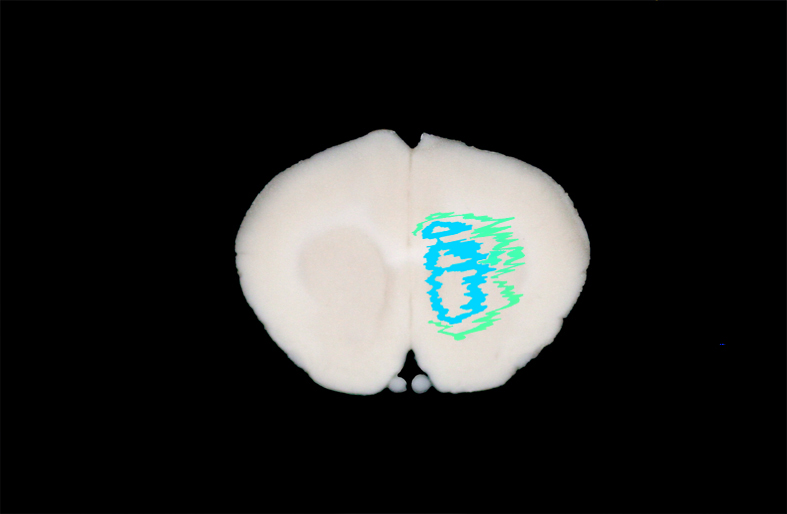

Supplement: Supplementary file 2 — Dataset 1 [file 41598_2019_55585_MOESM2_ESM.zip › 179.jpg]

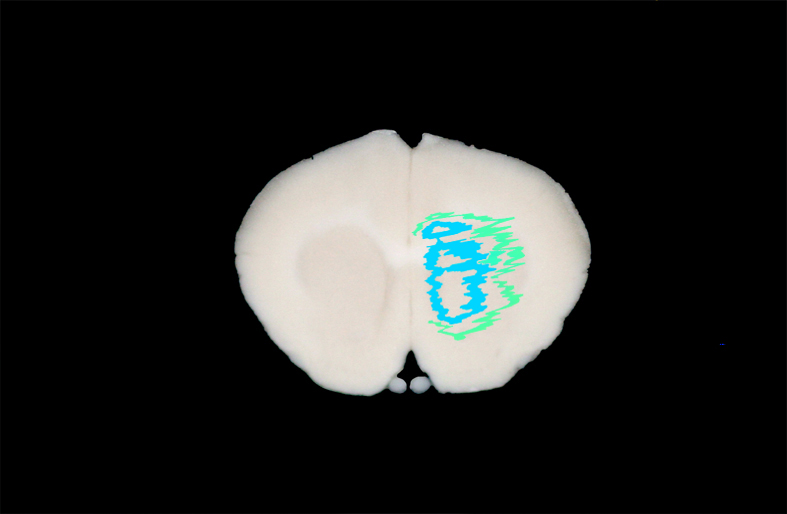

Supplement: Supplementary file 2 — Dataset 1 [file 41598_2019_55585_MOESM2_ESM.zip › 180.jpg]

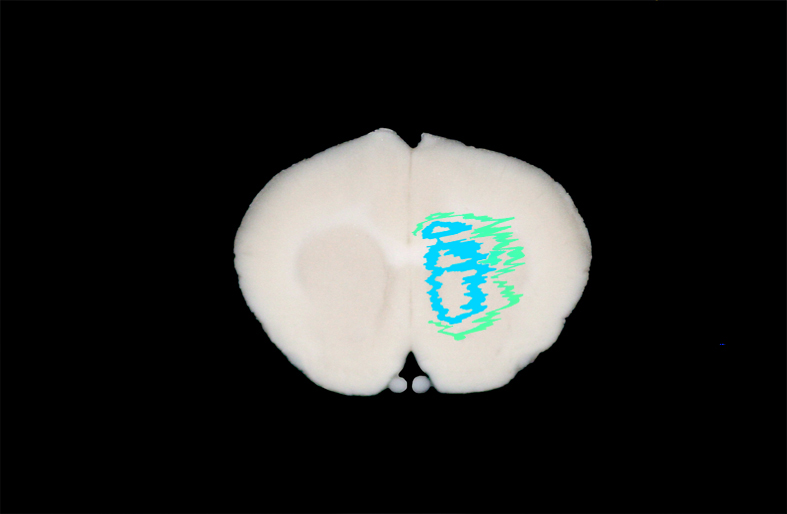

Supplement: Supplementary file 2 — Dataset 1 [file 41598_2019_55585_MOESM2_ESM.zip › 181.jpg]

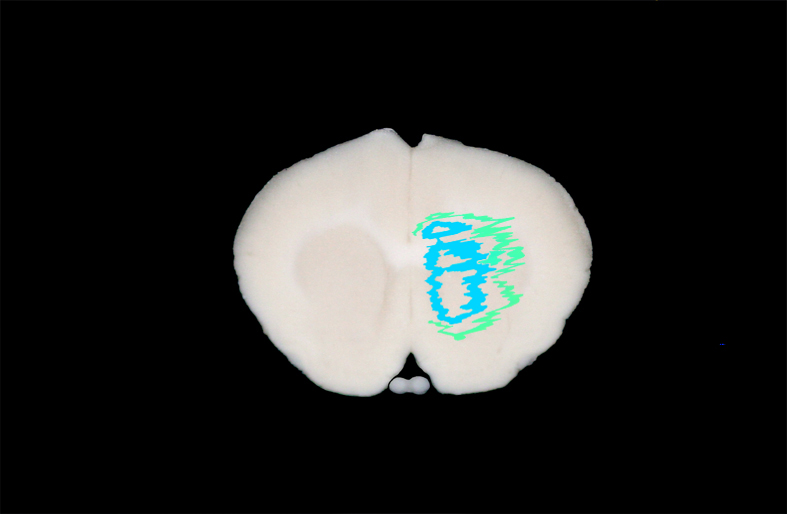

Supplement: Supplementary file 2 — Dataset 1 [file 41598_2019_55585_MOESM2_ESM.zip › 182.jpg]

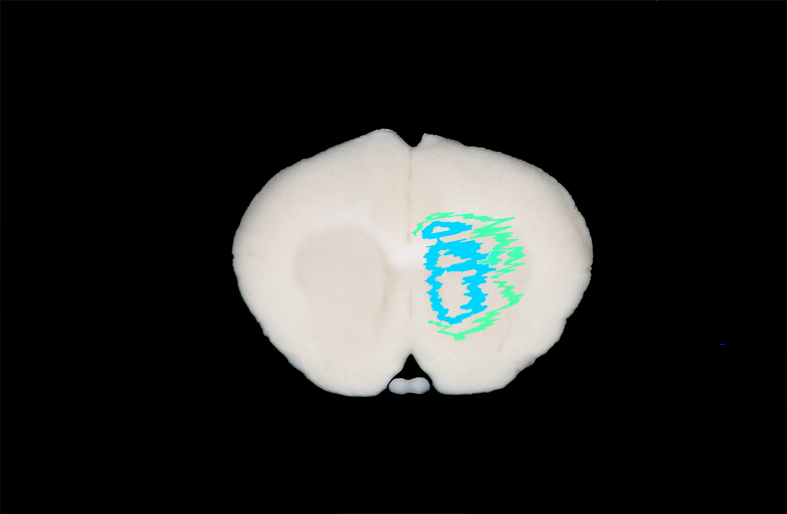

Supplement: Supplementary file 2 — Dataset 1 [file 41598_2019_55585_MOESM2_ESM.zip › 183.jpg]

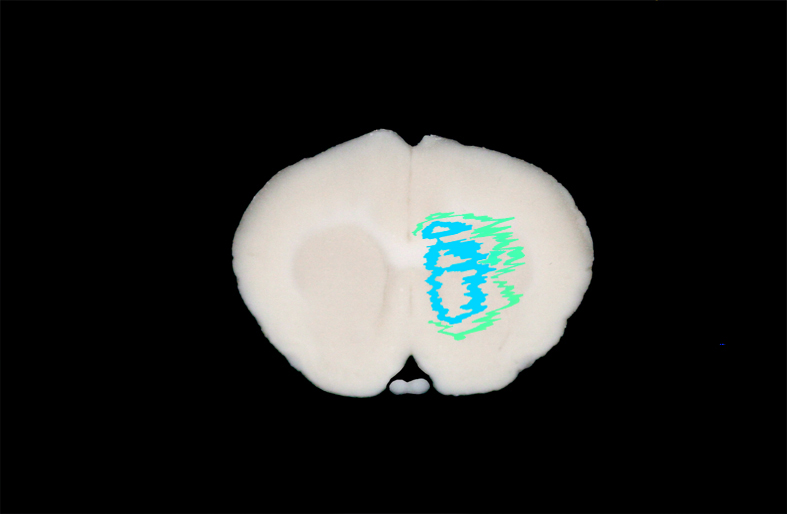

Supplement: Supplementary file 2 — Dataset 1 [file 41598_2019_55585_MOESM2_ESM.zip › 184.jpg]

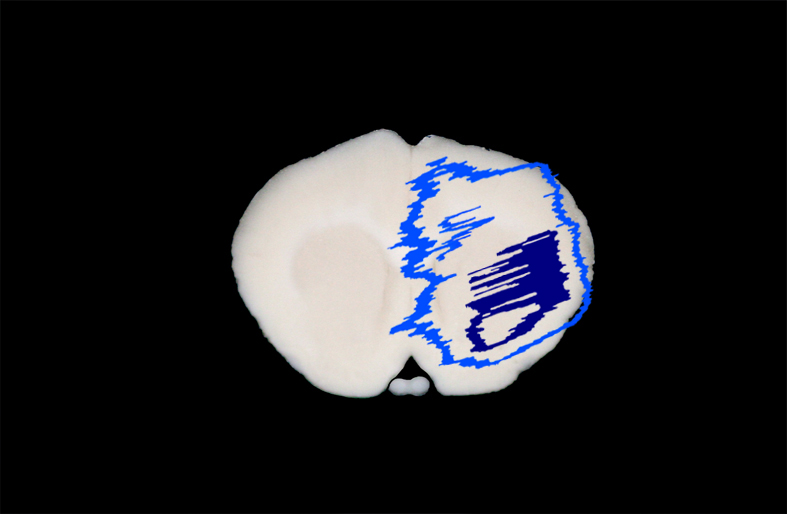

Supplement: Supplementary file 2 — Dataset 1 [file 41598_2019_55585_MOESM2_ESM.zip › 185.jpg]

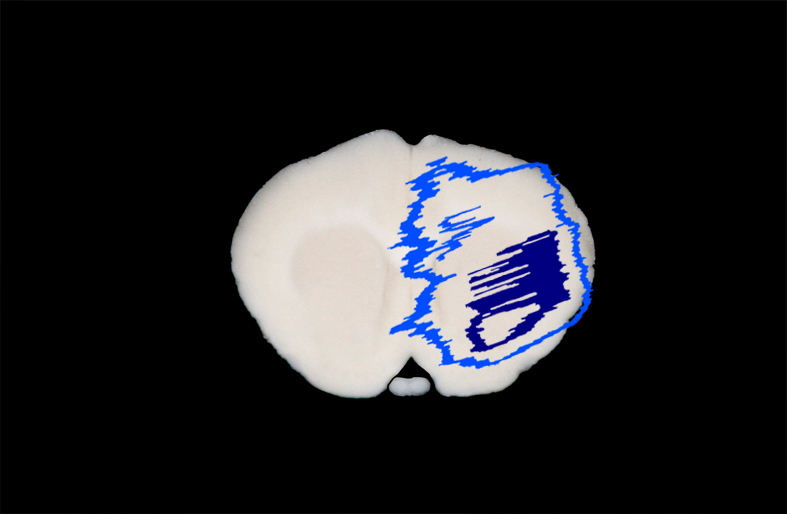

Supplement: Supplementary file 2 — Dataset 1 [file 41598_2019_55585_MOESM2_ESM.zip › 186.jpg]

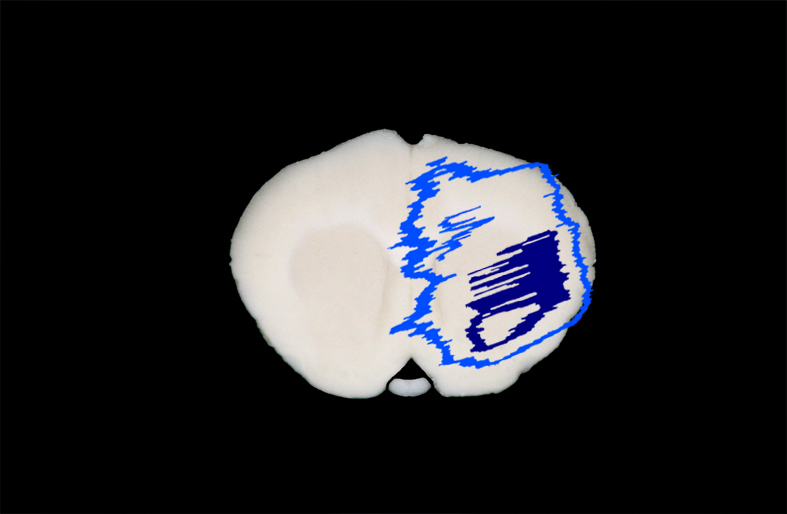

Supplement: Supplementary file 2 — Dataset 1 [file 41598_2019_55585_MOESM2_ESM.zip › 187.jpg]

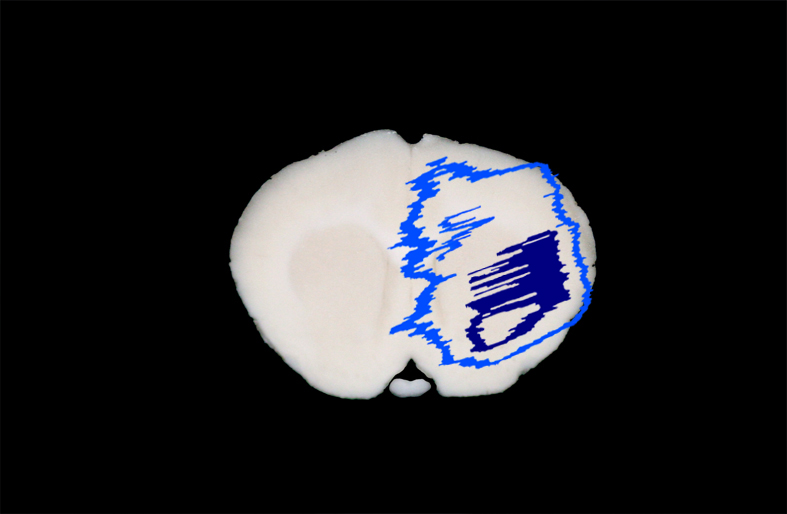

Supplement: Supplementary file 2 — Dataset 1 [file 41598_2019_55585_MOESM2_ESM.zip › 188.jpg]

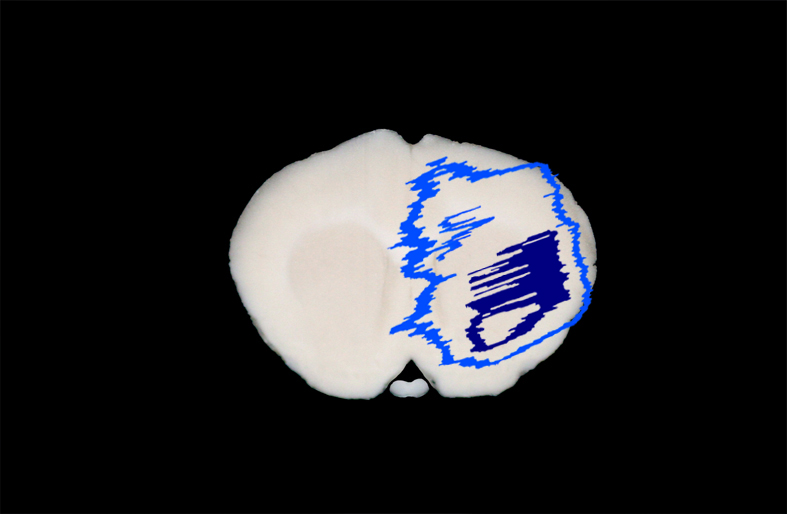

Supplement: Supplementary file 2 — Dataset 1 [file 41598_2019_55585_MOESM2_ESM.zip › 189.jpg]

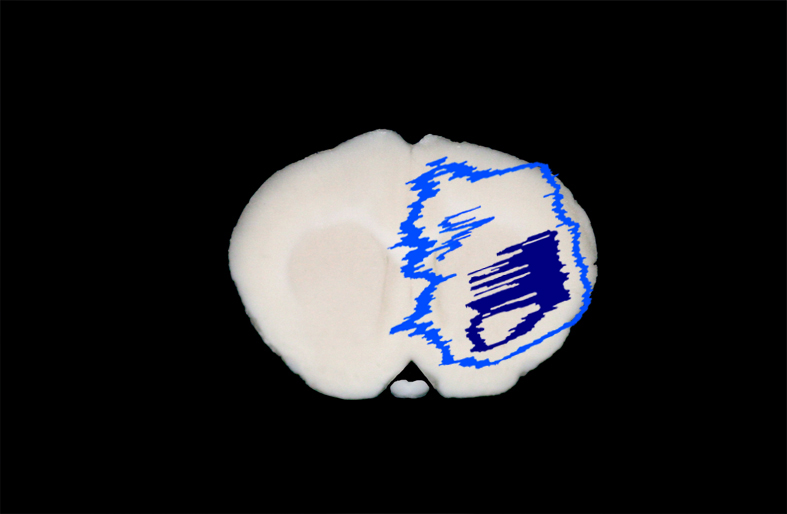

Supplement: Supplementary file 2 — Dataset 1 [file 41598_2019_55585_MOESM2_ESM.zip › 190.jpg]

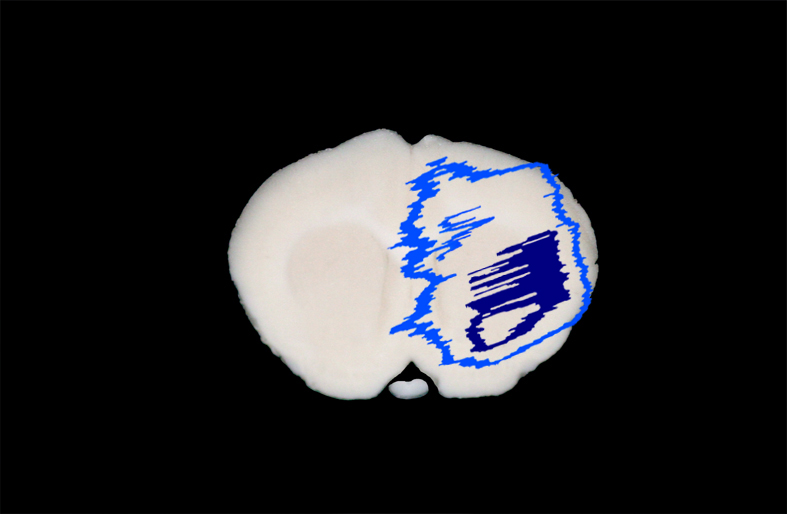

Supplement: Supplementary file 2 — Dataset 1 [file 41598_2019_55585_MOESM2_ESM.zip › 191.jpg]

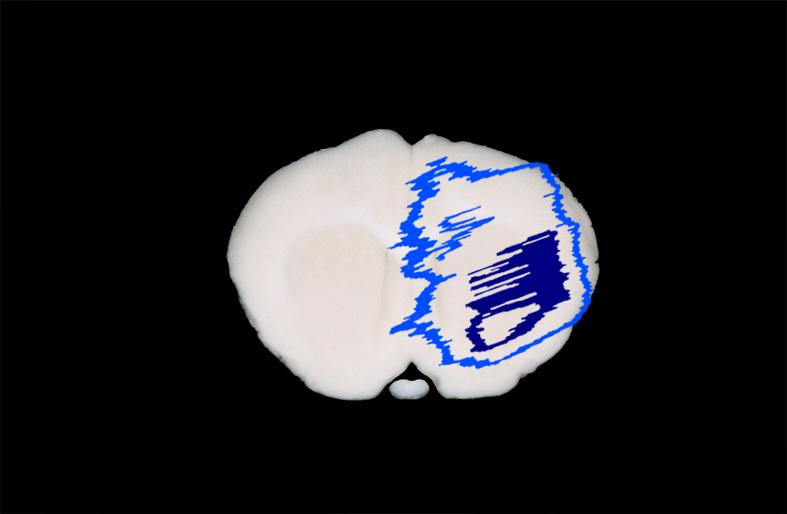

Supplement: Supplementary file 2 — Dataset 1 [file 41598_2019_55585_MOESM2_ESM.zip › 192.jpg]

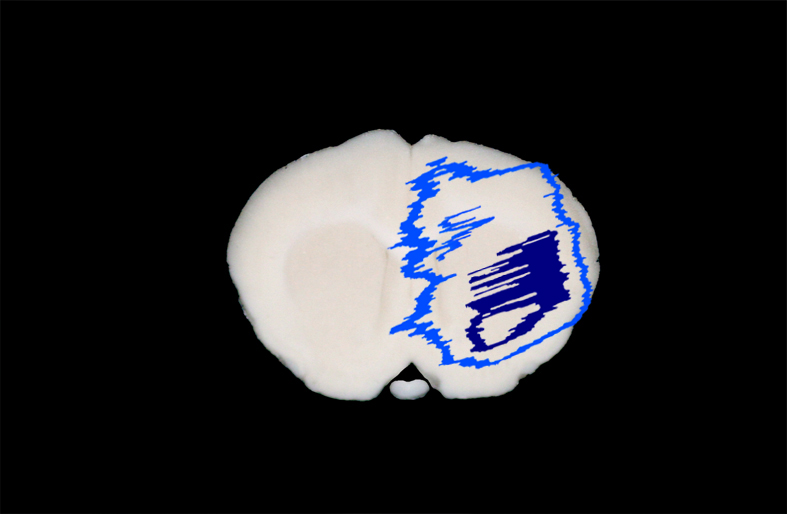

Supplement: Supplementary file 2 — Dataset 1 [file 41598_2019_55585_MOESM2_ESM.zip › 193.jpg]

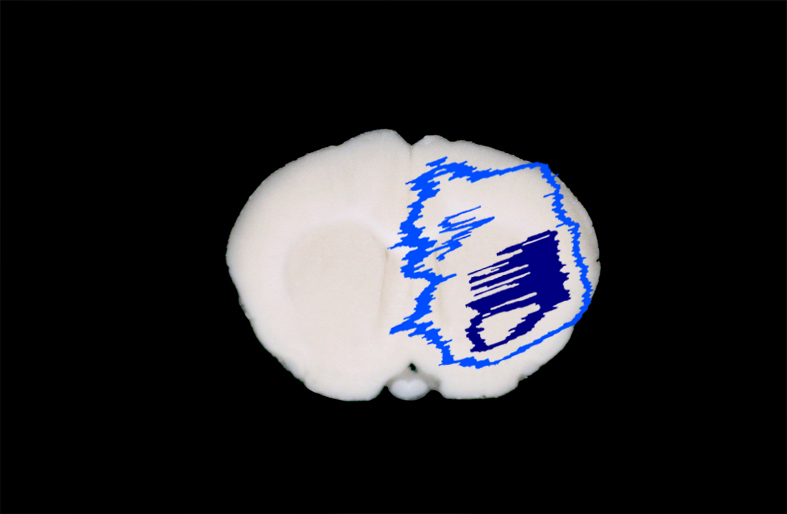

Supplement: Supplementary file 2 — Dataset 1 [file 41598_2019_55585_MOESM2_ESM.zip › 194.jpg]

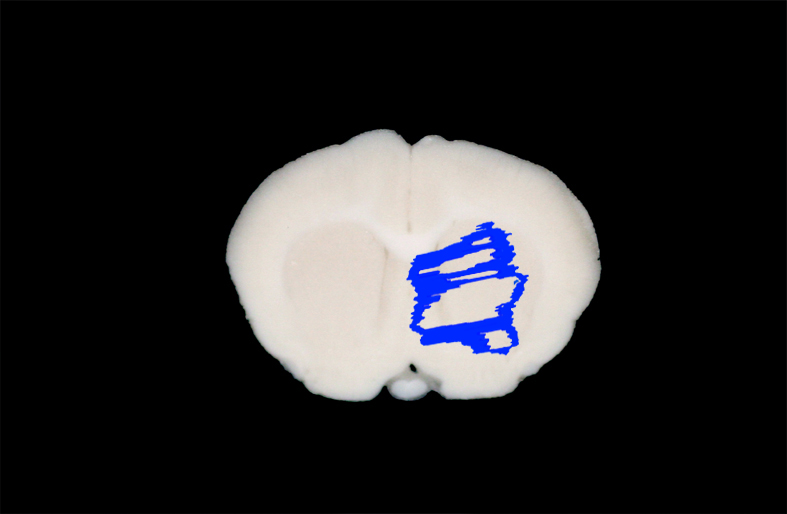

Supplement: Supplementary file 2 — Dataset 1 [file 41598_2019_55585_MOESM2_ESM.zip › 195.jpg]

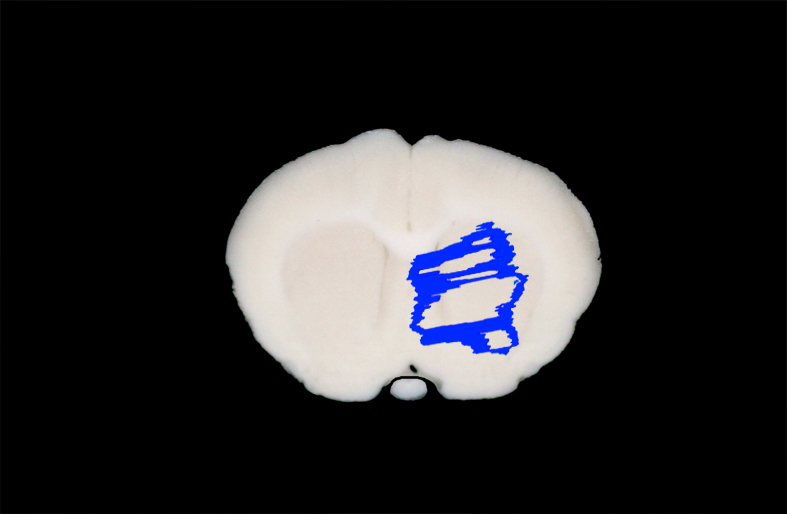

Supplement: Supplementary file 2 — Dataset 1 [file 41598_2019_55585_MOESM2_ESM.zip › 196.jpg]

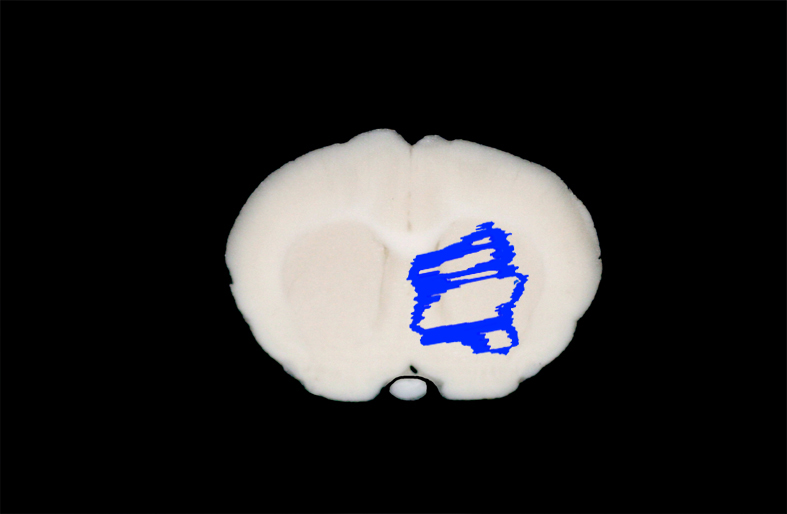

Supplement: Supplementary file 2 — Dataset 1 [file 41598_2019_55585_MOESM2_ESM.zip › 197.jpg]

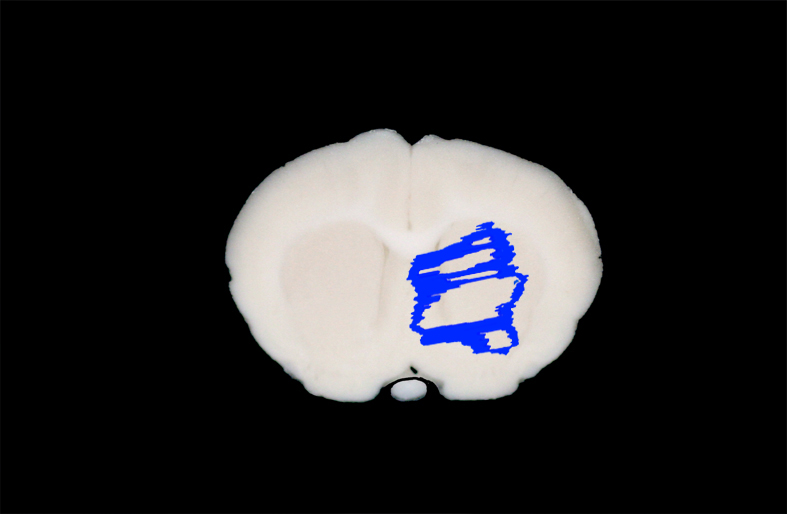

Supplement: Supplementary file 2 — Dataset 1 [file 41598_2019_55585_MOESM2_ESM.zip › 198.jpg]

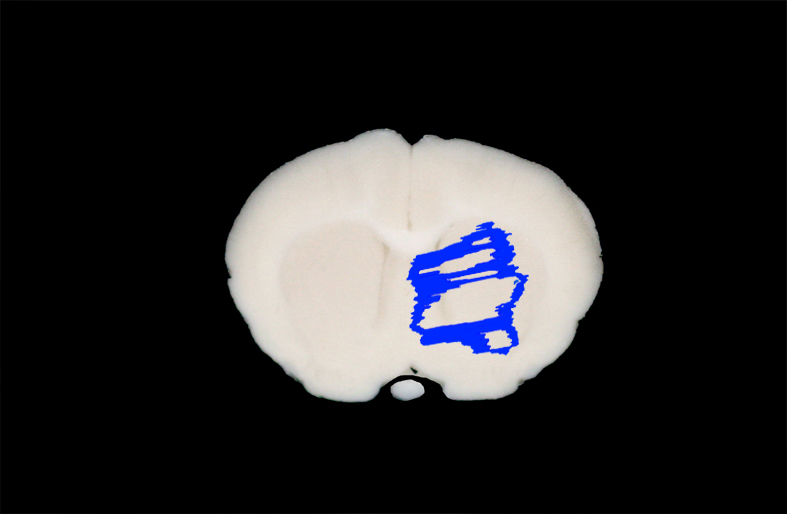

Supplement: Supplementary file 2 — Dataset 1 [file 41598_2019_55585_MOESM2_ESM.zip › 199.jpg]

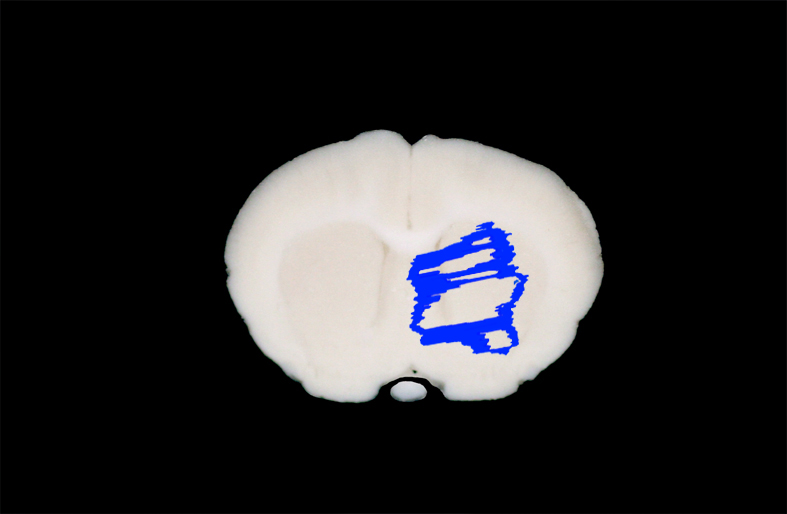

Supplement: Supplementary file 2 — Dataset 1 [file 41598_2019_55585_MOESM2_ESM.zip › 200.jpg]
