# Supplementary figures and images for: Construction of 3D-rendering imaging of an ischemic rat brain model using the planar FMMD technique (part 1 of 2)
Source: Sci Rep. 2019 Dec 13;9:19050. doi: 10.1038/s41598-019-55585-x (PMC6910971; doi:10.1038/s41598-019-55585-x)

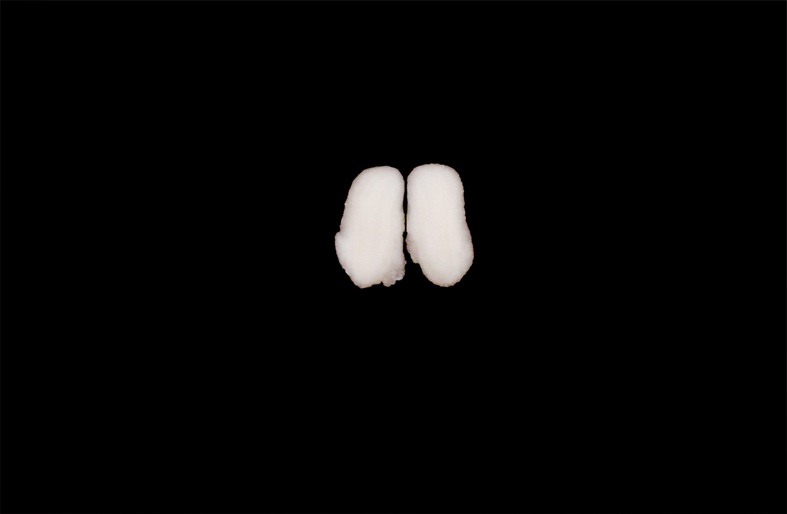

Supplement: Supplementary file 2 — Dataset 1 [file 41598_2019_55585_MOESM2_ESM.zip › 001.jpg]

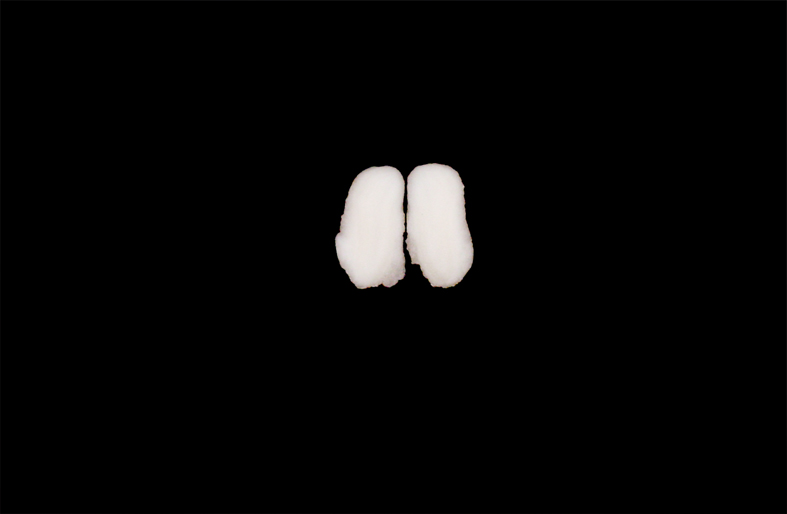

Supplement: Supplementary file 2 — Dataset 1 [file 41598_2019_55585_MOESM2_ESM.zip › 002.jpg]

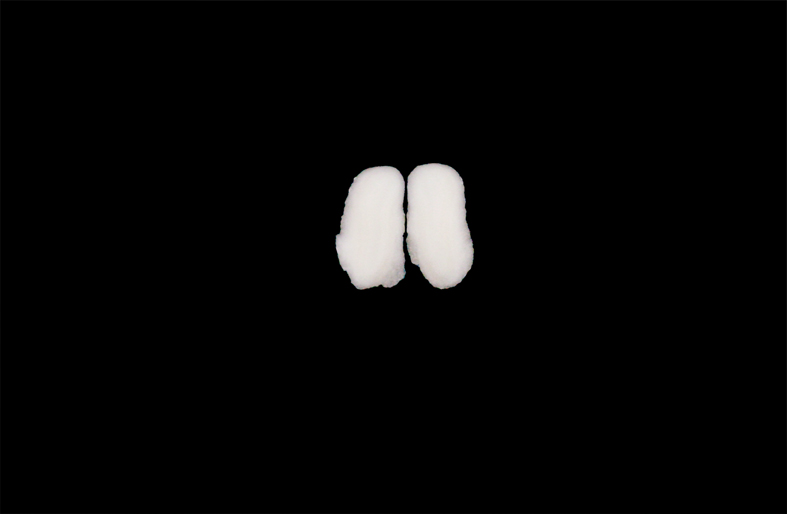

Supplement: Supplementary file 2 — Dataset 1 [file 41598_2019_55585_MOESM2_ESM.zip › 003.jpg]

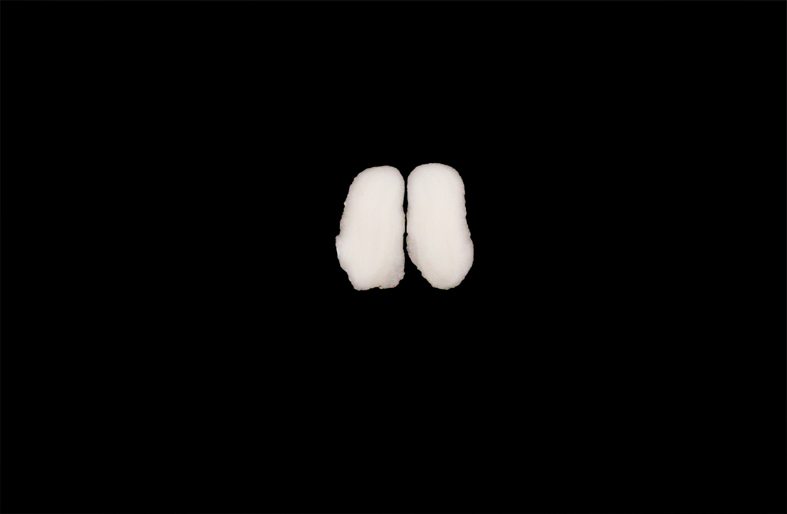

Supplement: Supplementary file 2 — Dataset 1 [file 41598_2019_55585_MOESM2_ESM.zip › 004.jpg]

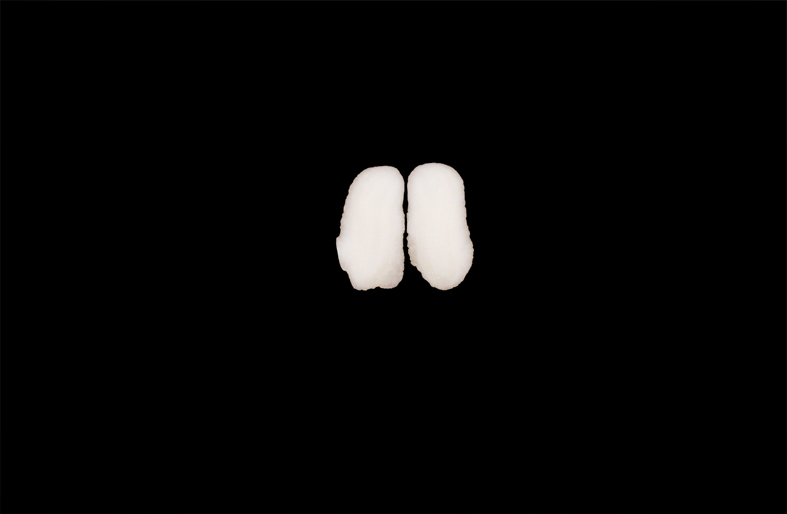

Supplement: Supplementary file 2 — Dataset 1 [file 41598_2019_55585_MOESM2_ESM.zip › 005.jpg]

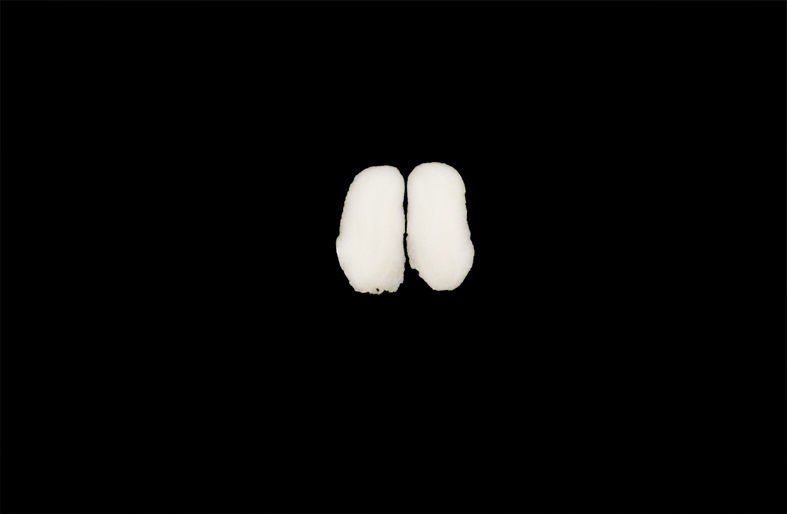

Supplement: Supplementary file 2 — Dataset 1 [file 41598_2019_55585_MOESM2_ESM.zip › 006.jpg]

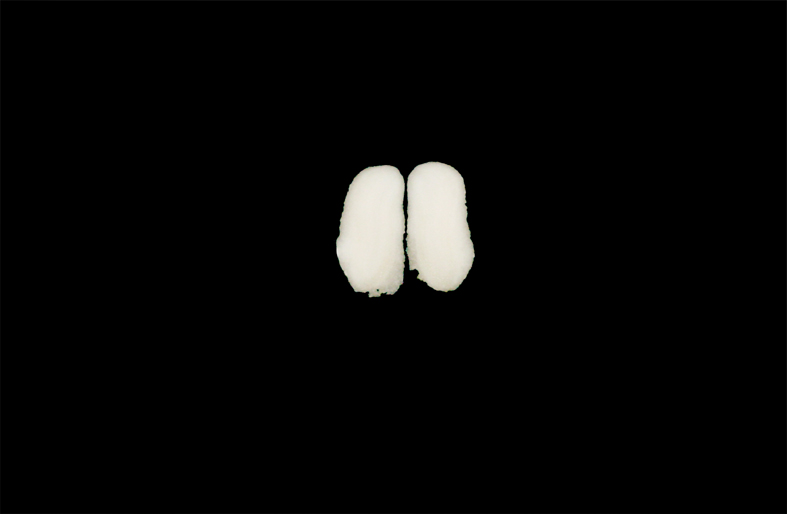

Supplement: Supplementary file 2 — Dataset 1 [file 41598_2019_55585_MOESM2_ESM.zip › 007.jpg]

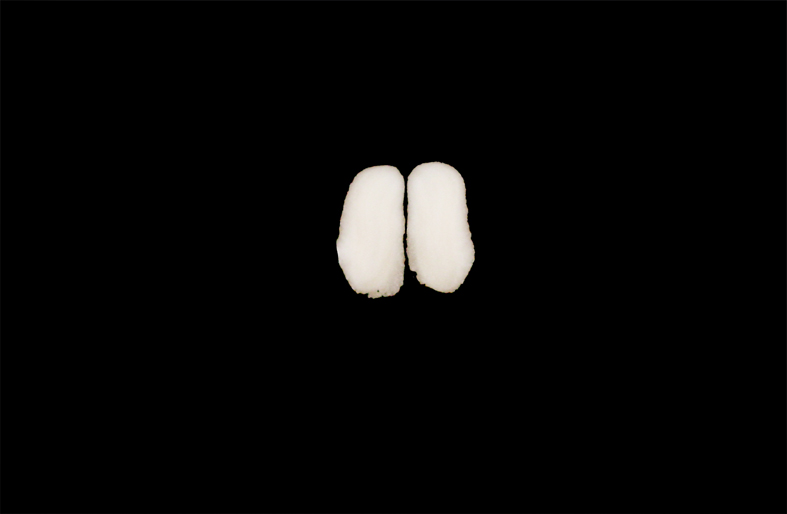

Supplement: Supplementary file 2 — Dataset 1 [file 41598_2019_55585_MOESM2_ESM.zip › 008.jpg]

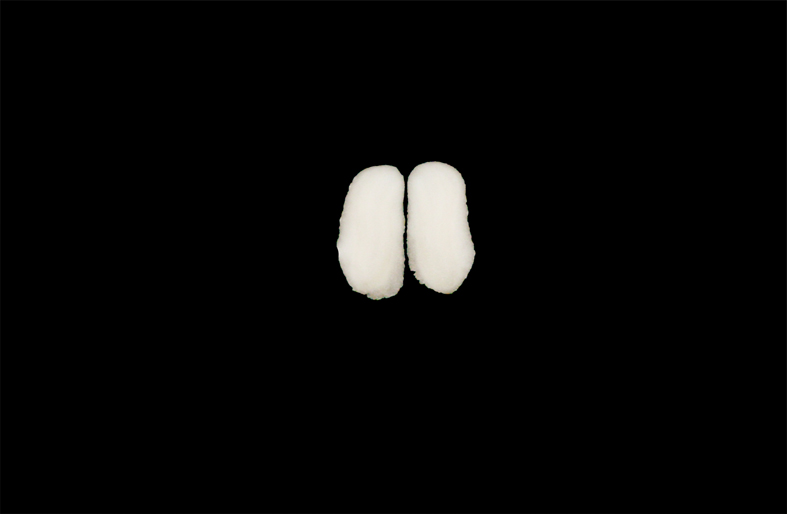

Supplement: Supplementary file 2 — Dataset 1 [file 41598_2019_55585_MOESM2_ESM.zip › 009.jpg]

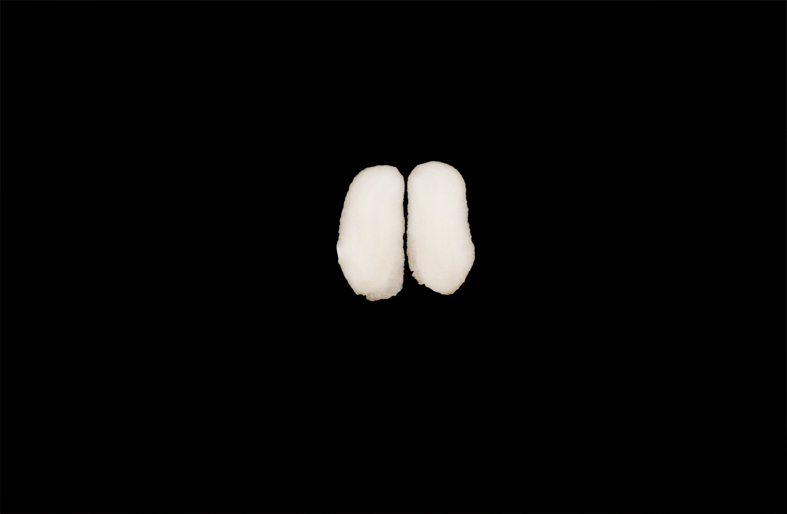

Supplement: Supplementary file 2 — Dataset 1 [file 41598_2019_55585_MOESM2_ESM.zip › 010.jpg]

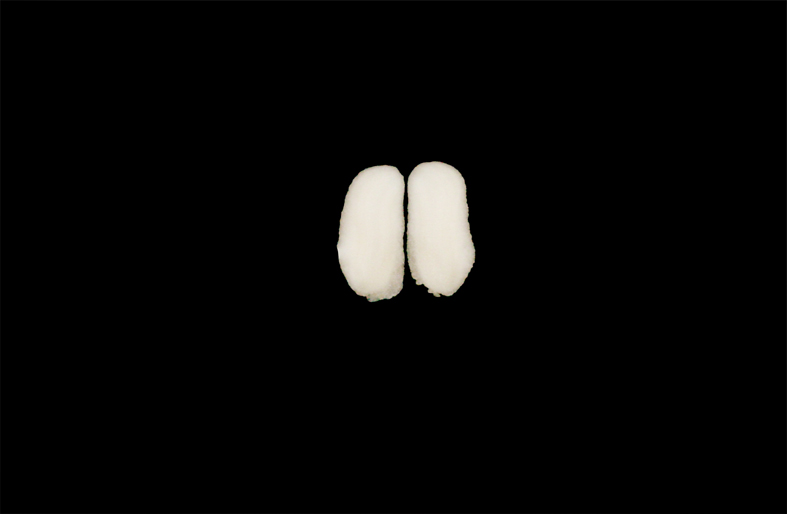

Supplement: Supplementary file 2 — Dataset 1 [file 41598_2019_55585_MOESM2_ESM.zip › 011.jpg]

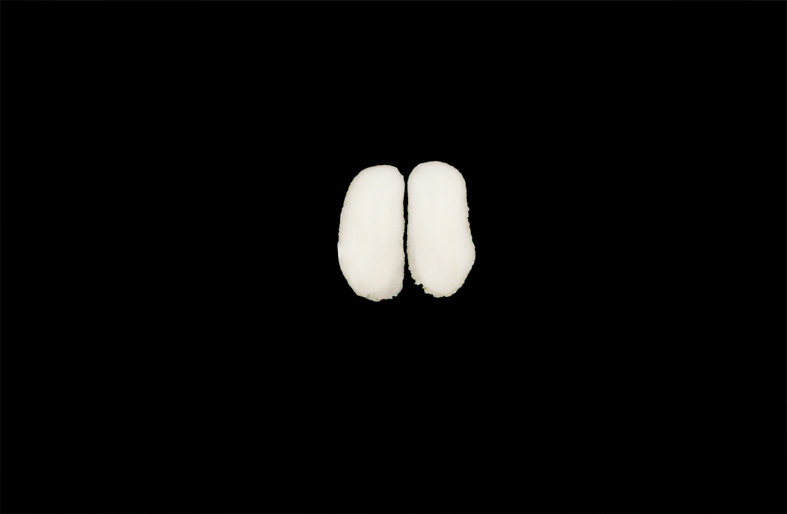

Supplement: Supplementary file 2 — Dataset 1 [file 41598_2019_55585_MOESM2_ESM.zip › 012.jpg]

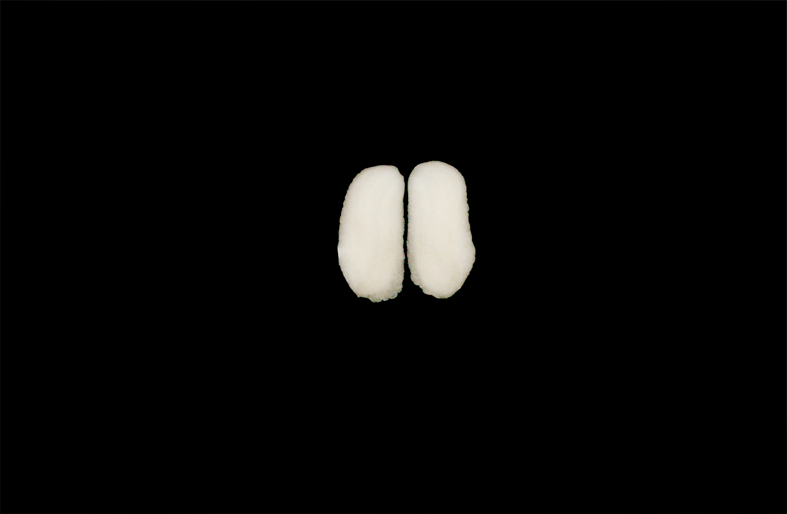

Supplement: Supplementary file 2 — Dataset 1 [file 41598_2019_55585_MOESM2_ESM.zip › 013.jpg]

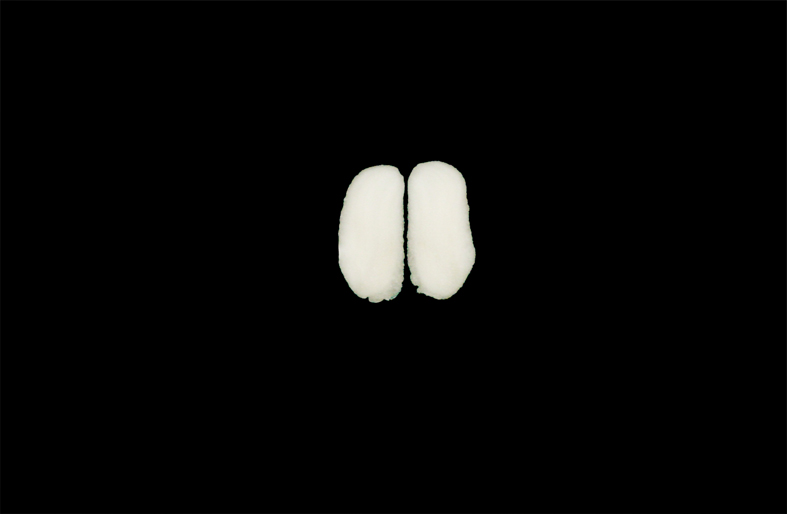

Supplement: Supplementary file 2 — Dataset 1 [file 41598_2019_55585_MOESM2_ESM.zip › 014.jpg]

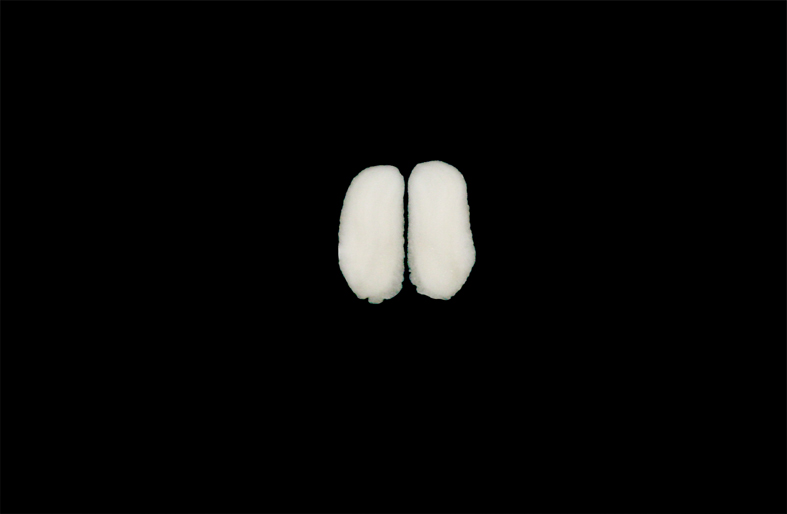

Supplement: Supplementary file 2 — Dataset 1 [file 41598_2019_55585_MOESM2_ESM.zip › 015.jpg]

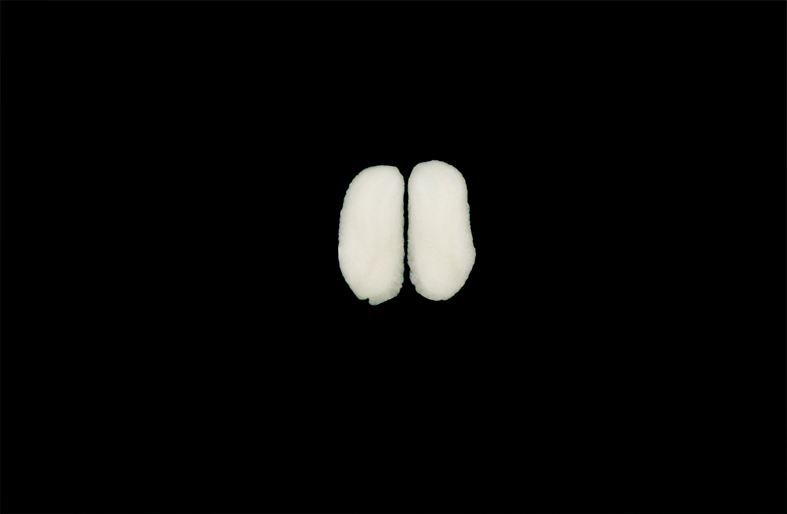

Supplement: Supplementary file 2 — Dataset 1 [file 41598_2019_55585_MOESM2_ESM.zip › 016.jpg]

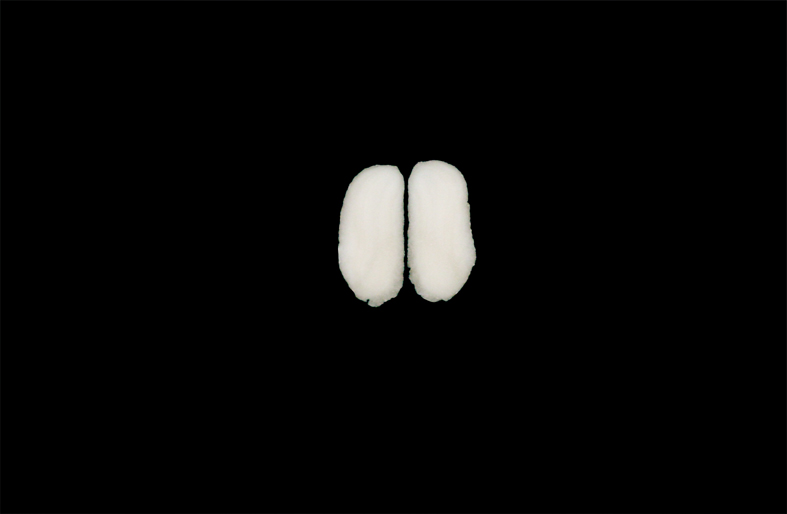

Supplement: Supplementary file 2 — Dataset 1 [file 41598_2019_55585_MOESM2_ESM.zip › 017.jpg]

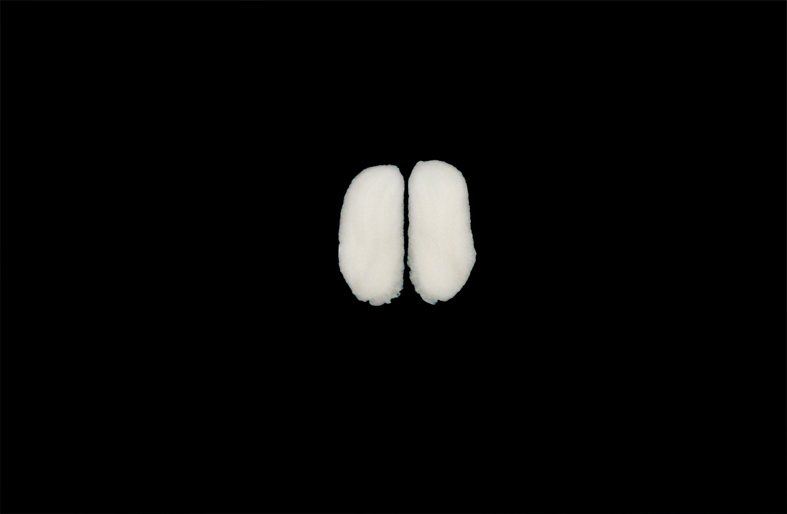

Supplement: Supplementary file 2 — Dataset 1 [file 41598_2019_55585_MOESM2_ESM.zip › 018.jpg]

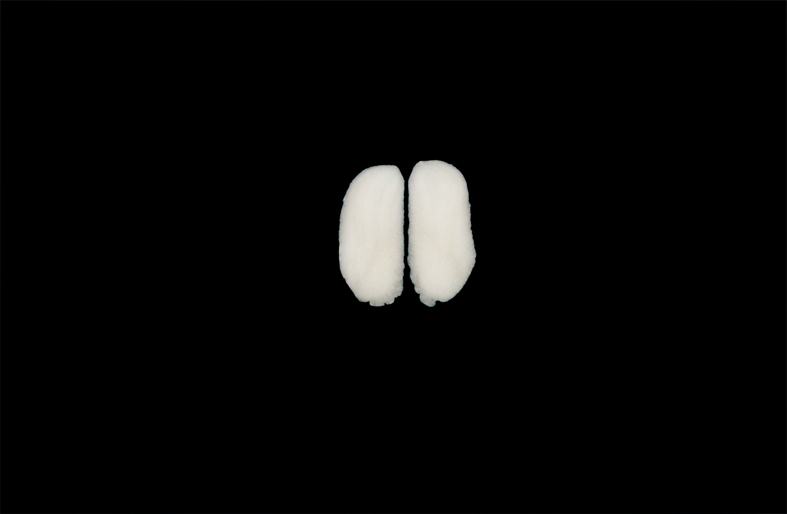

Supplement: Supplementary file 2 — Dataset 1 [file 41598_2019_55585_MOESM2_ESM.zip › 019.jpg]

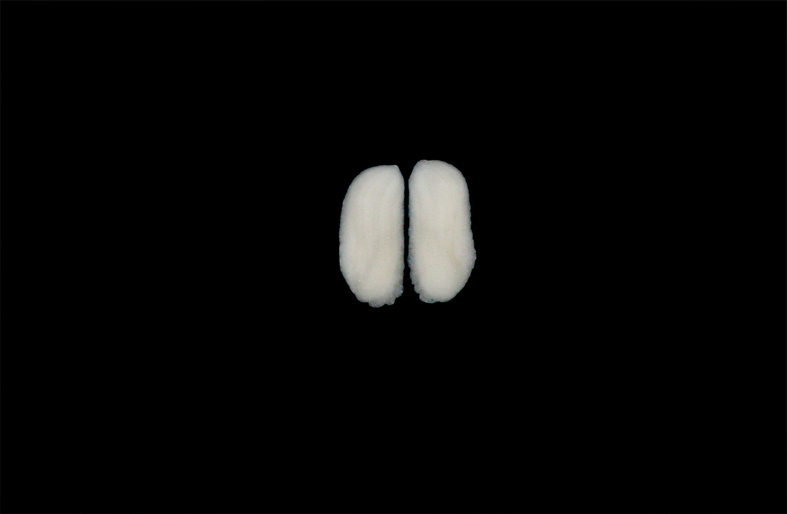

Supplement: Supplementary file 2 — Dataset 1 [file 41598_2019_55585_MOESM2_ESM.zip › 020.jpg]

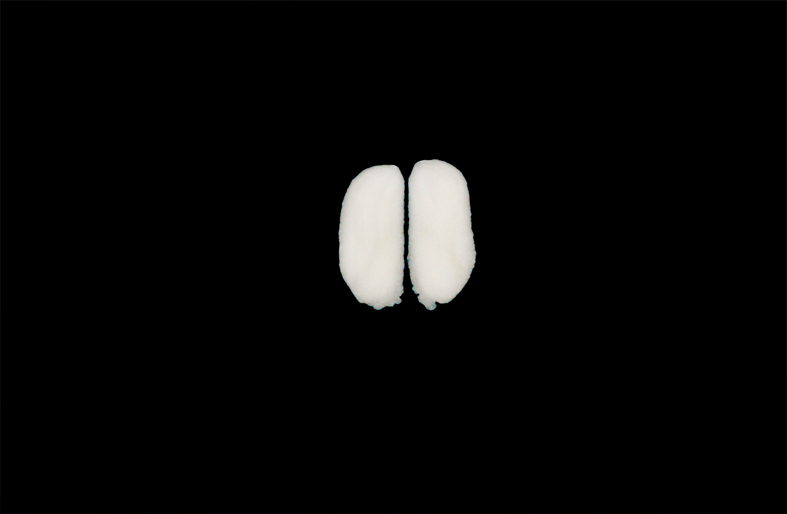

Supplement: Supplementary file 2 — Dataset 1 [file 41598_2019_55585_MOESM2_ESM.zip › 021.jpg]

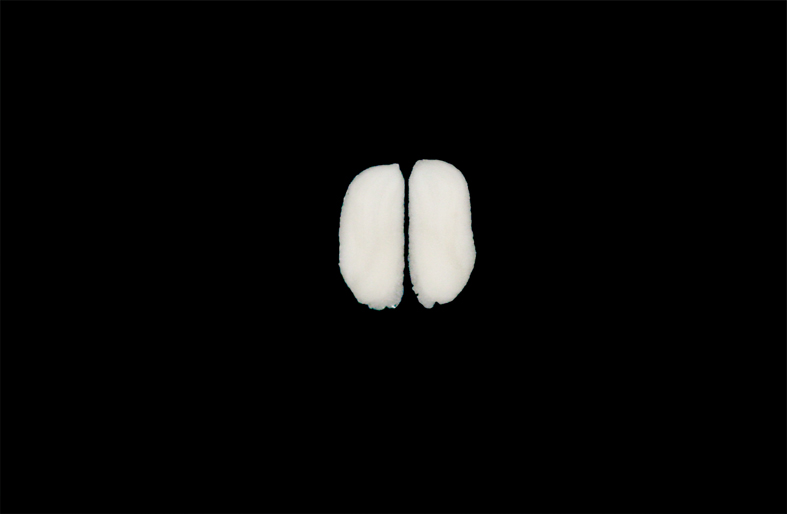

Supplement: Supplementary file 2 — Dataset 1 [file 41598_2019_55585_MOESM2_ESM.zip › 022.jpg]

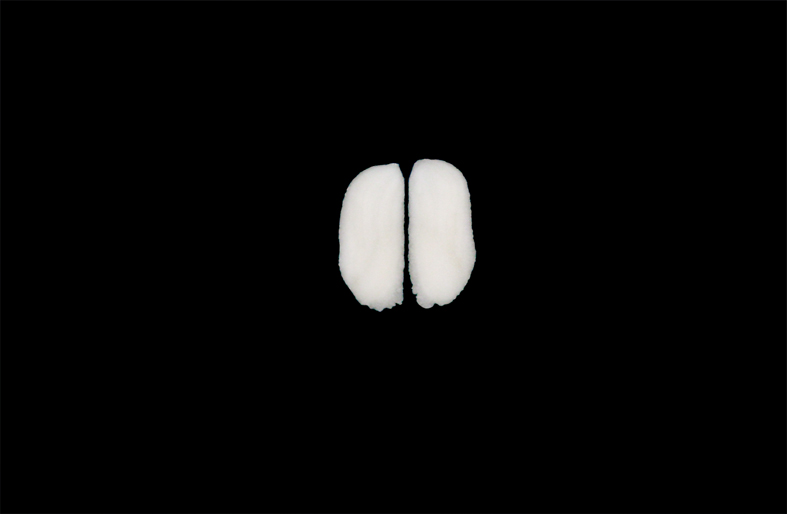

Supplement: Supplementary file 2 — Dataset 1 [file 41598_2019_55585_MOESM2_ESM.zip › 023.jpg]

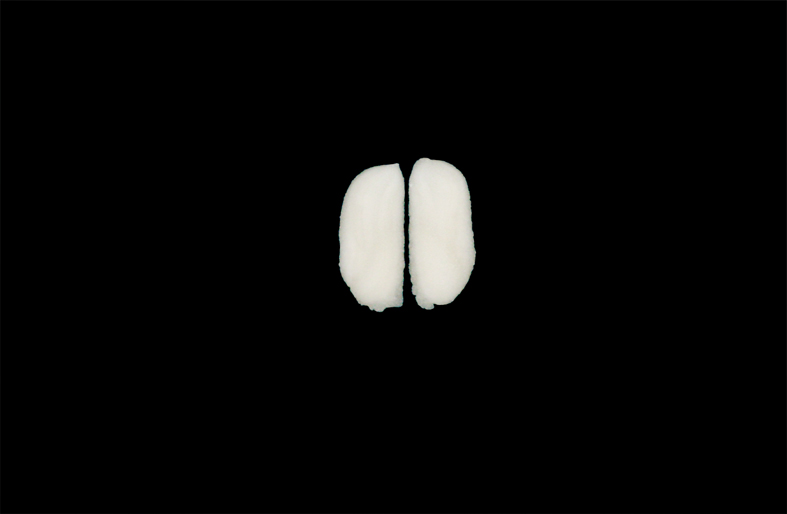

Supplement: Supplementary file 2 — Dataset 1 [file 41598_2019_55585_MOESM2_ESM.zip › 024.jpg]

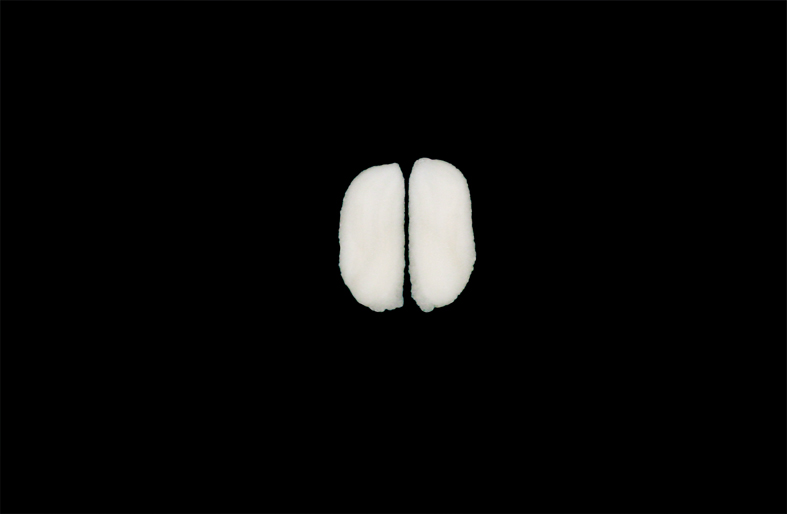

Supplement: Supplementary file 2 — Dataset 1 [file 41598_2019_55585_MOESM2_ESM.zip › 025.jpg]

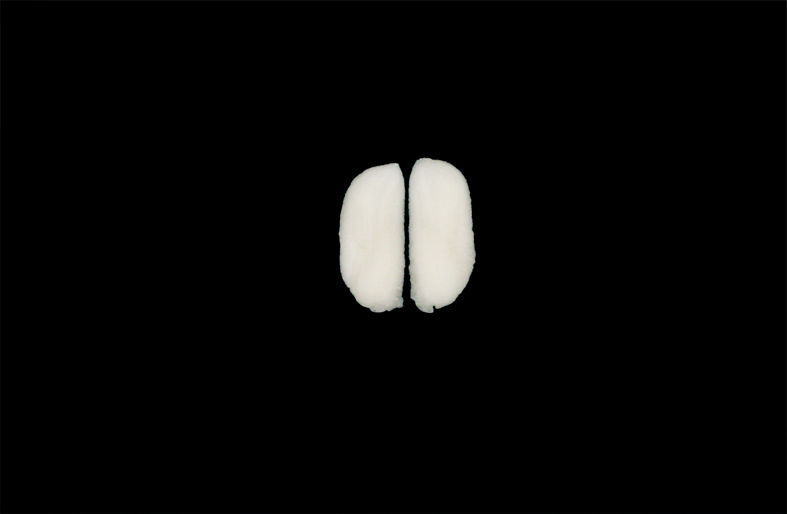

Supplement: Supplementary file 2 — Dataset 1 [file 41598_2019_55585_MOESM2_ESM.zip › 026.jpg]

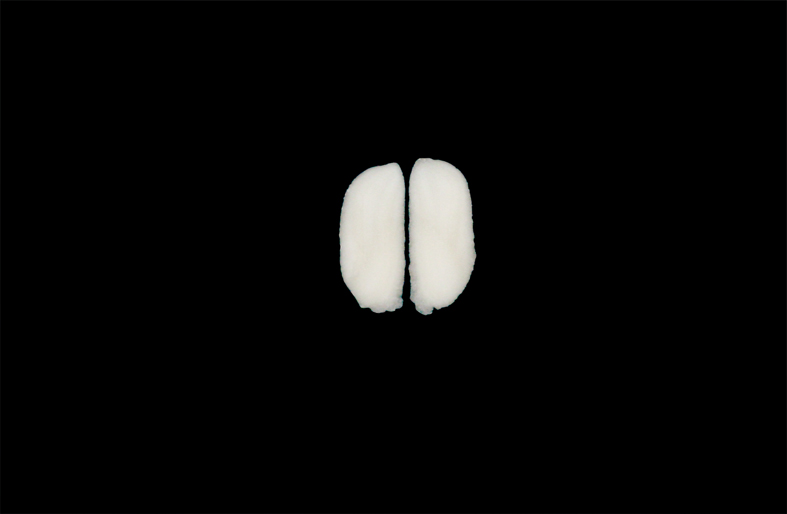

Supplement: Supplementary file 2 — Dataset 1 [file 41598_2019_55585_MOESM2_ESM.zip › 027.jpg]

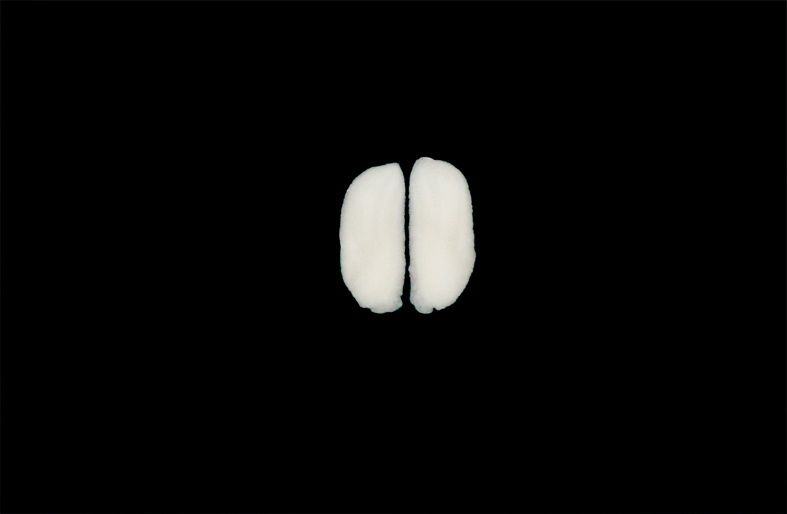

Supplement: Supplementary file 2 — Dataset 1 [file 41598_2019_55585_MOESM2_ESM.zip › 028.jpg]

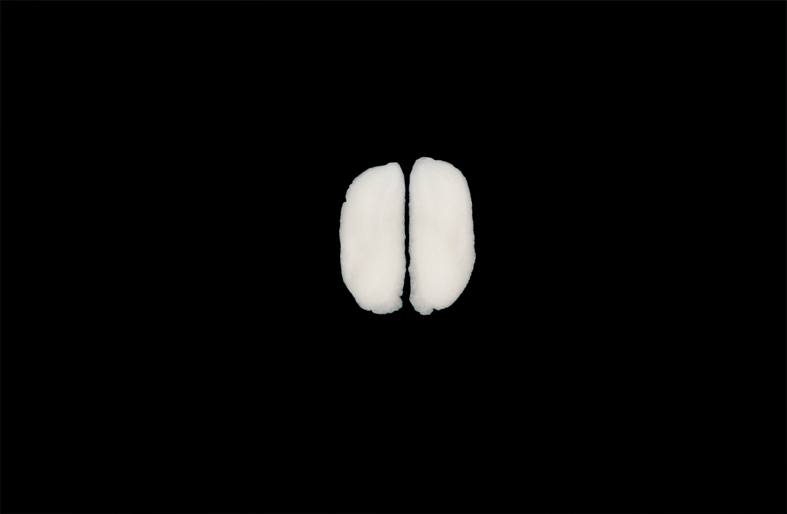

Supplement: Supplementary file 2 — Dataset 1 [file 41598_2019_55585_MOESM2_ESM.zip › 029.jpg]

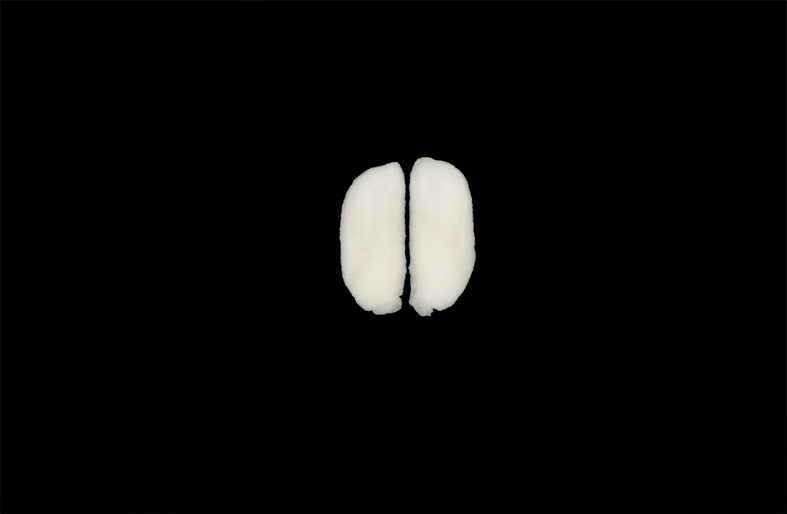

Supplement: Supplementary file 2 — Dataset 1 [file 41598_2019_55585_MOESM2_ESM.zip › 030.jpg]

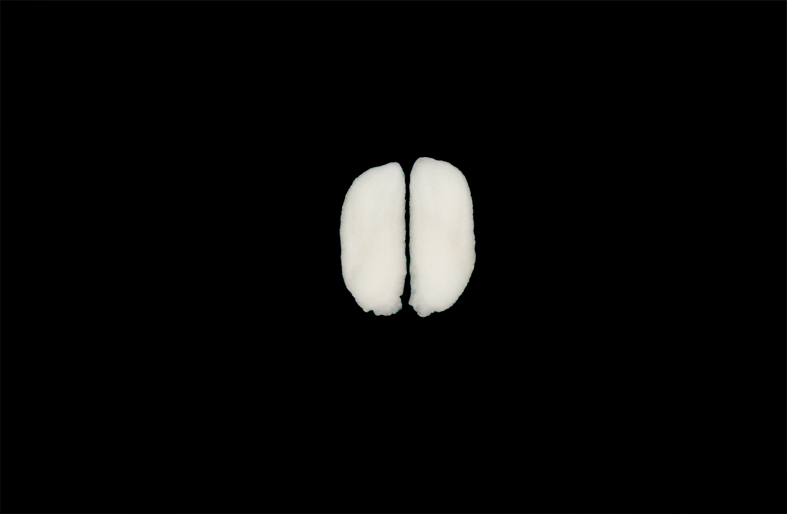

Supplement: Supplementary file 2 — Dataset 1 [file 41598_2019_55585_MOESM2_ESM.zip › 031.jpg]

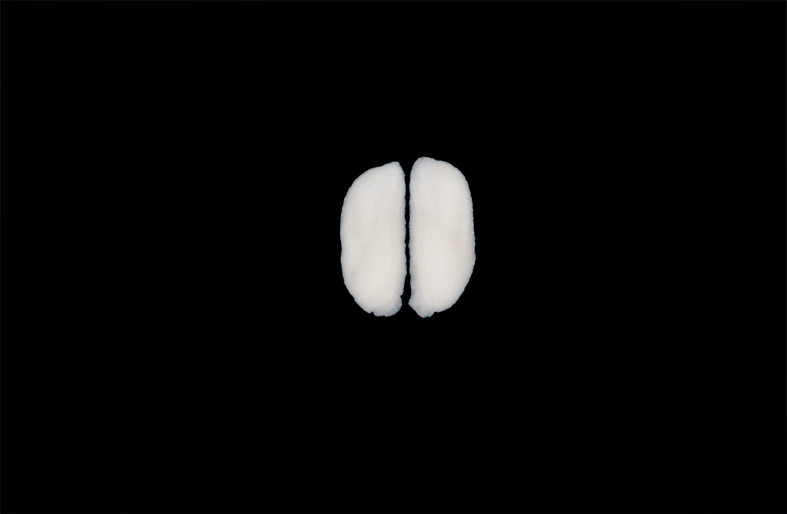

Supplement: Supplementary file 2 — Dataset 1 [file 41598_2019_55585_MOESM2_ESM.zip › 032.jpg]

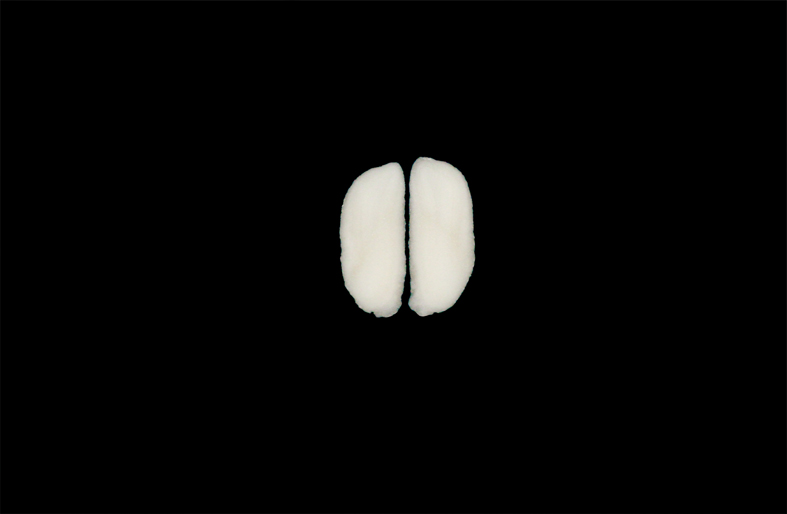

Supplement: Supplementary file 2 — Dataset 1 [file 41598_2019_55585_MOESM2_ESM.zip › 033.jpg]

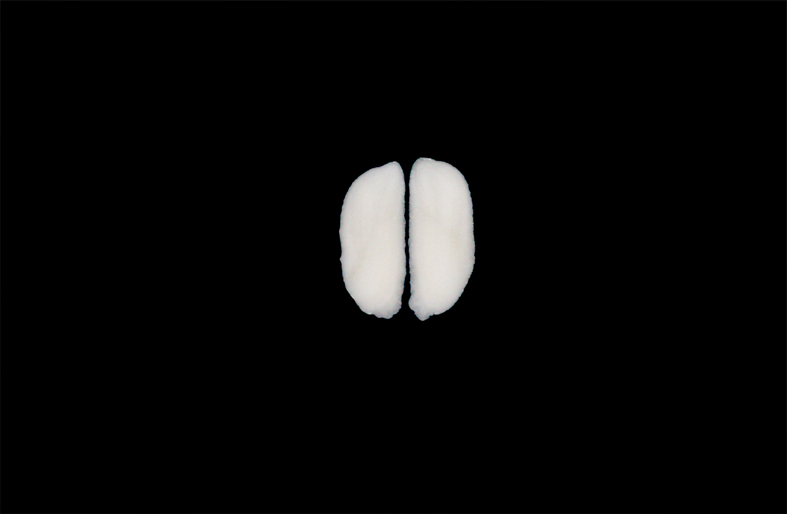

Supplement: Supplementary file 2 — Dataset 1 [file 41598_2019_55585_MOESM2_ESM.zip › 034.jpg]

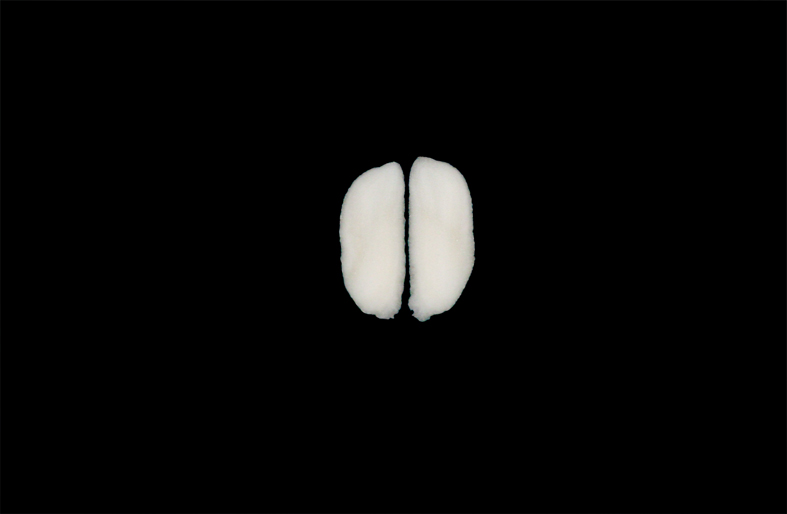

Supplement: Supplementary file 2 — Dataset 1 [file 41598_2019_55585_MOESM2_ESM.zip › 035.jpg]

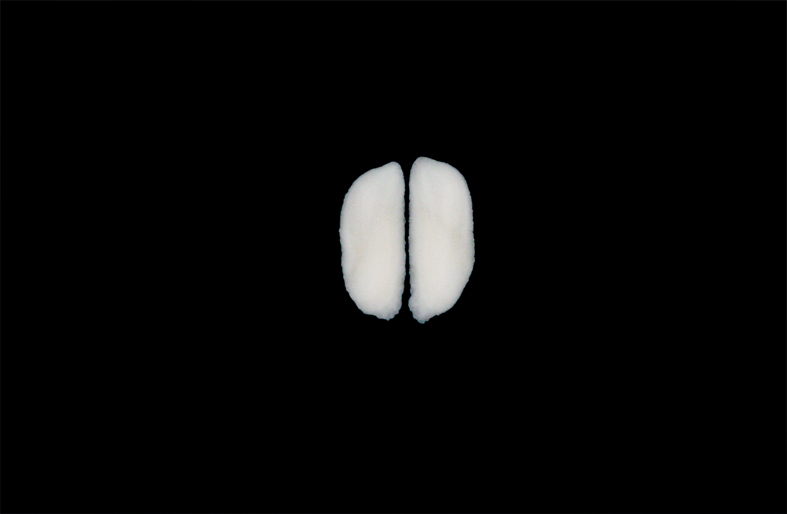

Supplement: Supplementary file 2 — Dataset 1 [file 41598_2019_55585_MOESM2_ESM.zip › 036.jpg]

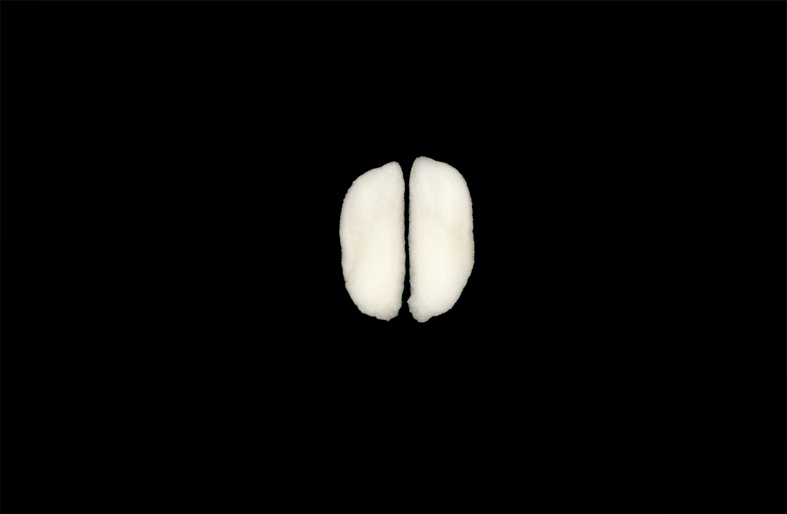

Supplement: Supplementary file 2 — Dataset 1 [file 41598_2019_55585_MOESM2_ESM.zip › 037.jpg]

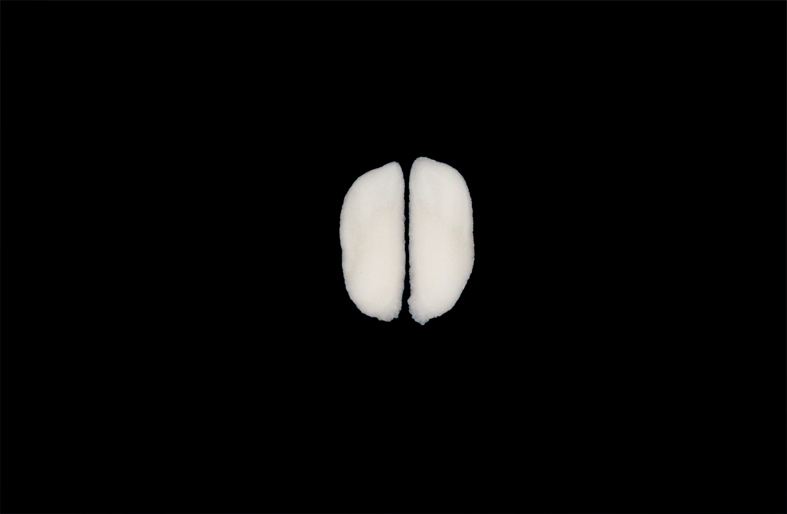

Supplement: Supplementary file 2 — Dataset 1 [file 41598_2019_55585_MOESM2_ESM.zip › 038.jpg]

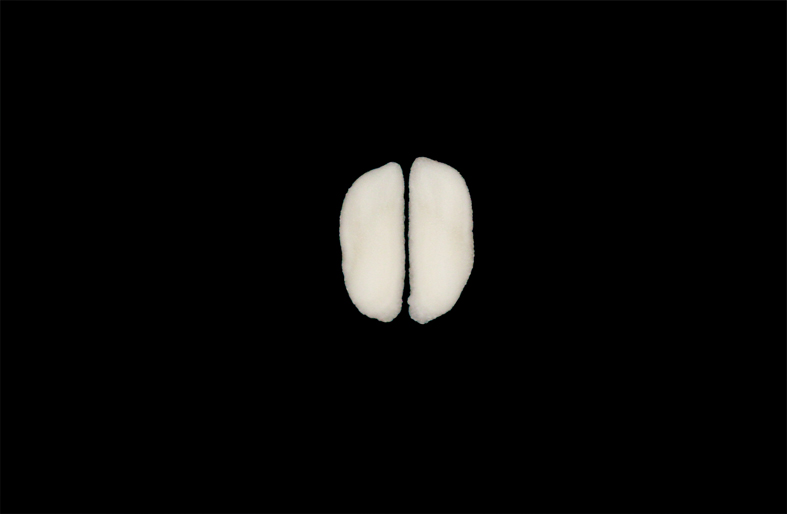

Supplement: Supplementary file 2 — Dataset 1 [file 41598_2019_55585_MOESM2_ESM.zip › 039.jpg]

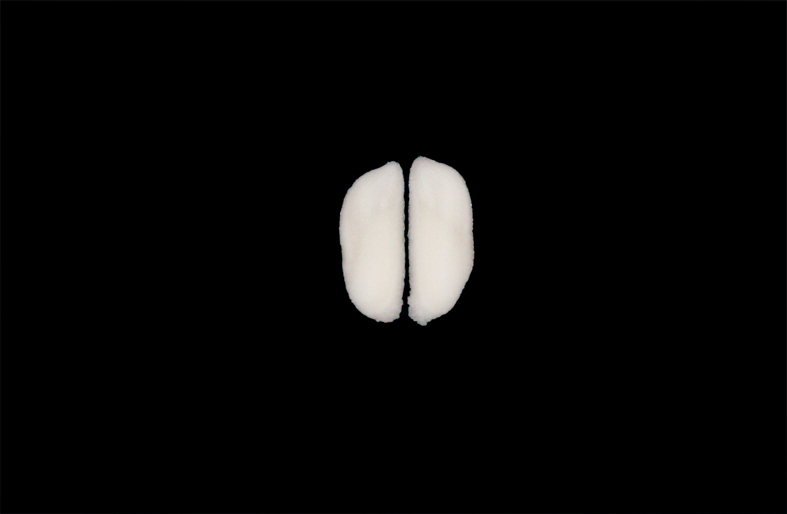

Supplement: Supplementary file 2 — Dataset 1 [file 41598_2019_55585_MOESM2_ESM.zip › 040.jpg]

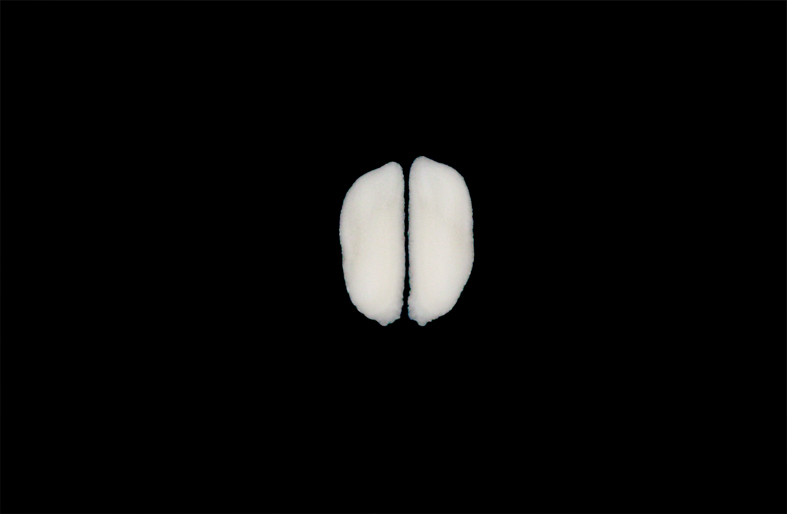

Supplement: Supplementary file 2 — Dataset 1 [file 41598_2019_55585_MOESM2_ESM.zip › 041.jpg]

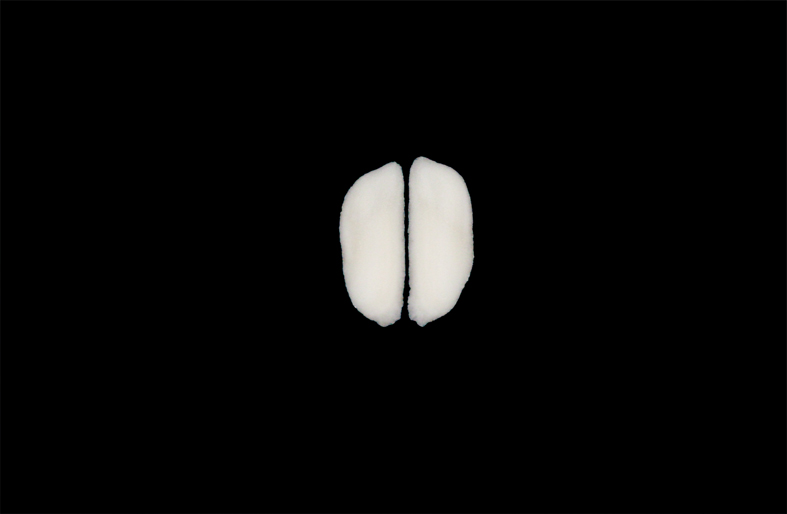

Supplement: Supplementary file 2 — Dataset 1 [file 41598_2019_55585_MOESM2_ESM.zip › 042.jpg]

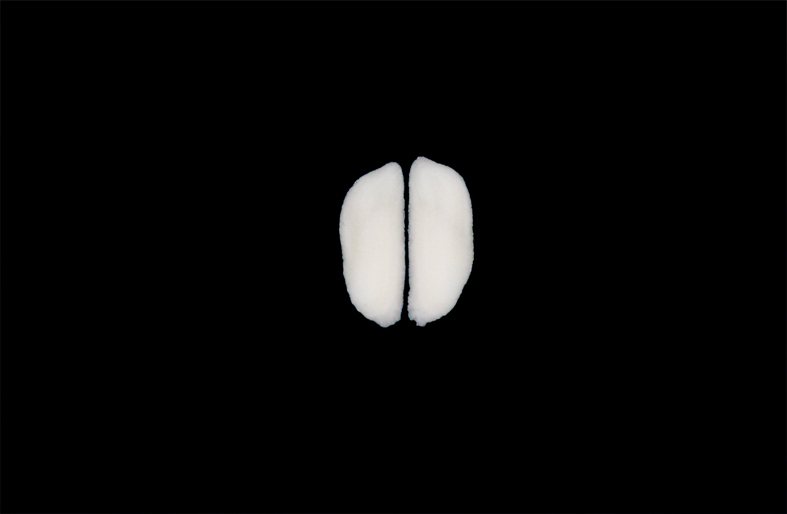

Supplement: Supplementary file 2 — Dataset 1 [file 41598_2019_55585_MOESM2_ESM.zip › 043.jpg]

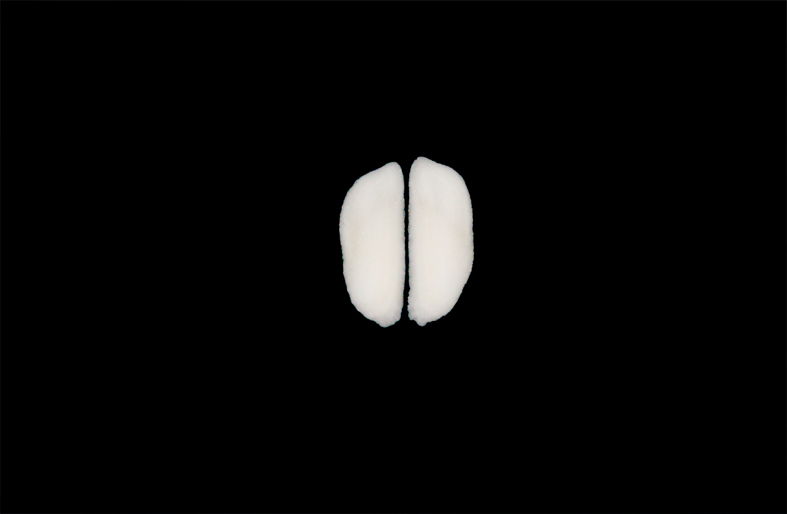

Supplement: Supplementary file 2 — Dataset 1 [file 41598_2019_55585_MOESM2_ESM.zip › 044.jpg]

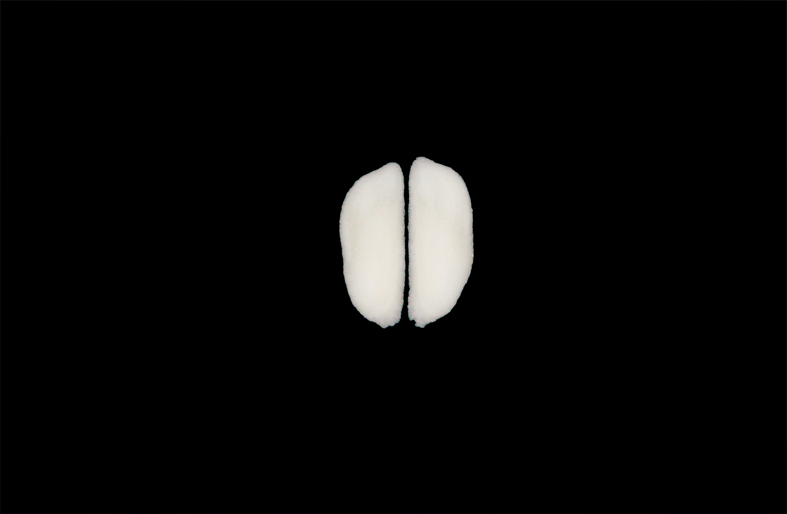

Supplement: Supplementary file 2 — Dataset 1 [file 41598_2019_55585_MOESM2_ESM.zip › 045.jpg]

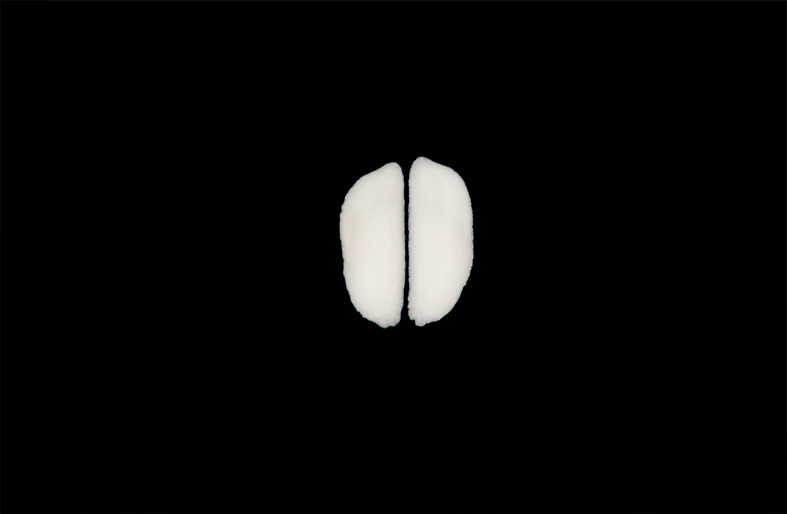

Supplement: Supplementary file 2 — Dataset 1 [file 41598_2019_55585_MOESM2_ESM.zip › 046.jpg]

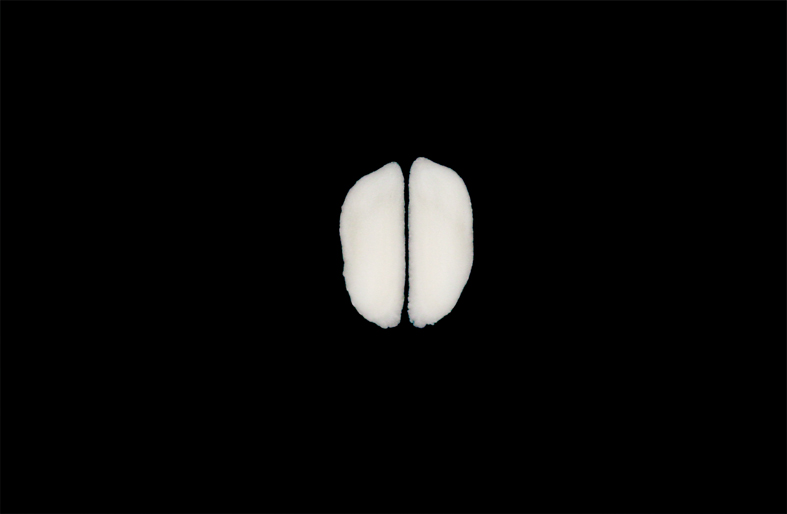

Supplement: Supplementary file 2 — Dataset 1 [file 41598_2019_55585_MOESM2_ESM.zip › 047.jpg]

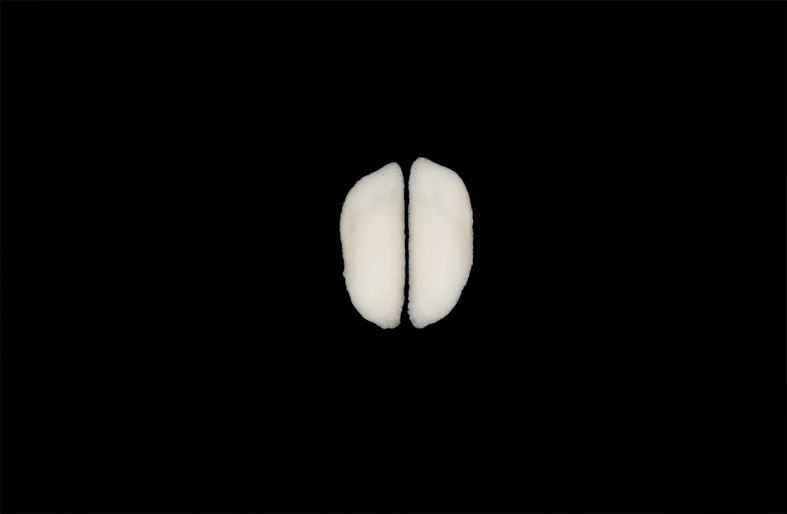

Supplement: Supplementary file 2 — Dataset 1 [file 41598_2019_55585_MOESM2_ESM.zip › 048.jpg]

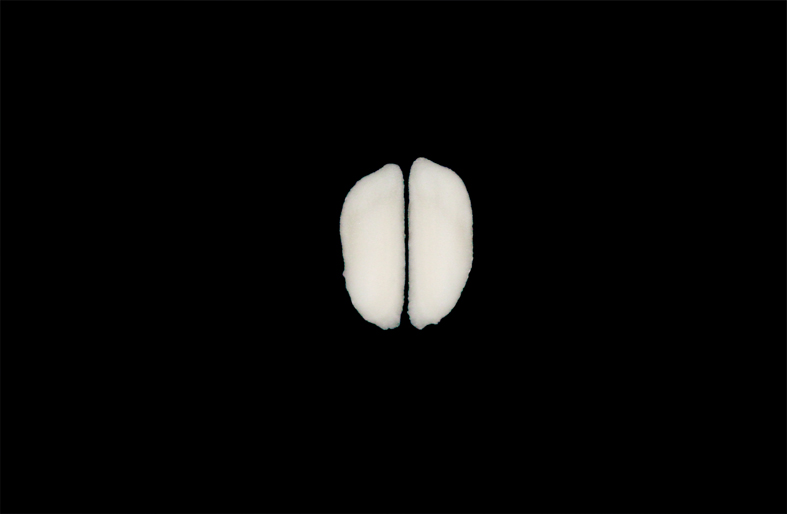

Supplement: Supplementary file 2 — Dataset 1 [file 41598_2019_55585_MOESM2_ESM.zip › 049.jpg]

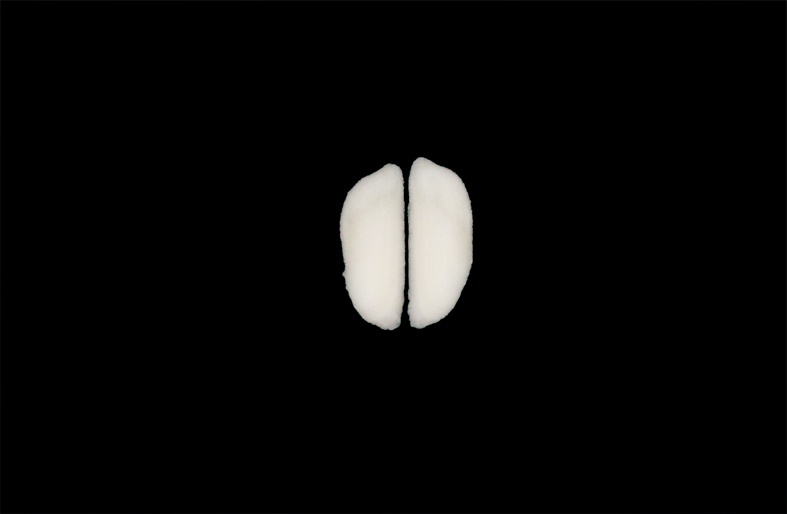

Supplement: Supplementary file 2 — Dataset 1 [file 41598_2019_55585_MOESM2_ESM.zip › 050.jpg]

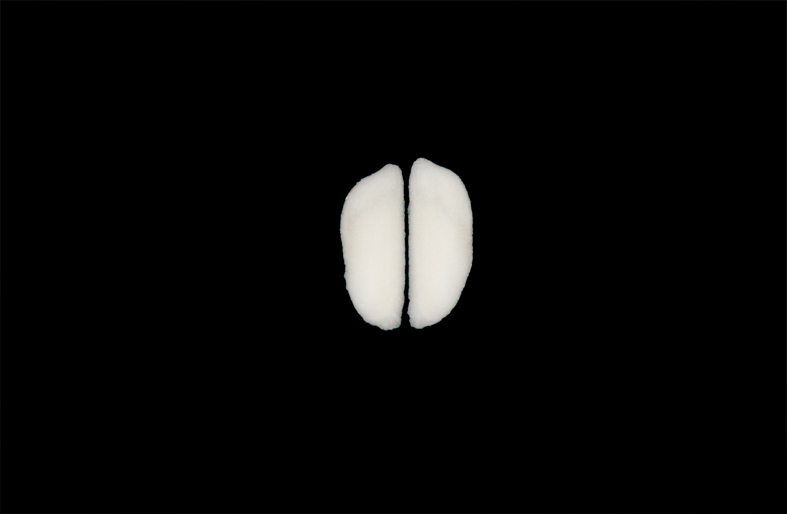

Supplement: Supplementary file 2 — Dataset 1 [file 41598_2019_55585_MOESM2_ESM.zip › 051.jpg]

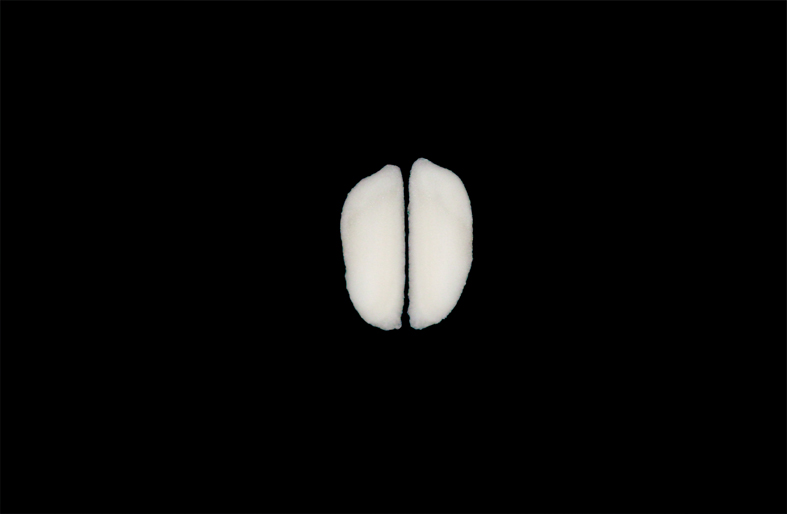

Supplement: Supplementary file 2 — Dataset 1 [file 41598_2019_55585_MOESM2_ESM.zip › 052.jpg]

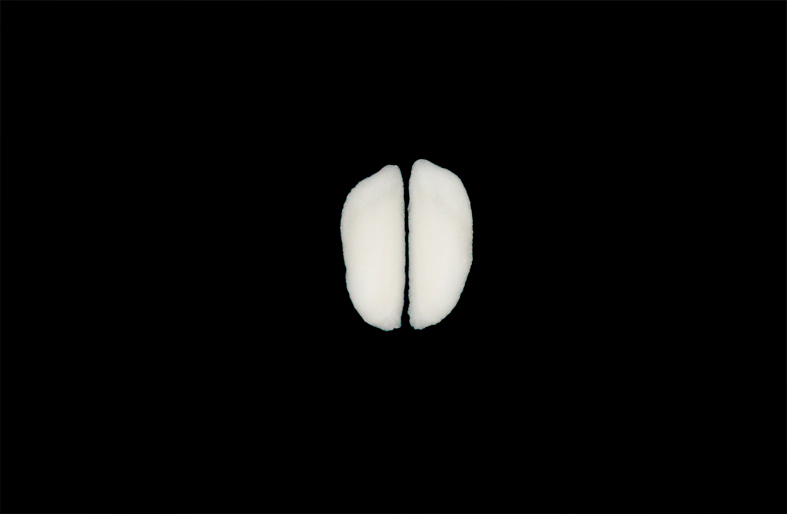

Supplement: Supplementary file 2 — Dataset 1 [file 41598_2019_55585_MOESM2_ESM.zip › 053.jpg]

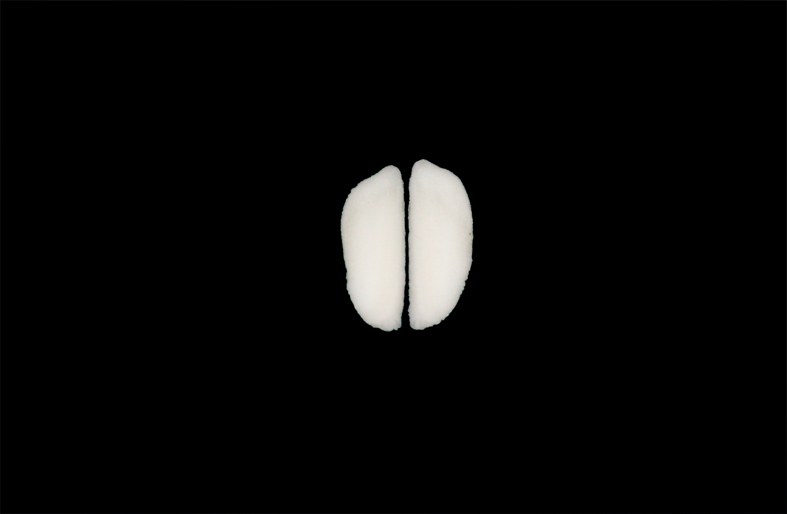

Supplement: Supplementary file 2 — Dataset 1 [file 41598_2019_55585_MOESM2_ESM.zip › 054.jpg]

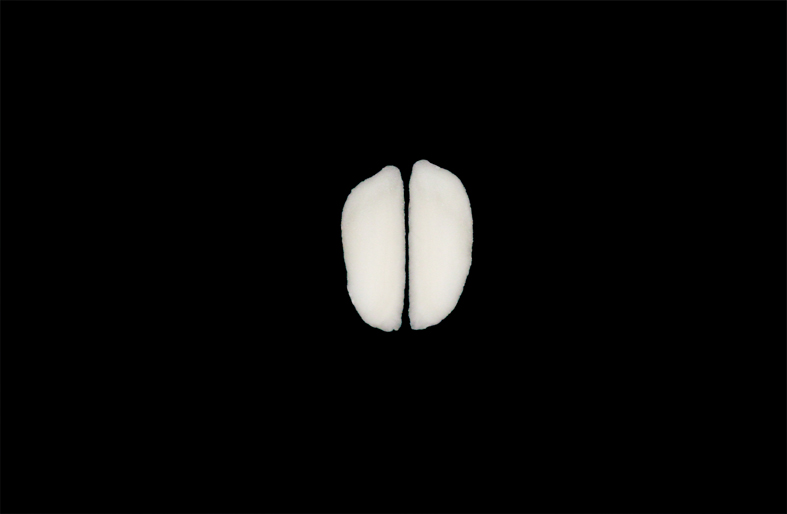

Supplement: Supplementary file 2 — Dataset 1 [file 41598_2019_55585_MOESM2_ESM.zip › 055.jpg]

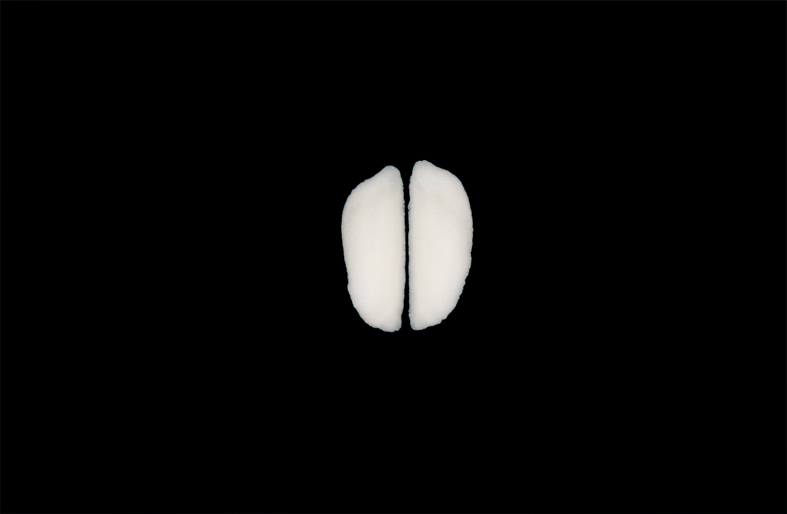

Supplement: Supplementary file 2 — Dataset 1 [file 41598_2019_55585_MOESM2_ESM.zip › 056.jpg]

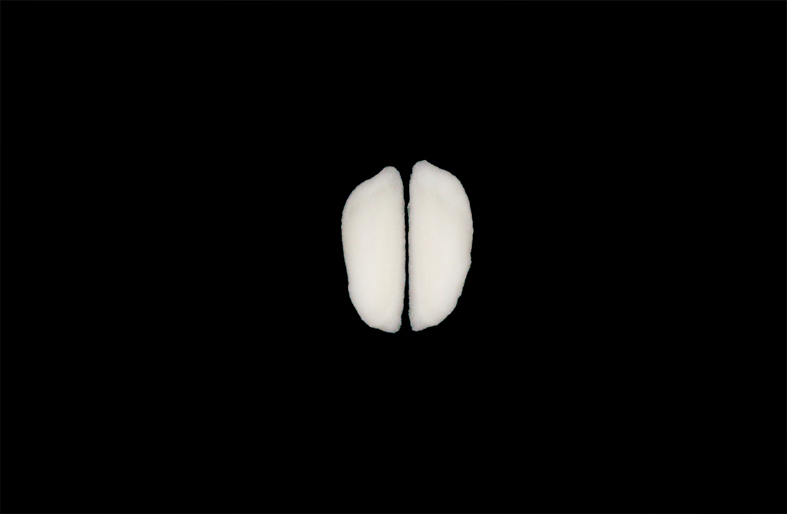

Supplement: Supplementary file 2 — Dataset 1 [file 41598_2019_55585_MOESM2_ESM.zip › 057.jpg]

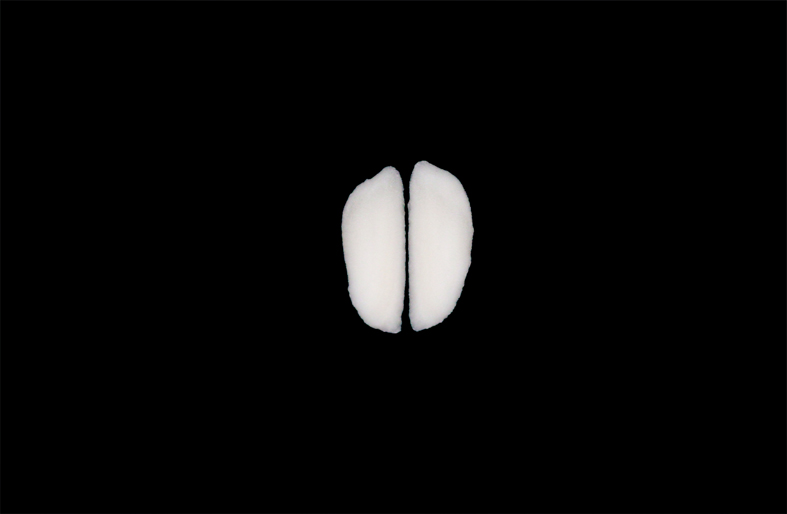

Supplement: Supplementary file 2 — Dataset 1 [file 41598_2019_55585_MOESM2_ESM.zip › 058.jpg]

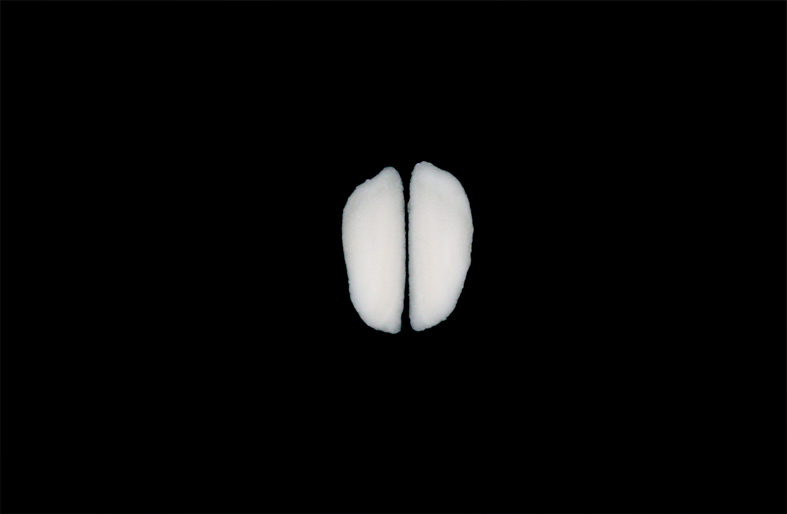

Supplement: Supplementary file 2 — Dataset 1 [file 41598_2019_55585_MOESM2_ESM.zip › 059.jpg]

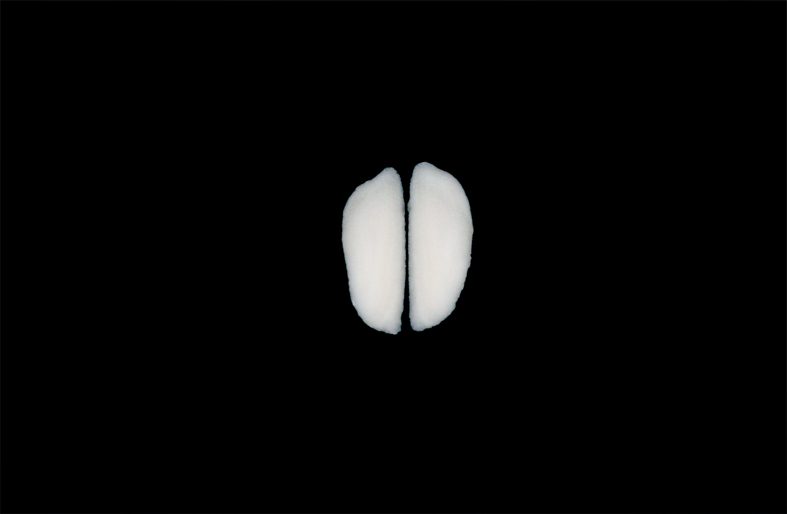

Supplement: Supplementary file 2 — Dataset 1 [file 41598_2019_55585_MOESM2_ESM.zip › 060.jpg]

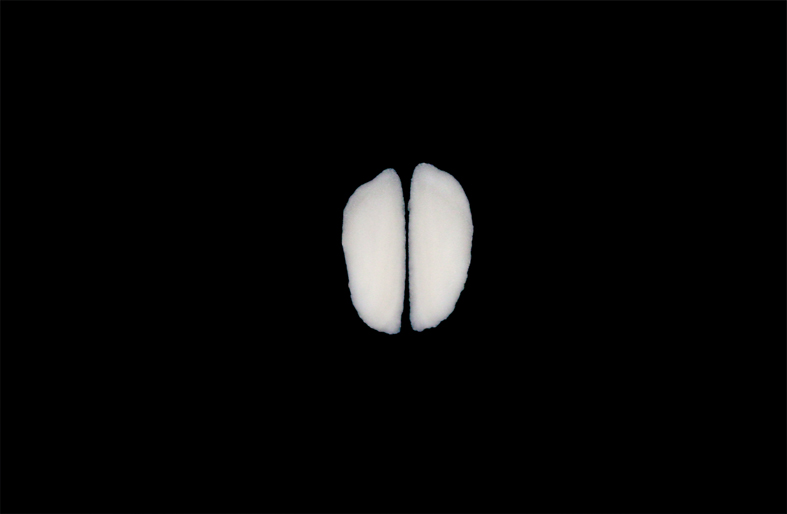

Supplement: Supplementary file 2 — Dataset 1 [file 41598_2019_55585_MOESM2_ESM.zip › 061.jpg]

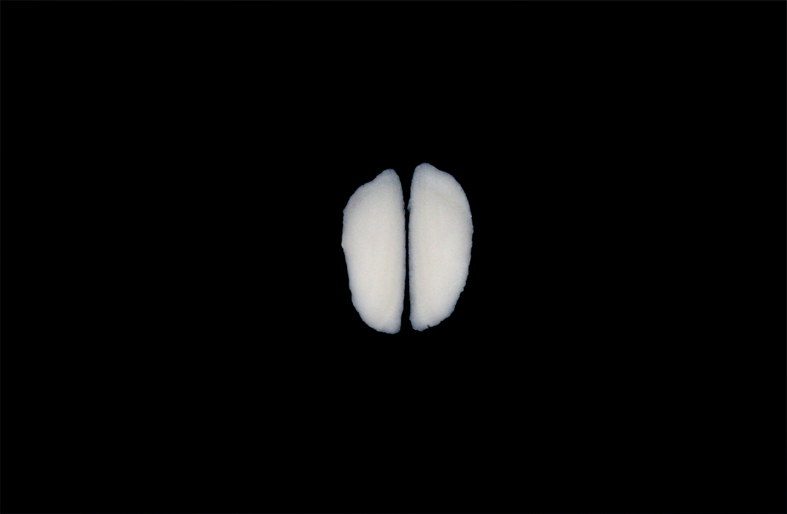

Supplement: Supplementary file 2 — Dataset 1 [file 41598_2019_55585_MOESM2_ESM.zip › 062.jpg]

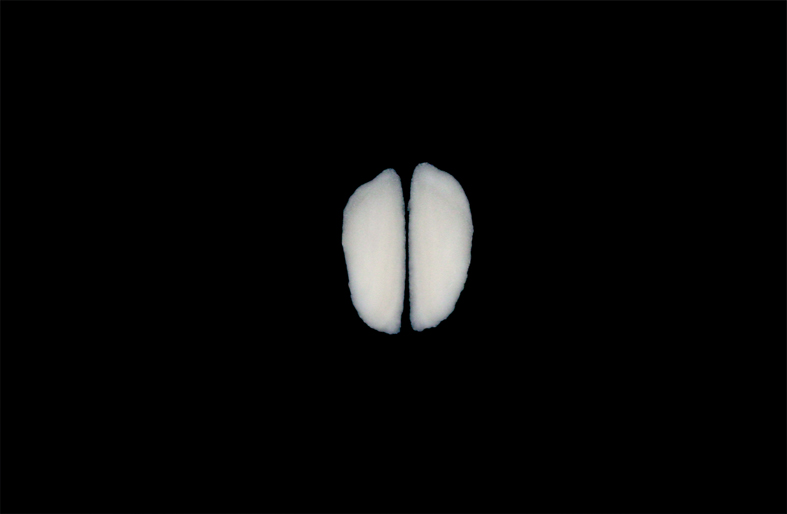

Supplement: Supplementary file 2 — Dataset 1 [file 41598_2019_55585_MOESM2_ESM.zip › 063.jpg]

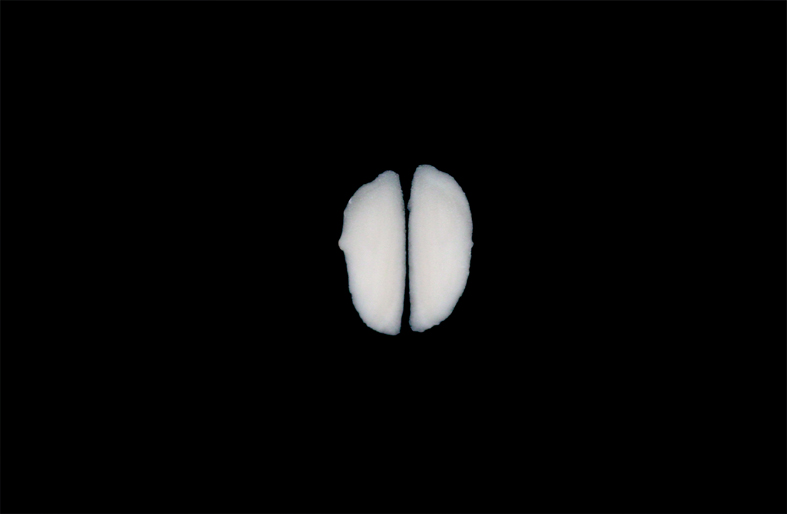

Supplement: Supplementary file 2 — Dataset 1 [file 41598_2019_55585_MOESM2_ESM.zip › 064.jpg]

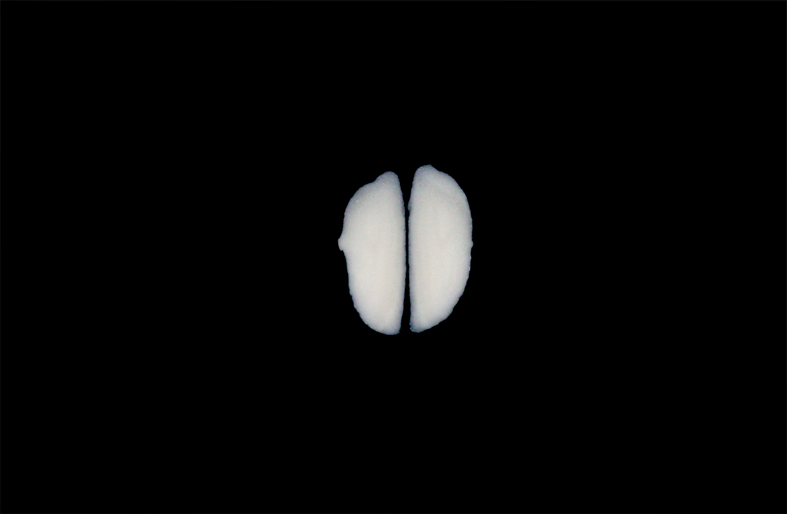

Supplement: Supplementary file 2 — Dataset 1 [file 41598_2019_55585_MOESM2_ESM.zip › 065.jpg]

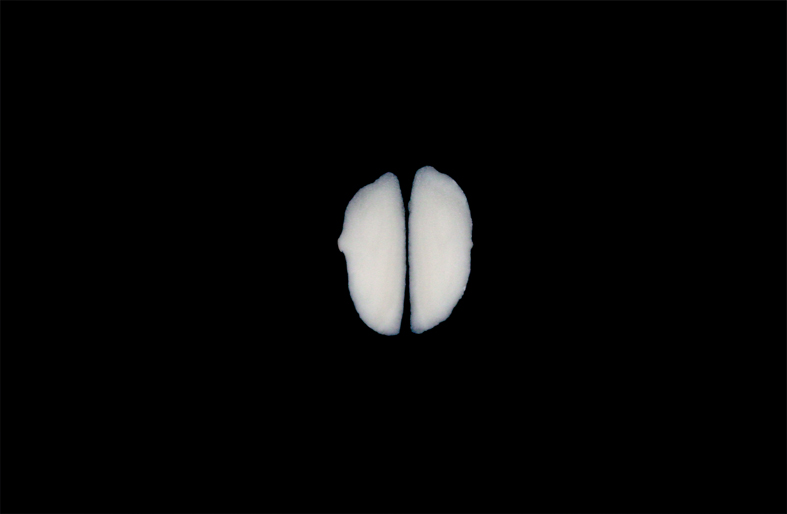

Supplement: Supplementary file 2 — Dataset 1 [file 41598_2019_55585_MOESM2_ESM.zip › 066.jpg]

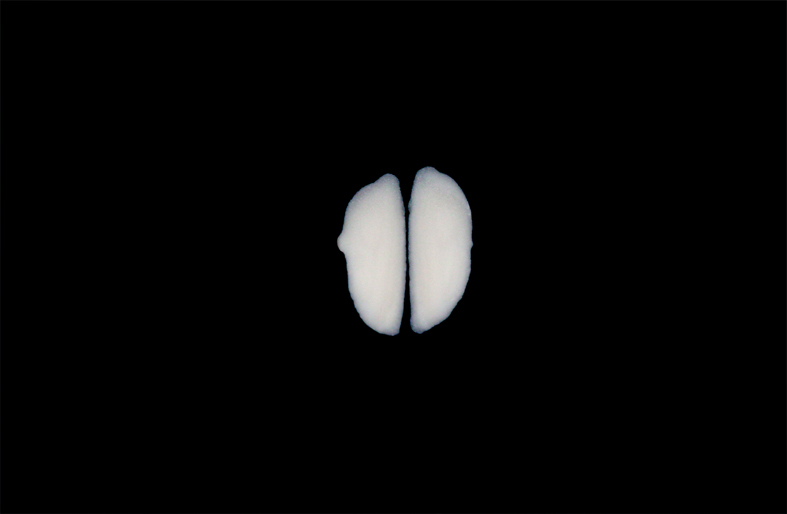

Supplement: Supplementary file 2 — Dataset 1 [file 41598_2019_55585_MOESM2_ESM.zip › 067.jpg]

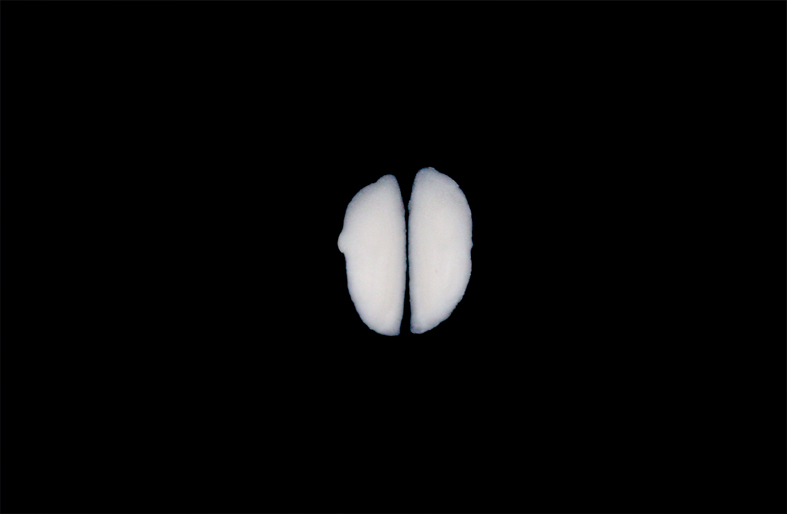

Supplement: Supplementary file 2 — Dataset 1 [file 41598_2019_55585_MOESM2_ESM.zip › 068.jpg]

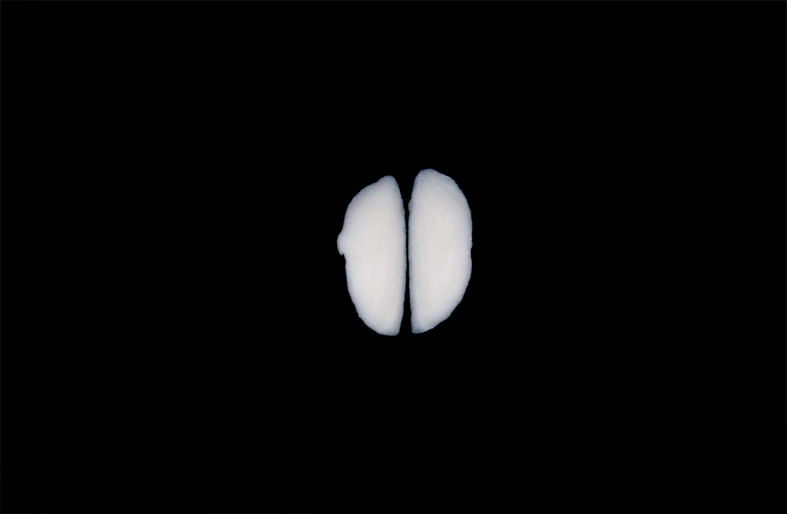

Supplement: Supplementary file 2 — Dataset 1 [file 41598_2019_55585_MOESM2_ESM.zip › 069.jpg]

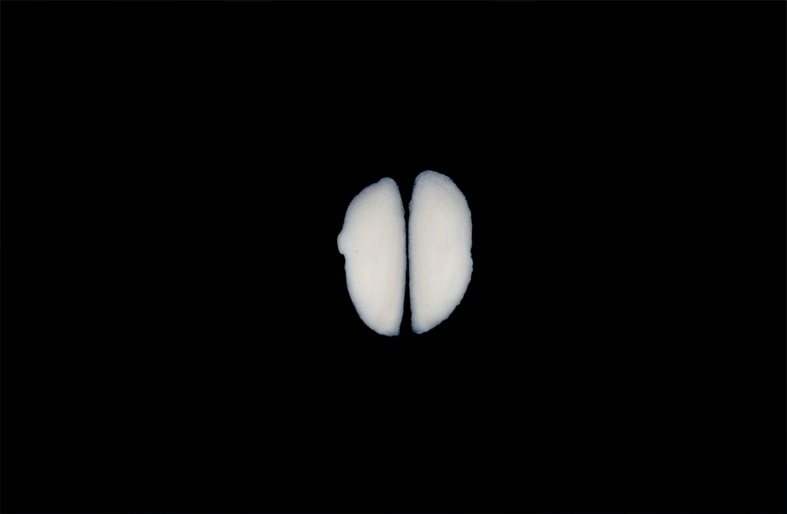

Supplement: Supplementary file 2 — Dataset 1 [file 41598_2019_55585_MOESM2_ESM.zip › 070.jpg]

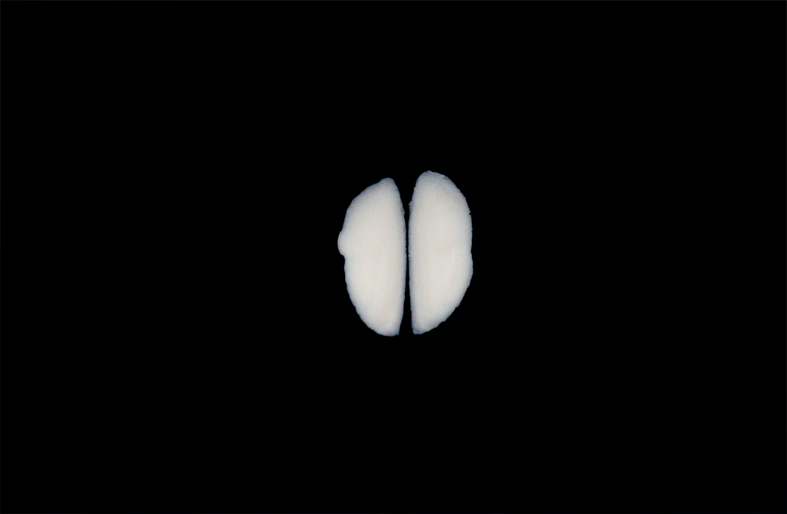

Supplement: Supplementary file 2 — Dataset 1 [file 41598_2019_55585_MOESM2_ESM.zip › 071.jpg]

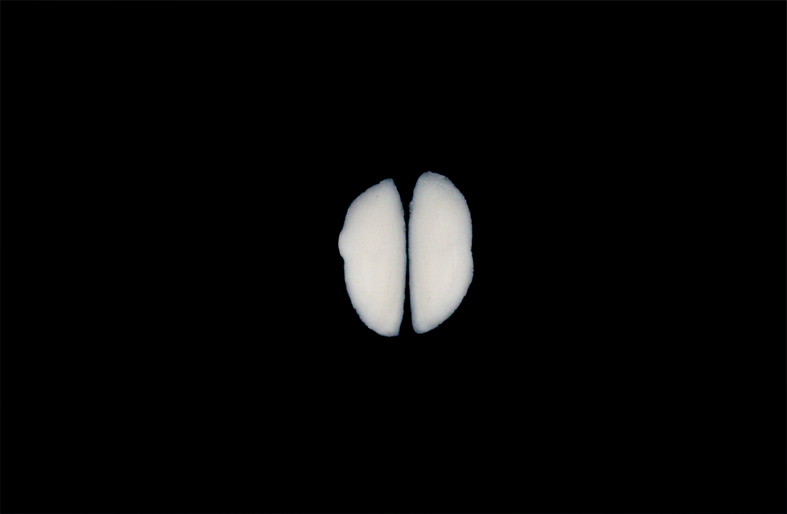

Supplement: Supplementary file 2 — Dataset 1 [file 41598_2019_55585_MOESM2_ESM.zip › 072.jpg]

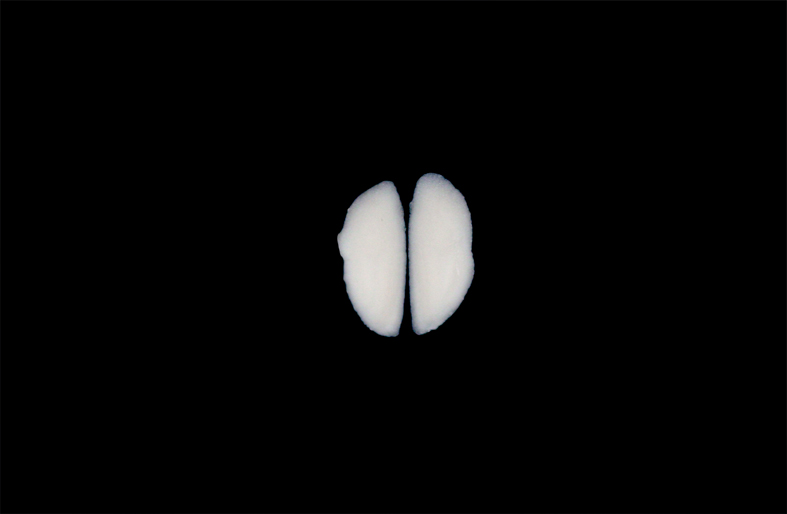

Supplement: Supplementary file 2 — Dataset 1 [file 41598_2019_55585_MOESM2_ESM.zip › 073.jpg]

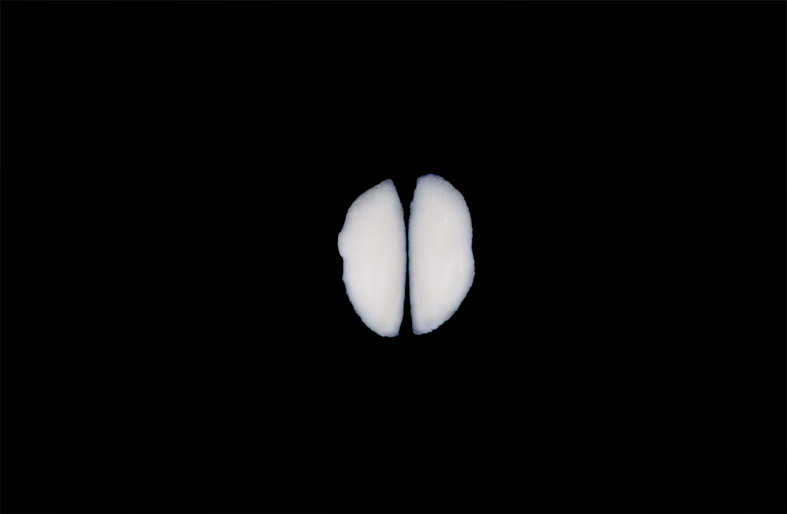

Supplement: Supplementary file 2 — Dataset 1 [file 41598_2019_55585_MOESM2_ESM.zip › 074.jpg]

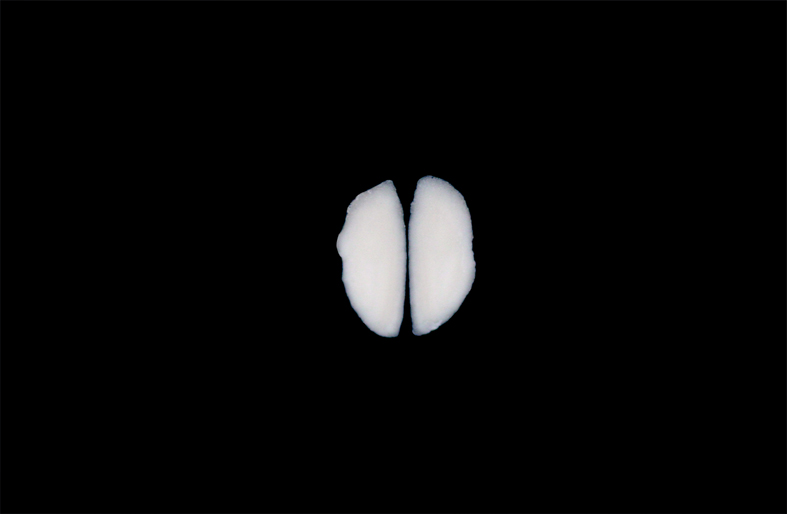

Supplement: Supplementary file 2 — Dataset 1 [file 41598_2019_55585_MOESM2_ESM.zip › 075.jpg]

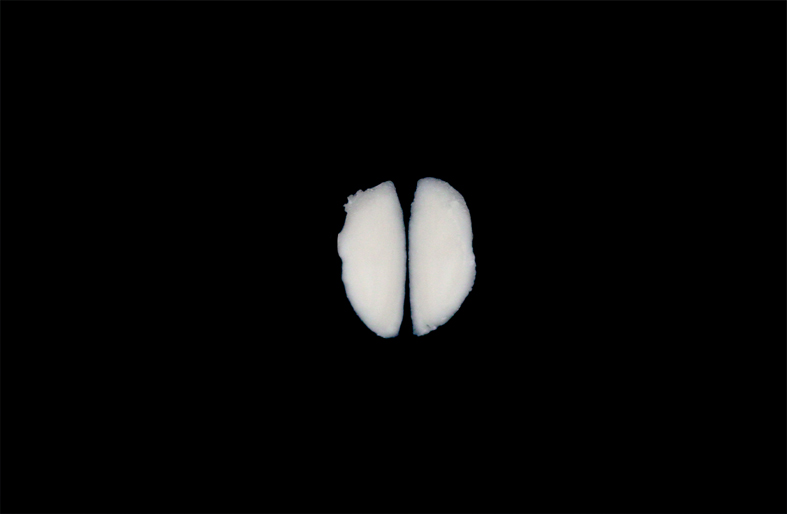

Supplement: Supplementary file 2 — Dataset 1 [file 41598_2019_55585_MOESM2_ESM.zip › 076.jpg]

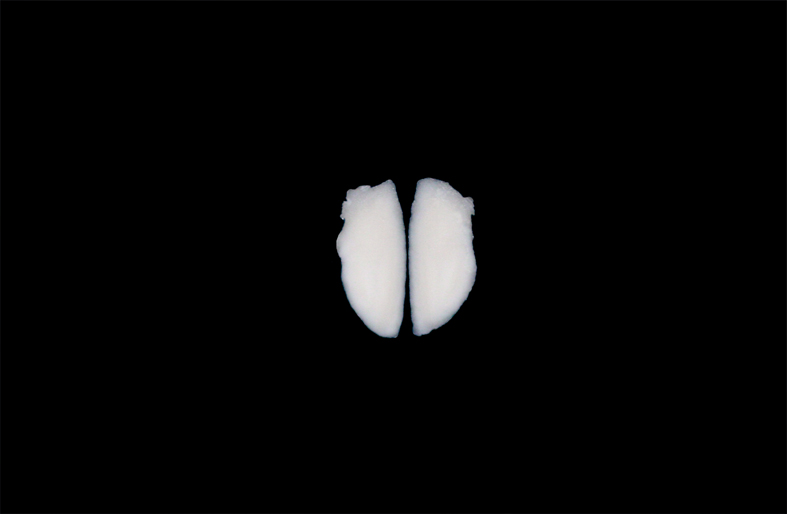

Supplement: Supplementary file 2 — Dataset 1 [file 41598_2019_55585_MOESM2_ESM.zip › 077.jpg]

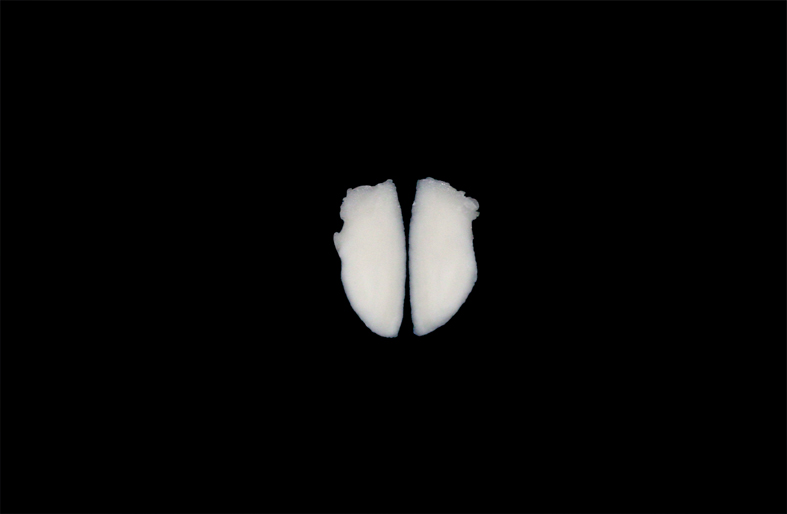

Supplement: Supplementary file 2 — Dataset 1 [file 41598_2019_55585_MOESM2_ESM.zip › 078.jpg]

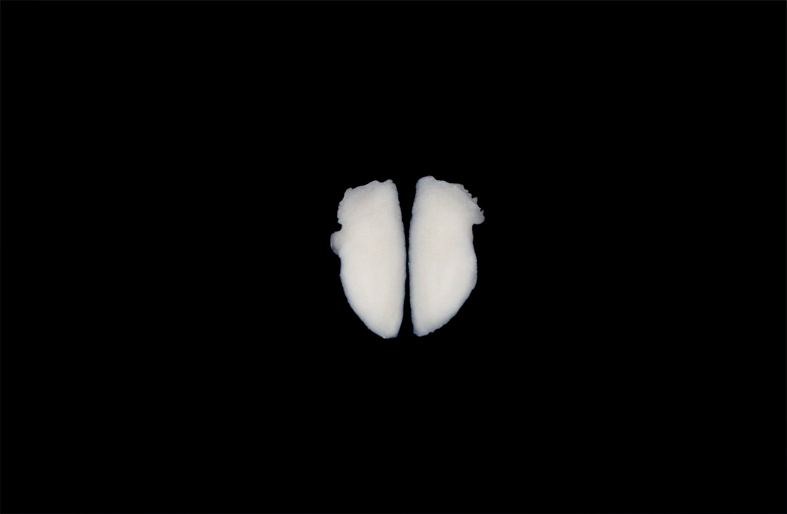

Supplement: Supplementary file 2 — Dataset 1 [file 41598_2019_55585_MOESM2_ESM.zip › 079.jpg]

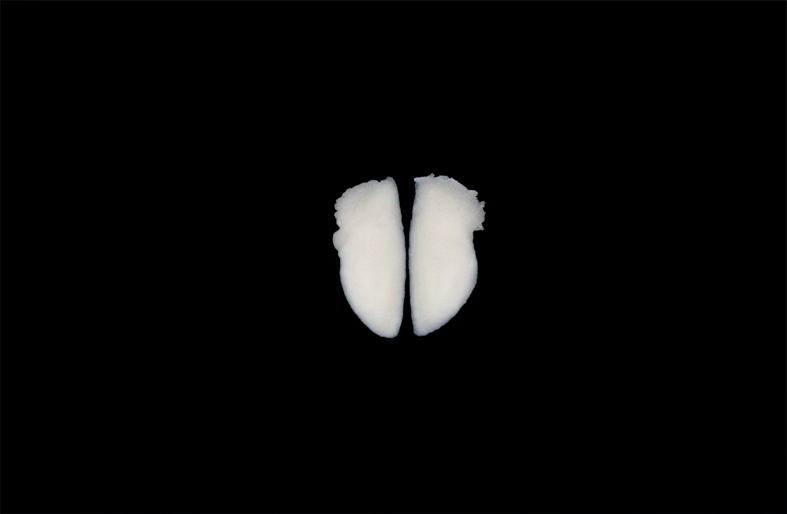

Supplement: Supplementary file 2 — Dataset 1 [file 41598_2019_55585_MOESM2_ESM.zip › 080.jpg]

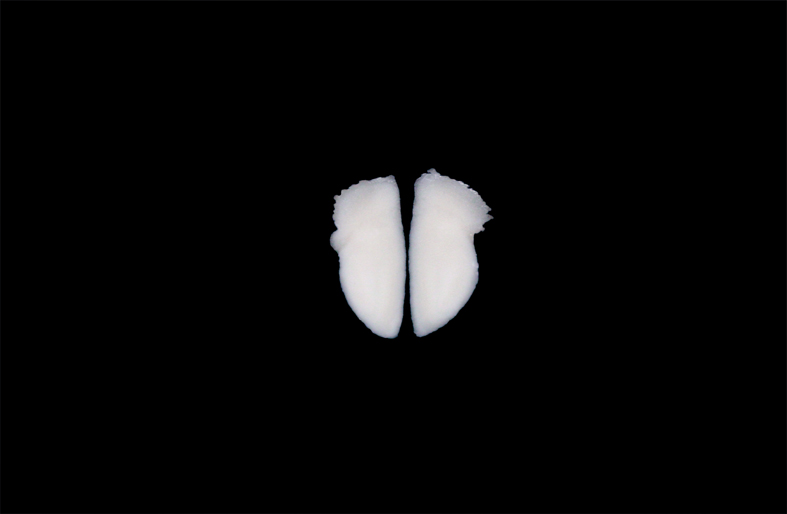

Supplement: Supplementary file 2 — Dataset 1 [file 41598_2019_55585_MOESM2_ESM.zip › 081.jpg]

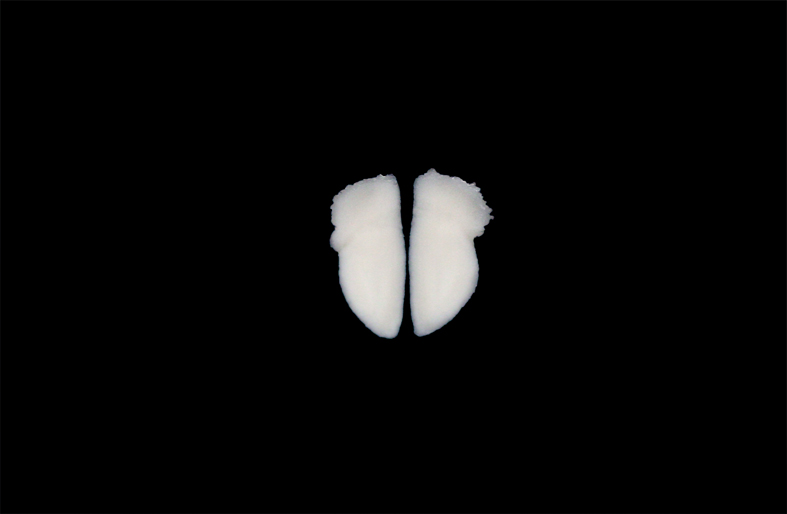

Supplement: Supplementary file 2 — Dataset 1 [file 41598_2019_55585_MOESM2_ESM.zip › 082.jpg]

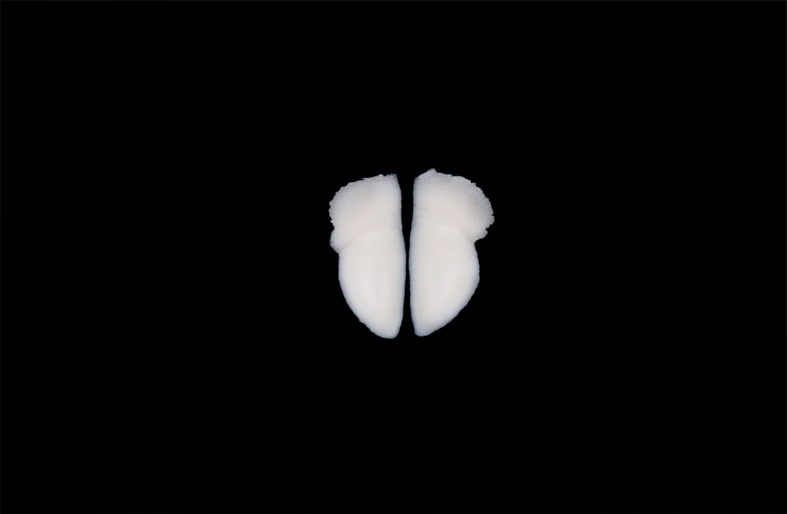

Supplement: Supplementary file 2 — Dataset 1 [file 41598_2019_55585_MOESM2_ESM.zip › 083.jpg]

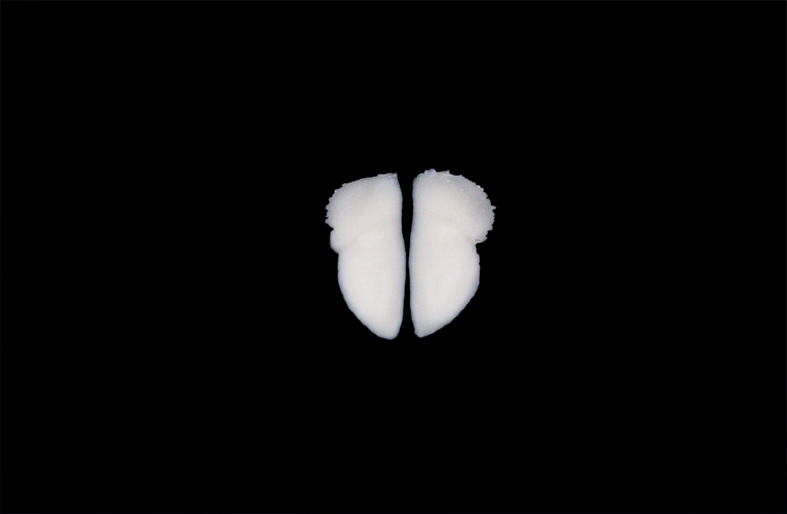

Supplement: Supplementary file 2 — Dataset 1 [file 41598_2019_55585_MOESM2_ESM.zip › 084.jpg]

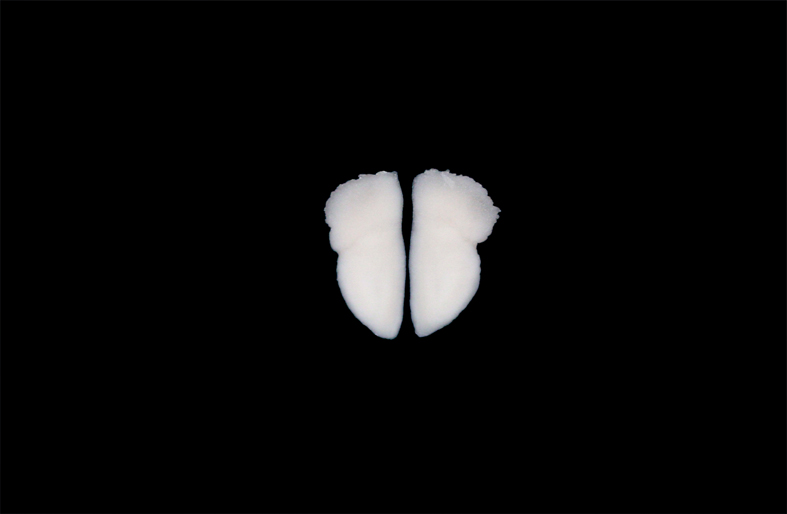

Supplement: Supplementary file 2 — Dataset 1 [file 41598_2019_55585_MOESM2_ESM.zip › 085.jpg]

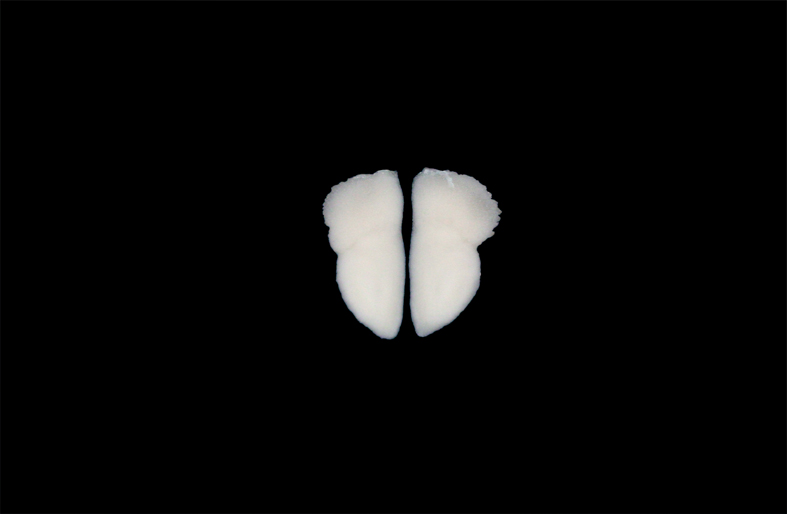

Supplement: Supplementary file 2 — Dataset 1 [file 41598_2019_55585_MOESM2_ESM.zip › 086.jpg]

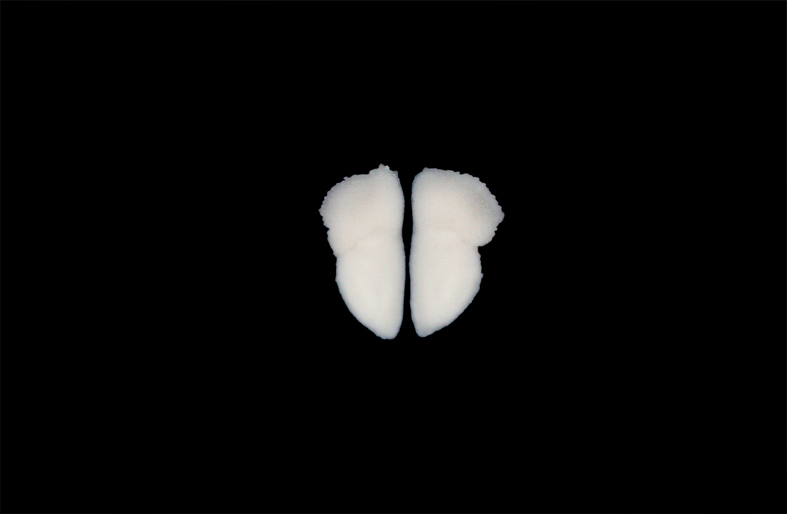

Supplement: Supplementary file 2 — Dataset 1 [file 41598_2019_55585_MOESM2_ESM.zip › 087.jpg]

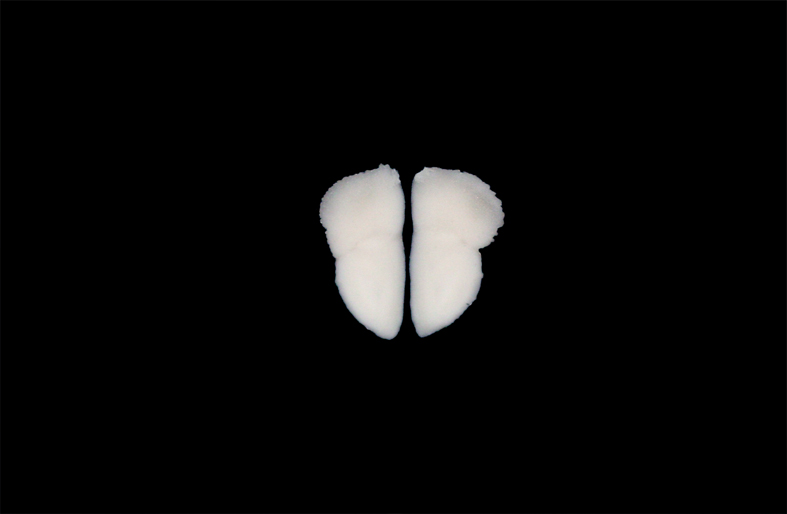

Supplement: Supplementary file 2 — Dataset 1 [file 41598_2019_55585_MOESM2_ESM.zip › 088.jpg]

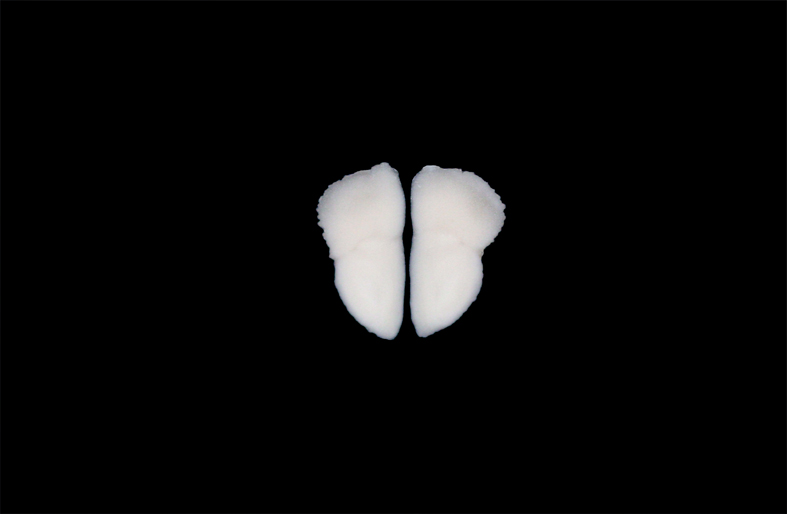

Supplement: Supplementary file 2 — Dataset 1 [file 41598_2019_55585_MOESM2_ESM.zip › 089.jpg]

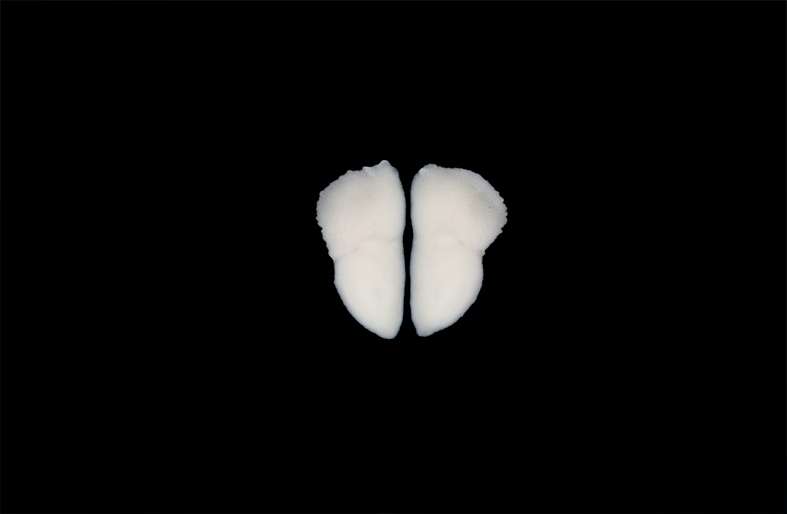

Supplement: Supplementary file 2 — Dataset 1 [file 41598_2019_55585_MOESM2_ESM.zip › 090.jpg]

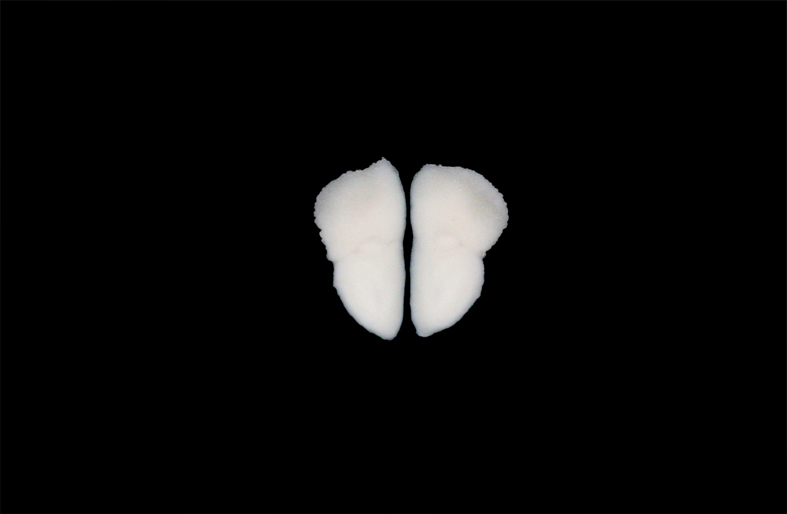

Supplement: Supplementary file 2 — Dataset 1 [file 41598_2019_55585_MOESM2_ESM.zip › 091.jpg]

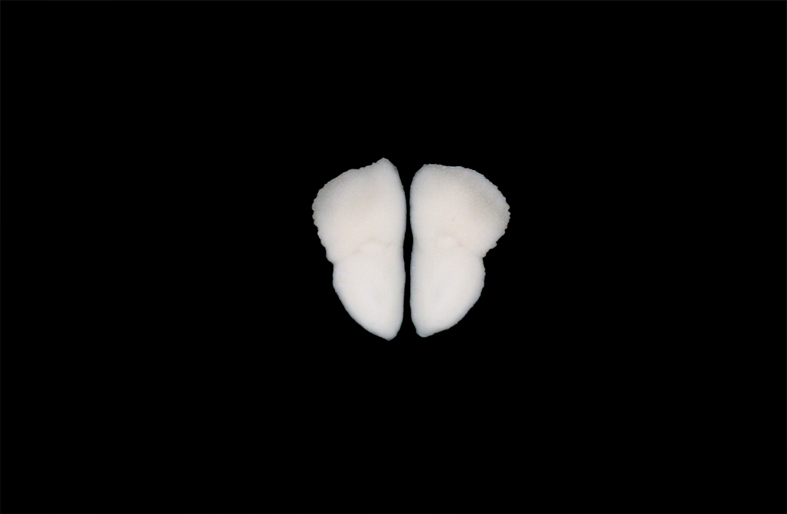

Supplement: Supplementary file 2 — Dataset 1 [file 41598_2019_55585_MOESM2_ESM.zip › 092.jpg]

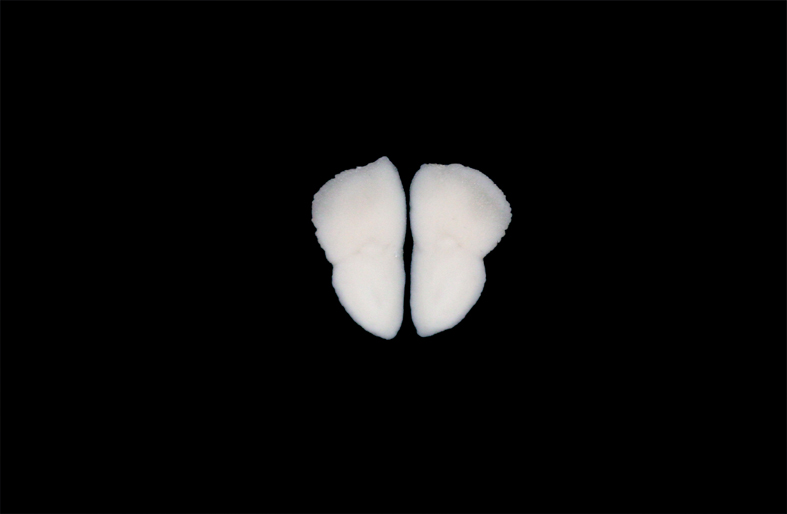

Supplement: Supplementary file 2 — Dataset 1 [file 41598_2019_55585_MOESM2_ESM.zip › 093.jpg]

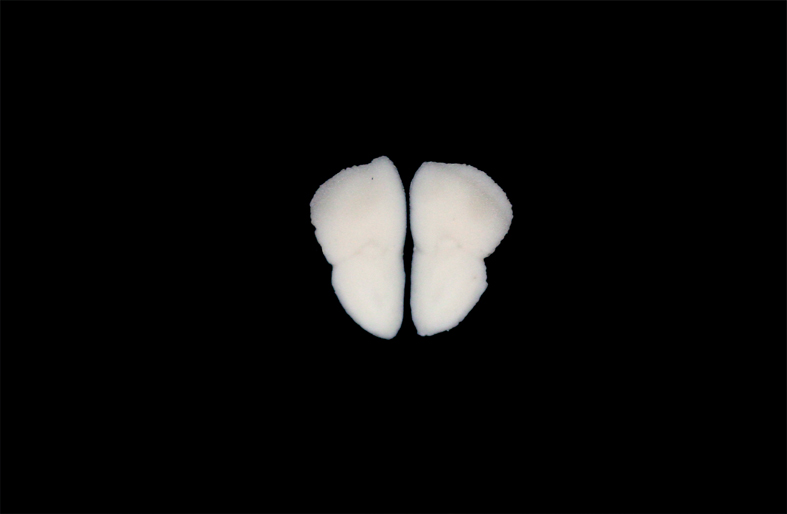

Supplement: Supplementary file 2 — Dataset 1 [file 41598_2019_55585_MOESM2_ESM.zip › 094.jpg]

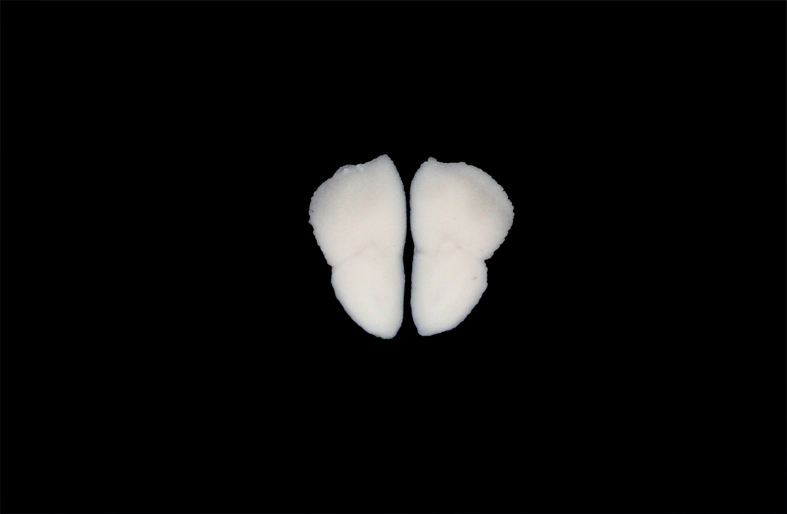

Supplement: Supplementary file 2 — Dataset 1 [file 41598_2019_55585_MOESM2_ESM.zip › 095.jpg]

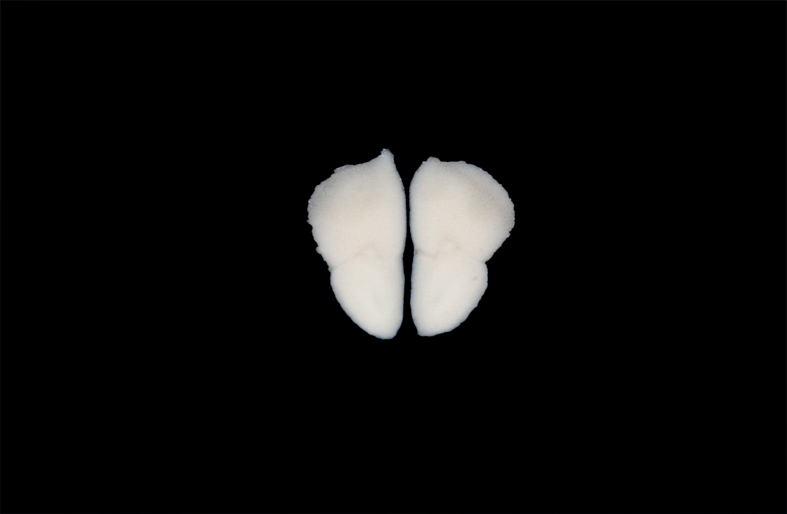

Supplement: Supplementary file 2 — Dataset 1 [file 41598_2019_55585_MOESM2_ESM.zip › 096.jpg]

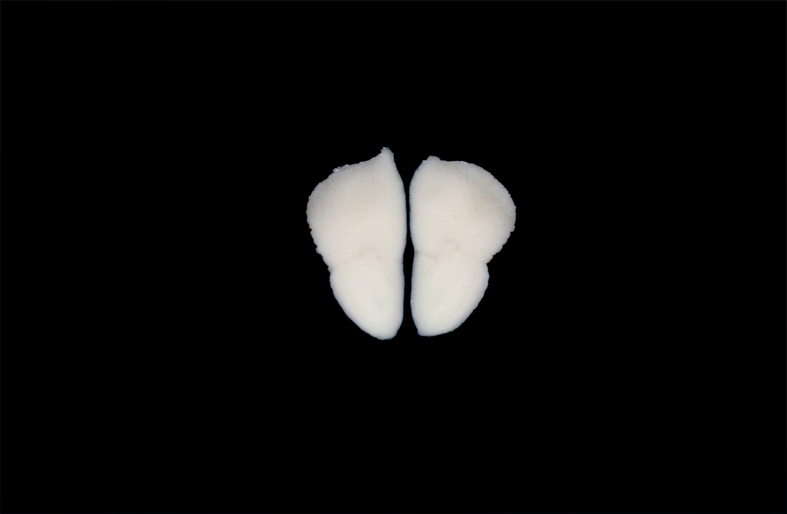

Supplement: Supplementary file 2 — Dataset 1 [file 41598_2019_55585_MOESM2_ESM.zip › 097.jpg]

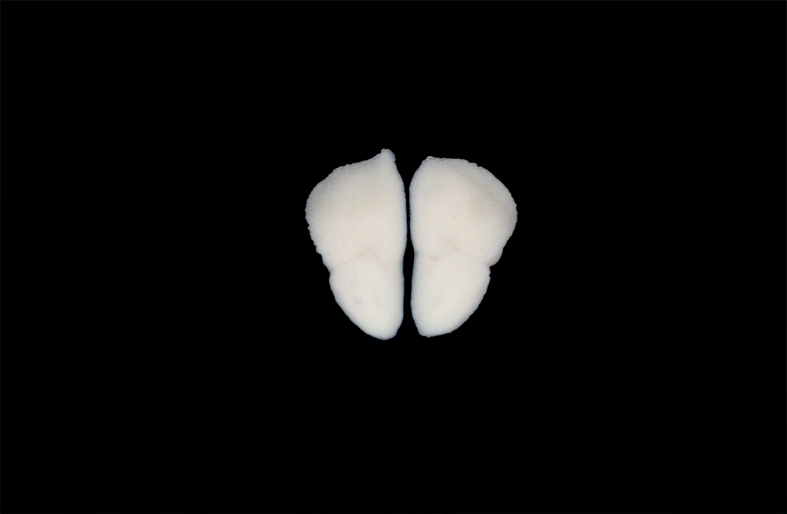

Supplement: Supplementary file 2 — Dataset 1 [file 41598_2019_55585_MOESM2_ESM.zip › 098.jpg]

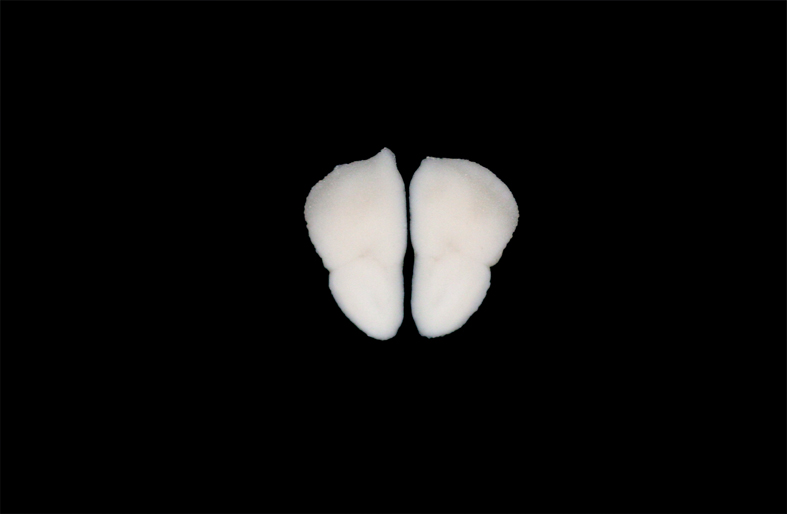

Supplement: Supplementary file 2 — Dataset 1 [file 41598_2019_55585_MOESM2_ESM.zip › 099.jpg]

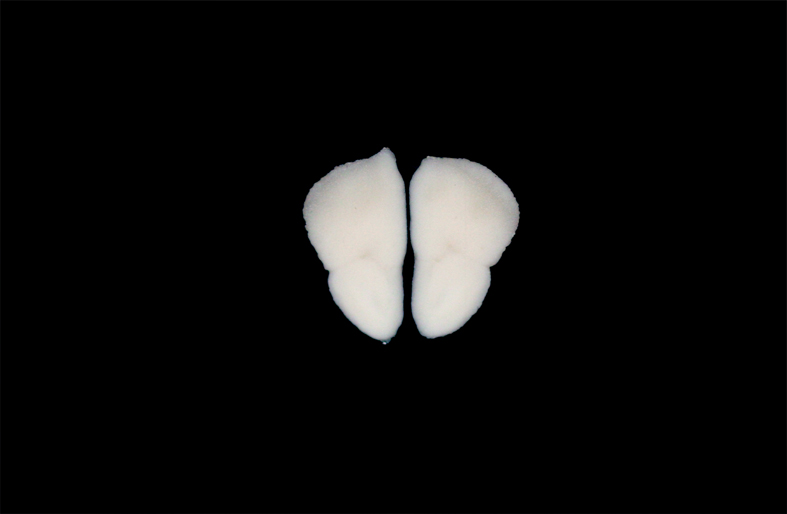

Supplement: Supplementary file 2 — Dataset 1 [file 41598_2019_55585_MOESM2_ESM.zip › 100.jpg]
